# Supplementary material for: HEATR5B associates with dynein‐dynactin and promotes motility of AP1‐bound endosomal membranes
Source: EMBO J. 2023 Oct 24;42(23):e114473. doi: 10.15252/embj.2023114473 (PMC10690479; doi:10.15252/embj.2023114473)
Supplement: Supplementary file 1 — Appendix [file EMBJ-42-e114473-s007.pdf]

APPENDIX

HEATR5B associates with dynein-dynactin and promotes motility of AP1-bound endosomal membranes

Madan et al.

Table of contents:

Appendix Figure S1. Purified protein samples.	2
Appendix Figure S2. Association of GFP-HEATR5B with AP1γ and RAB11A in live cells and validation of *DYNC1H1* siRNA.	3
Appendix Figure S3. Strategy for generating HEATR5B deficient human cells with CRISPR/Cas9.	4
Appendix Figure S4. Disruption of AP1γ localisation in additional *HEATR5B* mutant clonal U2OS cell lines and phenotypic rescue with *HEATR5B* cDNA.	5
Appendix Figure S5. Clustered RAB11A and AP1γ-positive structures in GFP-HATR5B overexpressing cells are in the vicinity of the TGN.	6
Appendix Figure S6. Generation of a *Drosophila Heatr5* mutant allele and analysis of the *Heatr5* zygotic and maternal phenotypes.	7
Appendix Figure S7. Supplementary results of CRISPR-based depletion of maternally provided Heatr5.	8
Appendix Figure S8. Supplementary information and results for AP1γ motility assays in *Drosophila* embryos.	9
Appendix Figure S9. Golgin-245 targeting strategy.	10
Appendix Figure S10. Supplementary information on protein localisation and trafficking in the *Drosophila* embryo.	11
Appendix Table S1. Non-dynein-dynactin components enriched on the dynein tail vs tag control in the absence of exogenous dynactin in either tail configuration	12
Appendix Table S2. Non-dynein-dynactin components enriched on the dynein tail vs tag control in the presence of exogenous dynactin in at least one tail configuration	14
Appendix Table S3. Information on *HEATR5B* mutant U2OS cell lines	15
Appendix Table S4. Oligonucleotides used to create gRNA plasmids for CRISPR in *Drosophila*	16

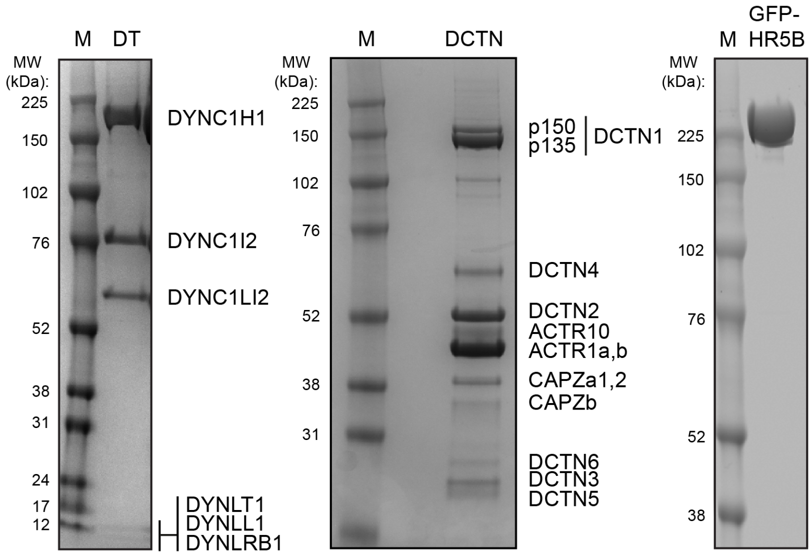

Appendix Figure S1 - Purified protein samples.

Images of Coomassie-stained gel lanes after electrophoresis of the human dynein tail complex (DT), pig brain dynactin (DCTN) and GFP tagged human HEATR5B (HR5B). Note that DCTN1 exists as two different isoforms (p150 and p135). M, protein markers; MW, molecular weight of protein markers.

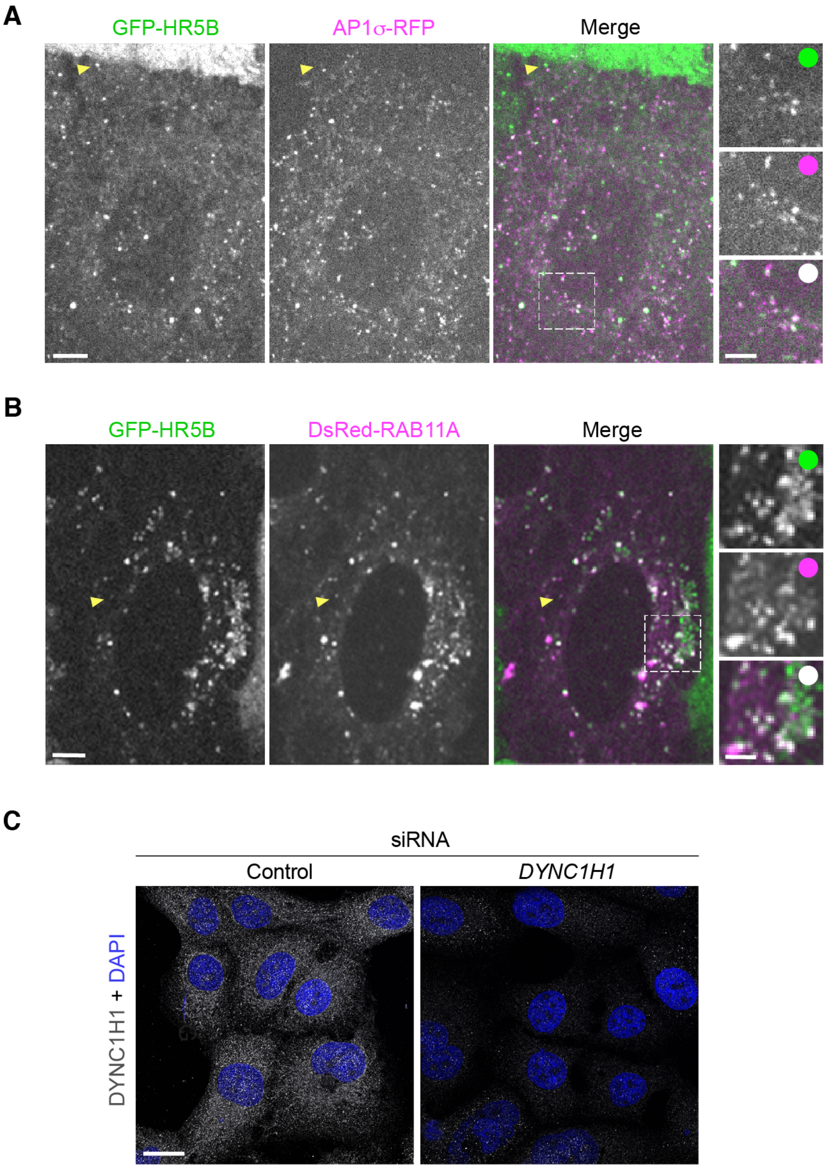

Appendix Figure S2 - Association of GFP-HEATR5B with AP1γ and RAB11A in live cells and validation of *DYNC1H1* siRNA.

A, B Representative spinning disk confocal images of HeLa cells that have a stable integration of a GFP-HEATR5B (HR5B) construct and have been transfected with AP1σ-RFP or DsRed-RAB11A expression plasmids. Dashed boxes show areas magnified in right-hand images. Arrowheads show particles highlighted in Movies EV2 and EV4 that undergo long-range transport.

C Representative confocal images of HeLa cells treated with control or *DYNC1H1* siRNAs and stained with a DYNC1H1 antibody, confirming effective knockdown of the protein with *DYNC1H1* siRNAs.

Data information: Scale bars: A and B, 5 µm; A and B insets, 2.5 µm; C, 20 µm.

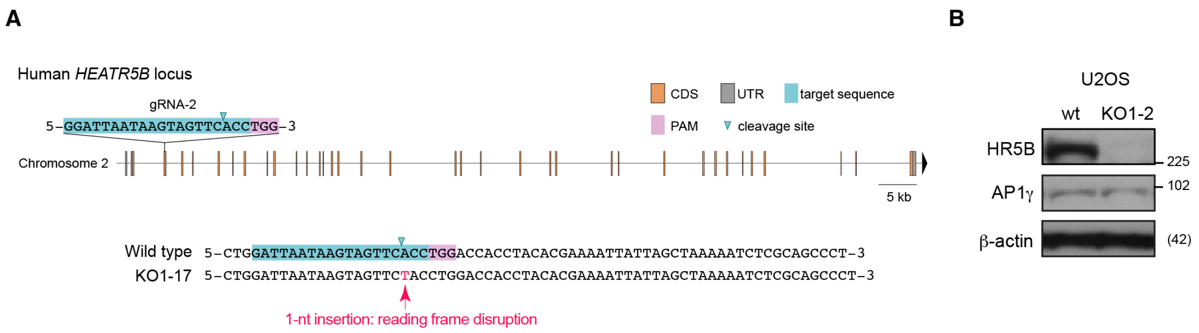

Appendix Figure S3 - Strategy for generating HEATR5B deficient human cells with CRISPR/Cas9.

A Position of target site of gRNA in human *HEATR5B* locus and sequence of U2OS clone 1-17 (which was used for mutant analysis unless stated otherwise) compared to the wild-type precursor. Information on indels in the other mutant cell lines is available in Appendix Table S3.

B Immunoblots showing loss of HEATR5B (HR5B) protein in U2OS KO1-2 clone. Equivalent data for KO1-17 clone is shown in Fig 4D.

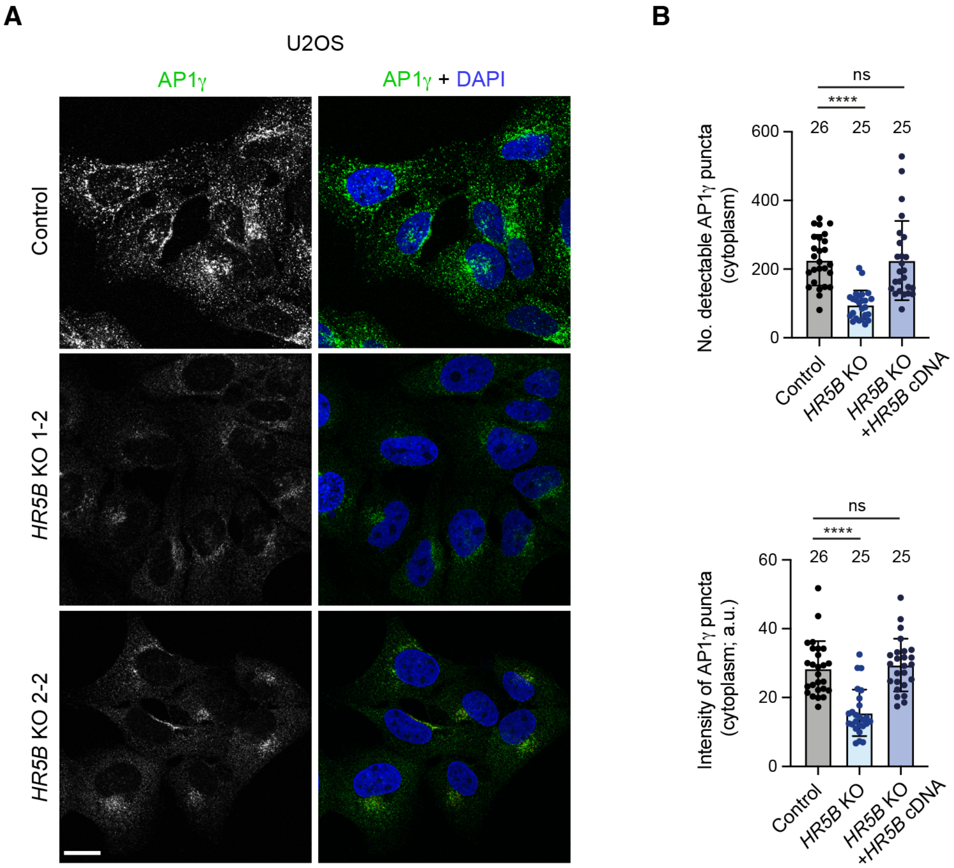

Appendix Figure S4 - Disruption of AP1γ localisation in additional *HEATR5B* mutant clonal U2OS cell lines and phenotypic rescue with *HEATR5B* cDNA.

A Representative confocal images of control (parental) and additional *HEATR5B* (*HR5B*)-mutant U2OS clonal lines. Scale bar: 15 μm.

B Quantification of number and mean total intensity of AP1γ puncta in control U2OS cells, *HR5B* KO U2OS cells and *HR5B* U2OS cells transfected with a GFP-HR5B expression plasmid (a.u., arbitrary units; see Figure EV2A for representative images for rescue condition). Circles indicate values from individual cells, with columns and error bars representing mean ± S.D. Numbers of cells analysed is shown above columns. Statistical significance was evaluated with a one-way ANOVA test with multiple comparisons correction. ****P <0.0001.

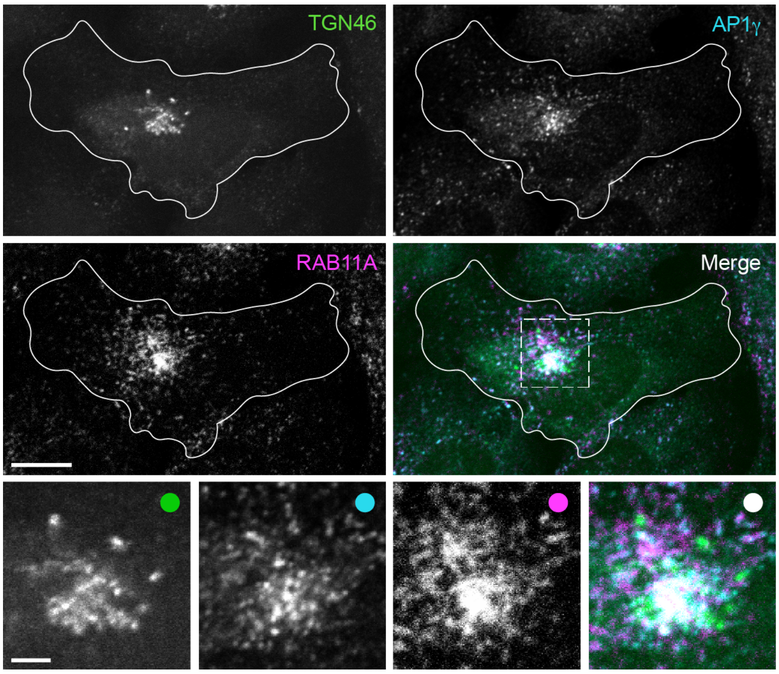

Appendix Figure S5 – Clustered RAB11A and AP1γ-positive structures in GFP-HEATR5B overexpressing cells are in the vicinity of the TGN.

Representative confocal images GFP-HEATR5B overexpressing cell stained with the indicated antibodies. White outline shows cell that expresses GFP-HR5B (as assessed by imaging the GFP channel). Dashed box shows area magnified in bottom row of images. Scale bar: 10 μm; insets, 2.5 μm.

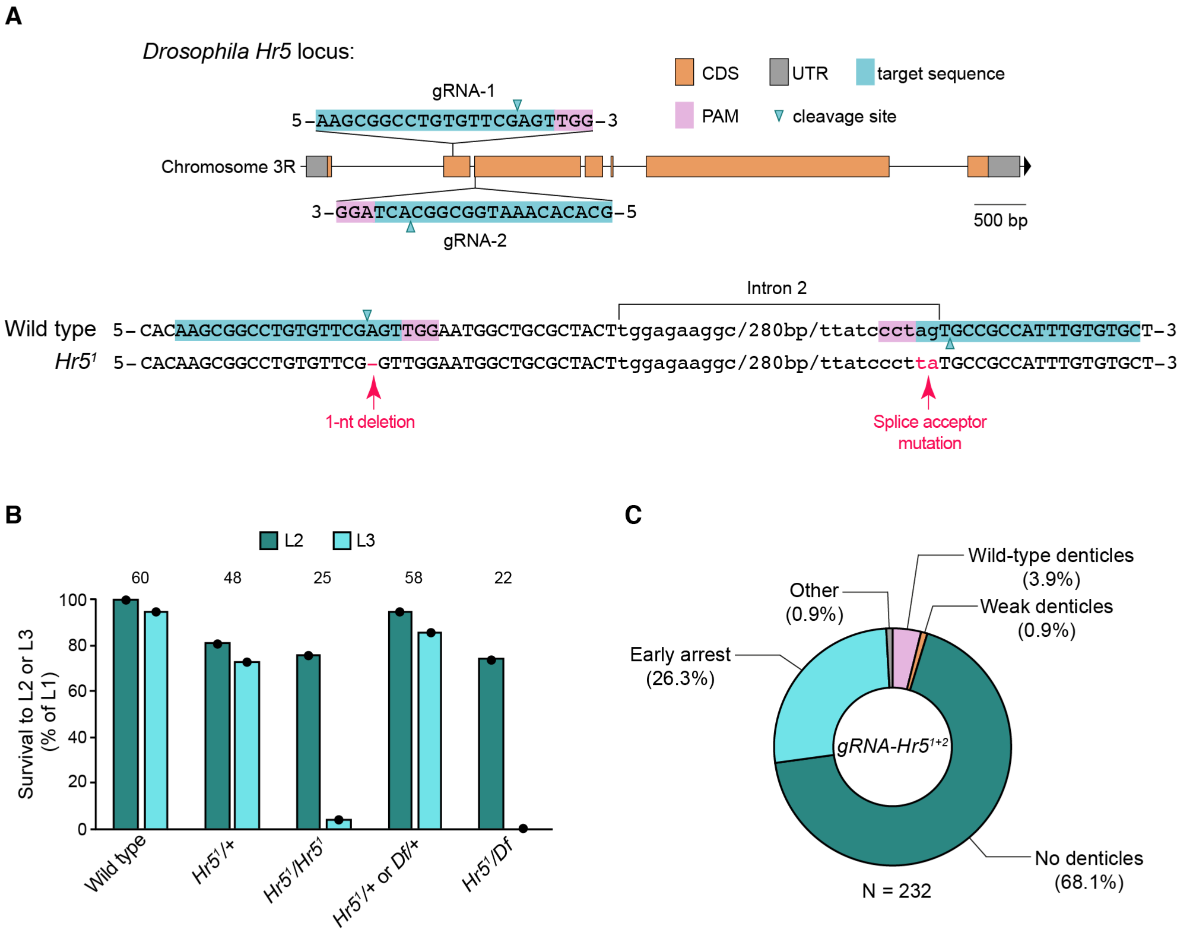

Appendix Figure S6 - Generation of a *Drosophila Heatr5* mutant allele and analysis of the *Heatr5* zygotic and maternal phenotypes.

A Position of target sites of *gRNA-Hr5*$^{1+2}$ transgene in *Heatr5 (Hr5)* locus (top) and sequence of the *Hr5*^{1} mutant allele compared to the wild-type precursor (bottom). PAM, protospacer adjacent motif. In the sequence alignment, the position of the PAM and target sequence is transposed to the opposite strand for simplicity.

B Lethal phase analysis of *Hr5*^{1} zygotic mutants. L1, L2 and L3 are successive larval instar stages. As none of the genotypes exhibited significant lethality during embryogenesis, only the rate of survival of L1 larvae of the indicated genotypes to L2 or L3 stages was recorded (*Df*: chromosomal deficiency *Df(3R)BSC222*, which uncovers the *Hr5* locus). Data are expressed as a percentage of initial number of L1 larvae for each genotype, with the number of L1 larvae followed for each genotype shown above columns. *Hr5*^{1}/+ and *Hr5*^{1} homozygous larvae were siblings from the same cross, as were '*Hr5*^{1}/+ or *Df/+*' and *Hr5*^{1}/*Df* larvae.

C Quantification of cuticle defects of unhatched embryos from *nos-cas9 gRNA-Hr5*$^{1+2}$ females. Embryos in the 'No denticles' category had reached late stages of embryogenesis as judged by the development of obvious internal structures, such as abdominal segments and/or mouth parts; 'Early arrest' embryos had no obvious internal structures; 'Other' represents rare instances of axial patterning defects. Data are pooled from egg lays of females generated in three independent crosses, across which the results were very consistent. N is number of embryos analysed.

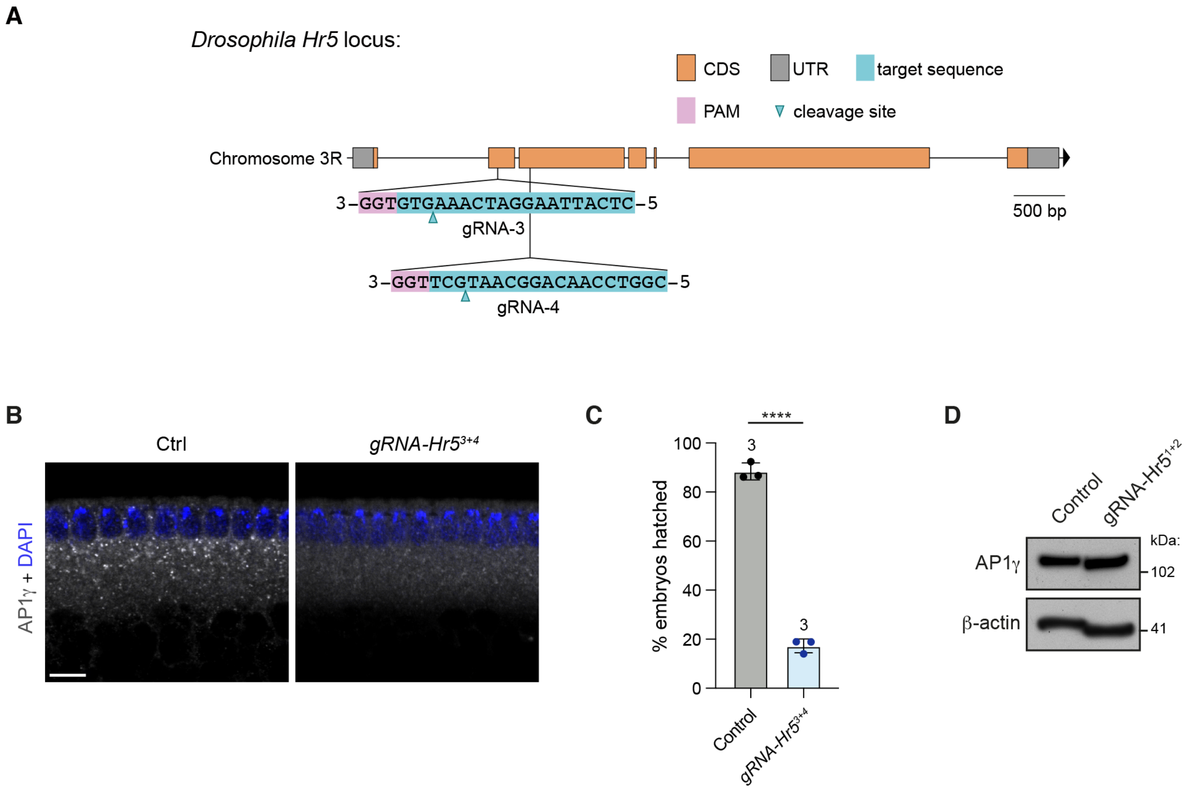

Appendix Figure S7 - Supplementary results of CRISPR-based depletion of maternally provided Heatr5.

A Position of target sites of *gRNA-Hr5*$^{3+4}$ transgene in the *Heatr5 (Hr5)* locus.

B Representative confocal images of embryos from control (*nos-cas9*) and *nos-cas9 gRNA-Hr5*$^{3+4}$ females stained with AP1γ antibodies. Scale bar, 10 μm.

C Hatching frequency of embryos laid by control (*nos-cas9*) and *nos-cas9 gRNA-Hr5*$^{3+4}$ females. Chart shows mean values per egg lay ± SD; circles are values for individual egg lays (at least 150 embryos analysed per egg lay); number of egg lays is shown above columns. Statistical significance was evaluated with a t-test: ****P <0.0001.

D Immunoblot images showing AP1γ protein level in cohorts of 0.5–3.5 h embryos laid by control (*nos-cas9*) and *nos-cas9 gRNA-Hr5*$^{1+2}$ females. β-actin was used as a loading control.

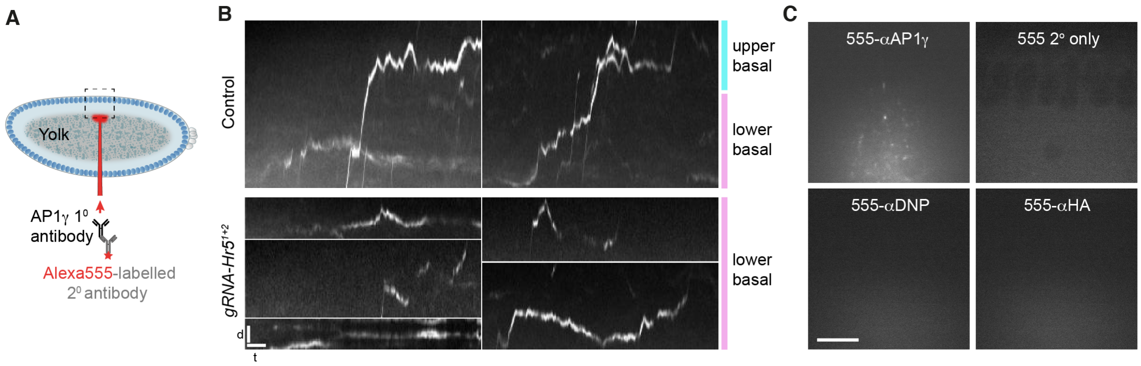

Appendix Figure S8 - Supplementary information and results for AP1γ motility assays in *Drosophila* embryos.

A Diagram of microinjection procedure. Dashed box shows typical field-of-view for imaging.

B Kymographs showing examples of AP1γ motility in embryos from control (*cas9*) and *nos-cas9 gRNA-Hr5*$^{1+2}$ females. Apical is to the top of each image. d = distance and t = time. Scale bars, 2 μm and 20 s.

C Representative images of embryos injected ~ 60 s earlier with the indicated antibodies; note the absence of bright puncta for all antibodies except α-AP1γ. Scale bar, 10 μm.

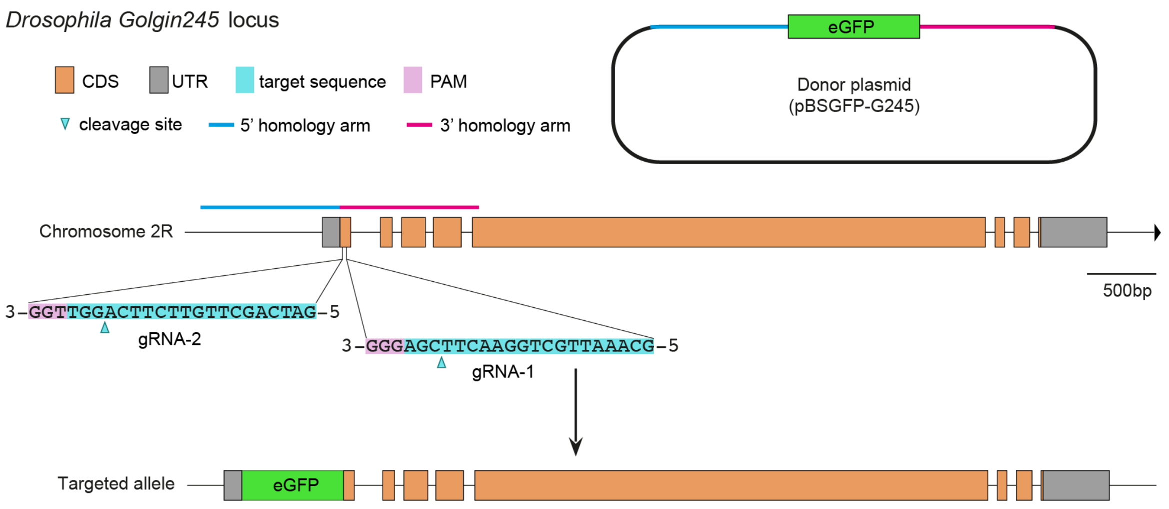

Appendix Figure S9 - Golgin-245 targeting strategy.

Illustration of target sites and homology arms in the *Golgin245* locus, the GFP donor construct, and the *Golgin245* locus after knock-in of GFP.

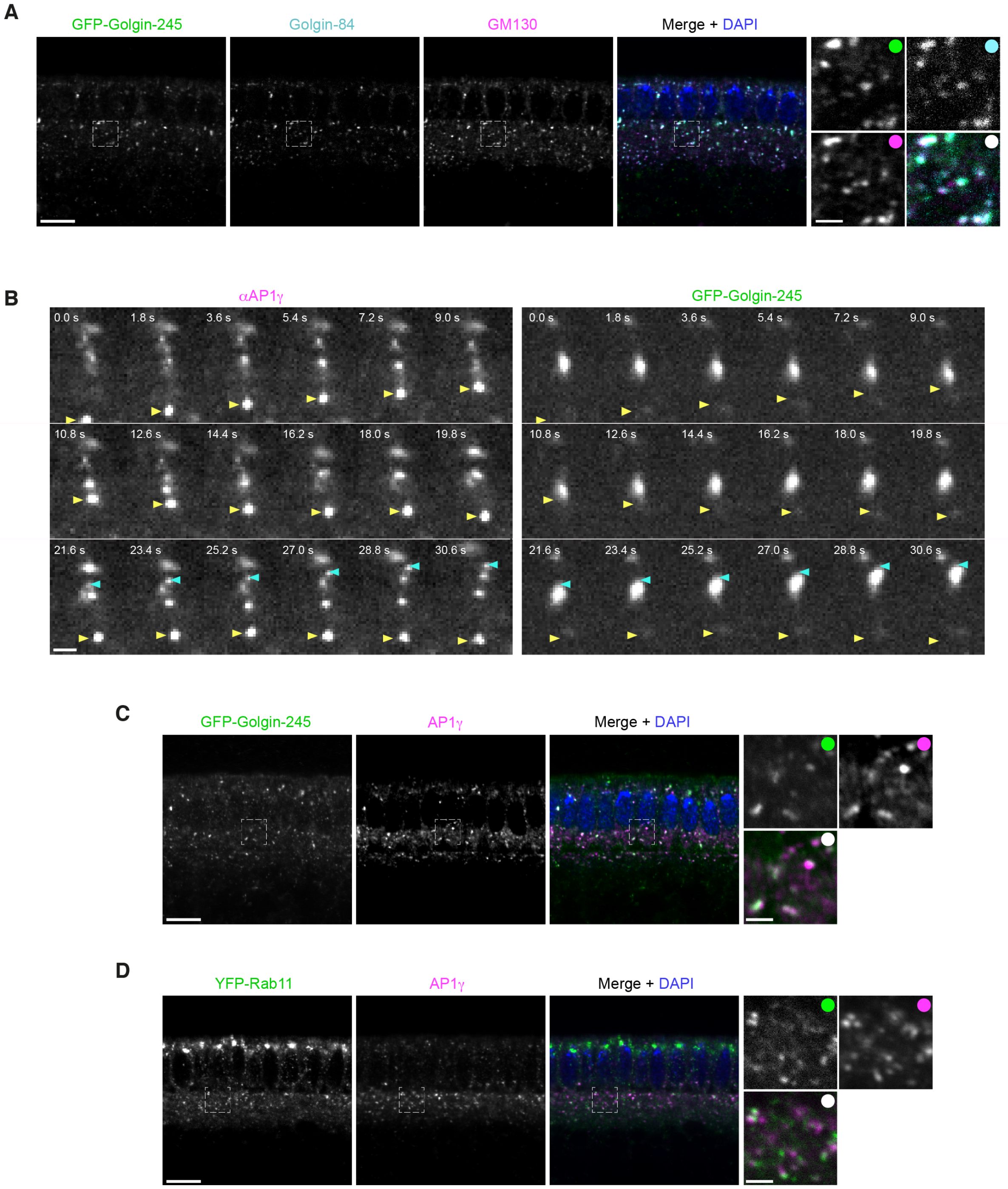

Appendix Figure S10 - Supplementary information on protein localisation and trafficking in the *Drosophila* embryo.

A Representative confocal images of blastoderm embryo stained for GFP-Golgin-245, Golgin-84 and GM130 showing the GFP fusion protein localises to the Golgi.

B Representative single-channel stills corresponding to the image series from Figure 7A (with yellow and cyan arrowheads in the equivalent positions).

C, D Representative confocal images of wild-type blastoderm embryos stained for GFP-Golgin-245 and AP1γ (C) or YFP-Rab11 and AP1γ (D). Dashed boxes show areas magnified in right-hand images. Scale bars: A, C and D, 10 μm; B, and A, C, D insets, 2 μm.

Appendix Table S1. Non-dynein-dynactin components enriched on the dynein tail vs tag control in the absence of exogenous dynactin in either tail configuration

Protein	Function	Identifier [1]	Polypeptide length (aa)	Position(s) of predicted coiled coil(s) [2]
Arglu1	RNA binding/ transcriptional regulation	Q3UL36	271	-
Asap3	Arf GTPase activating factor	Q5U464	904	142-167, 249-273
Bicd2	Dynein-dynactin cargo adaptor	Q921C5	820	20-270, 340-539, 662-804
Btf3l4	Transcriptional regulation	Q9CQH7	158	-
Hist2h2bb	Nucleosome component	Q64525	126	-
Larp7	RNA binding/ snRNA processing	Q05CL8	570	-
Luc7l	Putative RNA binding	Q9CYI4	371	87-177, 220-256
Manf	Neurotrophic factor	Q9CXI5	179	-
Mapk8ip3	Dynein-dynactin and kinesin cargo adaptor (Jip3)	Q9ESN9	1337	58-177, 437-555
Nde1	Regulator of dynein and Lis1	Q9CZA6	344	18-188
Pdxk	Vitamer kinase	Q8K183	312	-
Pfn2	Actin binding	Q9JJV2	140	-
Pkfl	ATP-dependent 6-phosphofructokinase	P12382	780	-
Ppp3R1	Calcineurin subunit	Q63810	170	-
Prdx2	Thiol-specific peroxidase	Q61171	198	-
Psd3	Arf6 GEF	Q2PFD7	1037	911-941
Pura	Nucleic acid binding	P42669	321	-
Purb	Nucleic acid binding	O35295	324	-
Rpl7a	Large ribosomal subunit	P12970	266	-
Rpl10a	Large ribosomal subunit	P53026	217	-
Rpl12	Large ribosomal subunit	P35979	165	-
Rpl17	Large ribosomal subunit	Q9CPR4	184	-

Rpl18	Large ribosomal subunit	P35980	188	-
Rpl31	Large ribosomal subunit	P62900	125	-
Rplp1	Translational elongation	P47955	114	-
Rps10	Small ribosomal subunit	P63325	165	-
Rps13	Small ribosomal subunit	P62301	151	-
Rps18	Small ribosomal subunit	P62270	152	-
Rps23	Small ribosomal subunit	P62267	143	-
Rps28	Small ribosomal subunit	P62858	69	-
Rsrc2	Cell proliferation regulator	A2RTL5	376	171-214
Slc35g2	Solute carrier	D3YVE8	412	-
Snrnp70	Spliceosomal U1 snRNP component	Q62376	448	-
Snrpd1	Spliceosomal snRNP component	P62315	119	-
Tma7	Unknown	Q8K003	64	21-50

(1) UniProtKB accession number (canonical mouse sequence)
(2) Information compiled at UniProtKB (https://www.uniprot.org)

Appendix Table S2. Non-dynein-dynactin components enriched on the dynein tail vs tag control in the presence of exogenous dynactin in at least one tail configuration

Protein	Molecular function	Identifier [(1)]	Polypeptide length (aa)	Position(s) of predicted coiled coil(s) [(2)]
Acp1	Phosphotyrosine protein phosphatase	Q9D358	158	-
Calm1	Calcium-based enzyme regulation	P0DP26	149	-
Cttnbp2	Regulates neuronal cortactin distribution	B9EJA2	1648	119-274
Gfap	Intermediate filament protein	P03995	430	70-214, 228-374
Heatr5B	AP-1 complex associated	Q8C547	2070	-
Hnrnpu	DNA and RNA binding protein	Q8VEK3	800	626-653
Ppm1e	Protein phosphates	Q80TL0	749	-
Rpl14	Large ribosomal subunit protein	Q9CR57	217	-
Rpl19	Large ribosomal subunit protein	P84099	196	-
Rpl6	Large ribosomal subunit protein	P47911	296	-
Rps7	Small ribosomal subunit protein	P62082	194	-
Rps8	Small ribosomal subunit protein	P62242	208	-
Rps16	Small ribosomal subunit protein	P14131	146	-
Smarca2	Transcriptional regulation	Q6DIC0	1577	-
Strip1	Cytoskeletal regulator	Q8C079	837	-
Tial1	RNA binding	P70318	392	-
Wdr91	Endosomal trafficking	Q7TMQ7	748	-

(1) UniProtKB accession number (canonical mouse sequence)
(2) Information compiled at UniProtKB (https://www.uniprot.org)

Appendix Table S3. Information on *HEATR5B* mutant U2OS cell lines

Clone name	Types of mutation detected (nt) (1)	Protein expression (2)
KO 1-17	+1	Abolished
KO 1-2	+1, -1	Abolished
KO 2-2	-20	Abolished

(1) Predominant alleles identified by TIDE; + and – refer, respectively, to insertion and deletions. In cases in which only one allele is detect, mutations may be homozygous or *trans*-heterozygous over an allele that deletes the sequenced region.
(2) Assessed by immunoblotting for full-length protein

Appendix Table S4. Oligonucleotides used to create gRNA plasmids for CRISPR in *Drosophila*

Gene	Constructed plasmid (1)	gRNA cloning oligos (2)
Heatr5	*pCDF4-gRNA-Hr5*$^{1+2}$	f:TATATAGGAAAGATATCCGGGTGAACTTCGAAGCGGCCTGTGTTCGAGTGTTTTAGAGCTAGAAATAGCAAG<br>r:ATTTTAACTTGCTATTTCTAGCTCTAAAACAGTGCCGCCATTTGTGTGCCGACGTTAAATTGAAAATAGGTC
Heatr5	*pCDF4-gRNA-Hr5*$^{3+4}$	f:TATATAGGAAAGATATCCGGGTGAACTTCGCTCATTAAGGATCAAAGTGGTTTTAGAGCTAGAAATAGCAAG<br>r:ATTTTAACTTGCTATTTCTAGCTCTAAAACAGCATTGCCTGTTGGACCGCGACGTTAAATTGAAAATAGGTC
Golgin245	*pCFD3-gRNA-G245*^{1}	f: GTCGCAAATTGCTGGAACTTCGA<br>r: AAACTCGAAGTTCCAGCAATTTG
	pCFD3-gRNA-G245^{2}	f: GTCGATCAGCTTGTTCTTCAGGT<br>r: AAACACCTGAAGAACAAGCTGAT

(1) See Port et al. (2014) for details of vectors.
(2) f, forward; r, reverse. Sequences corresponding to target sequence are underlined. For pCFD4, PCR with the primer pairs using the parental pCFD4 plasmid as a template yields a double gRNA containing product that can be used to produce the desired pCFD4 plasmid. For pCFD3, the primer pairs are annealed before cloning into the Bbs1-cut vector.
