# Supplementary material for: HEATR5B associates with dynein‐dynactin and promotes motility of AP1‐bound endosomal membranes
Source: EMBO J. 2023 Oct 24;42(23):e114473. doi: 10.15252/embj.2023114473 (PMC10690479; doi:10.15252/embj.2023114473)
Supplement: Supplementary file 2 — Expanded View Figures PDF [file EMBJ-42-e114473-s006.pdf]

Expanded View Figures

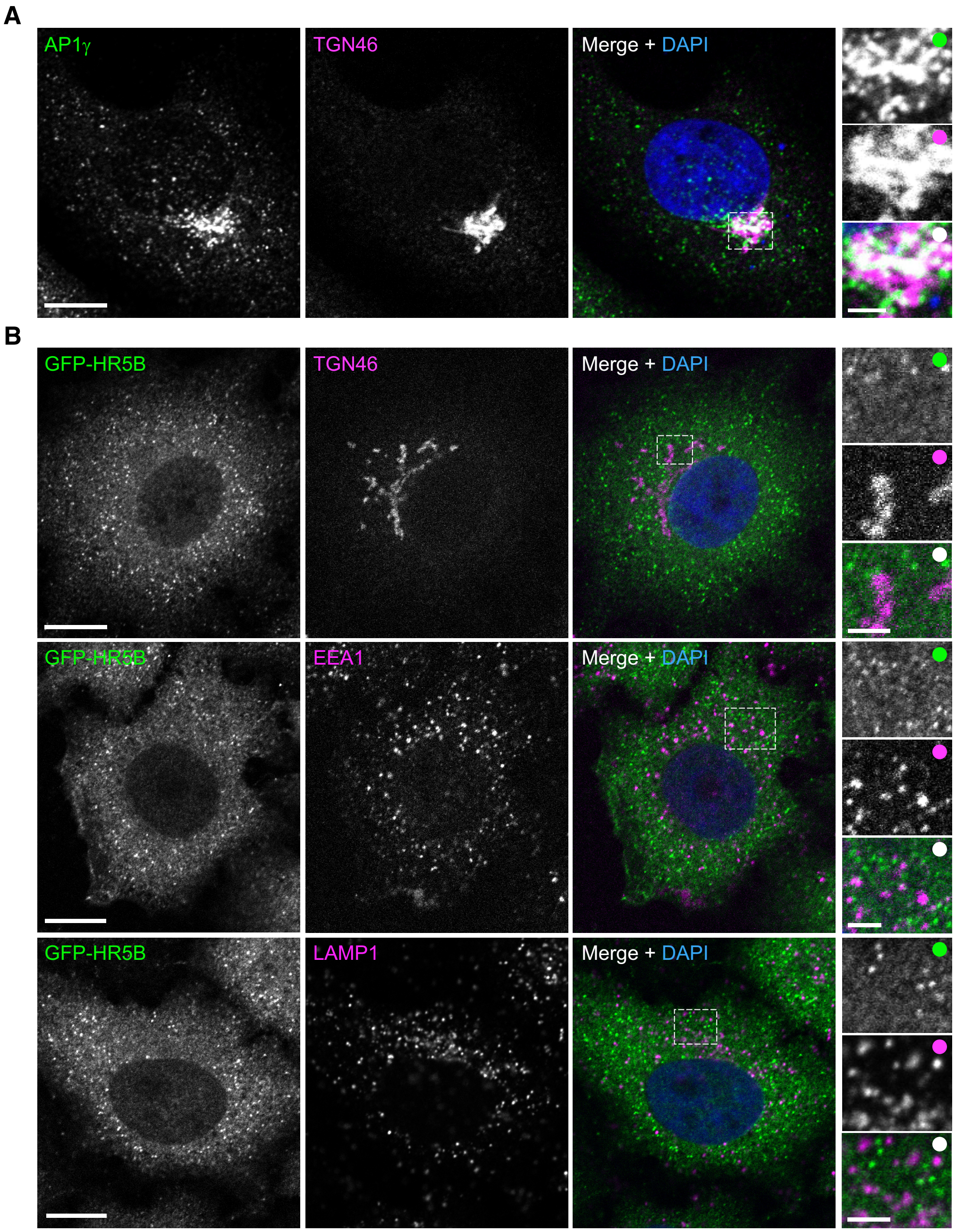

Figure EV1. Localisation of AP1γ and GFP-HEATR5B with respect to other structures in fixed human cells.

A, B Representative confocal images of wild-type (A) and stable GFP-HEATR5B (B) HeLa cells stained with antibodies to the indicated proteins (GFP signal in panel (B) was amplified with GFP antibodies). Dashed box shows area magnified in right-hand images. Panel (A) shows that AP1γ is clustered at the periphery of the TGN. Panel (B) shows that association of GFP-HEATR5B (HR5B) with TGN46, EEA1 and LAMP1 is rarely observed. Scale bars: main panels, 10 μm; insets, 2.5 μm. White circles indicate merge of magnified images.

 © 2023 The Authors

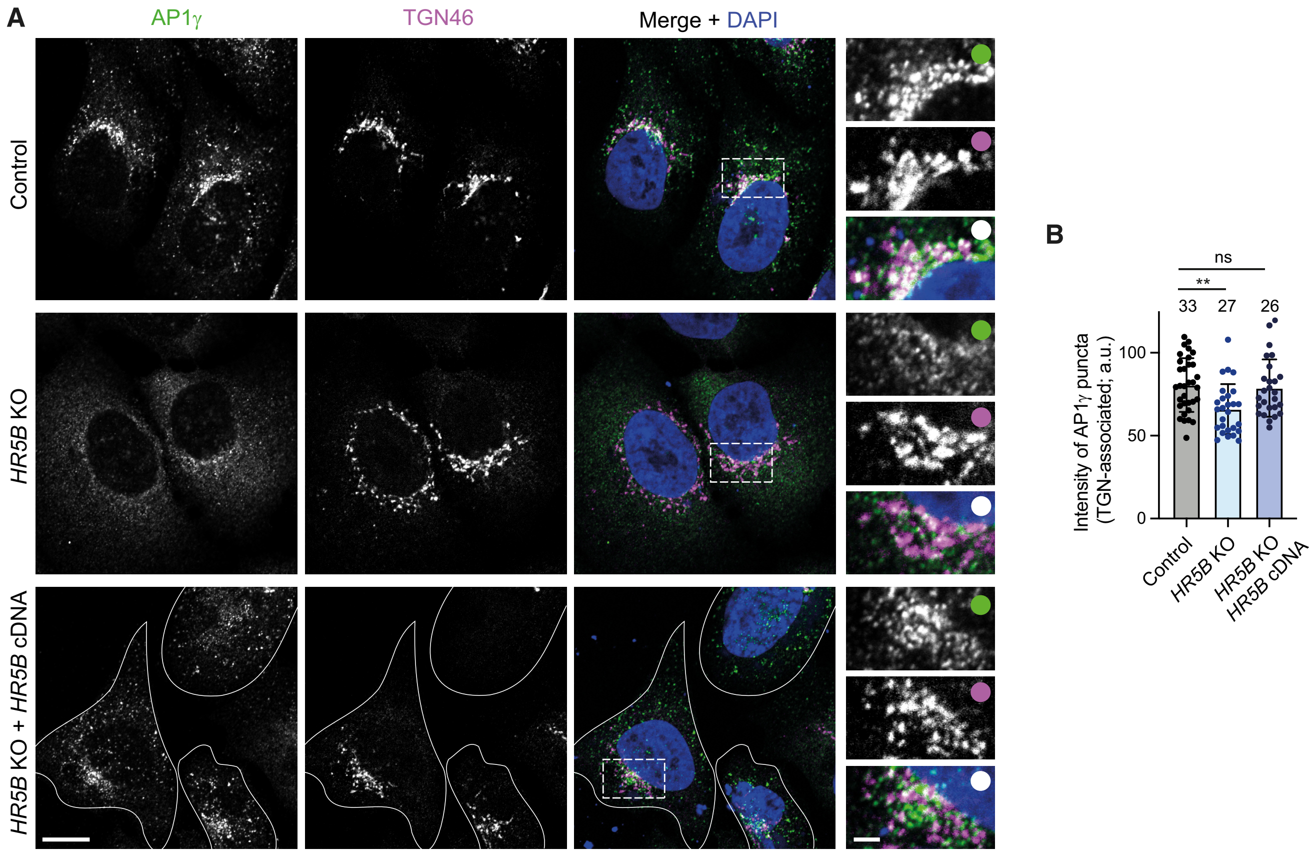

Figure EV2. Disruption of HEATR5B partially impairs association of AP1γ with the TGN.

A Representative confocal images of control (parental) U2OS cells, *HR5B* KO U2OS cells and *HR5B* KO U2OS cells transfected with a GFP-HR5B expression plasmid stained with antibodies to AP1γ and TGN46. For illustrative purposes, the *HR5B* KO cells shown are amongst those with a strong reduction in TGN-associated AP1γ signal. Dashed box shows area magnified in right-hand images. White outlines show mutant cells that express GFP-HR5B (as assessed by imaging the GFP channel). Scale bars: (A) 10 μm; (A) insets: 2.5 μm. White circles indicate merge of magnified images.

B Quantification of mean intensity of TGN-associated AP1γ puncta. Circles indicate values from individual cells, with columns and error bars representing mean ± SD. Number of cells analysed is shown above columns. Statistical significance was evaluated with a one-way ANOVA test with Dunnett's multiple comparisons correction: $^{**}P < 0.01$.

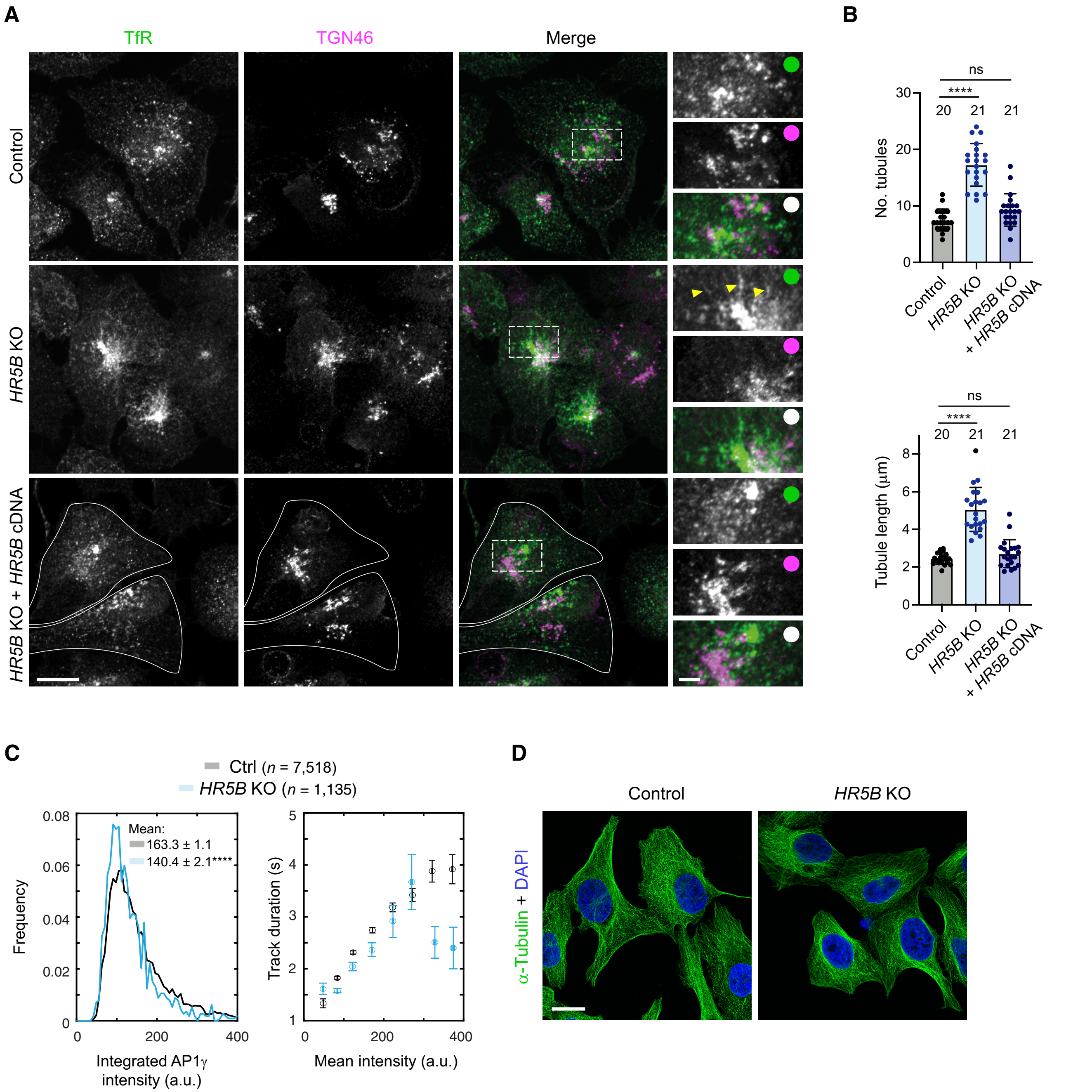

Figure EV3. Supplementary data on *HEATR5B* KO phenotypes.

A Representative confocal images of control (parental) U2OS cells, *HR5B* KO U2OS cells and *HR5B* KO U2OS cells transfected with a GFP-HR5B expression plasmid stained with antibodies to Transferrin receptor (TfR), which marks the recycling compartment in association with the TGN, and TGN46. Dashed box shows area magnified in right-hand images. Arrowheads show examples of tubulation in *HR5B* KO cells. White outlines show mutant cells that express GFP-HR5B (as assessed by imaging the GFP channel). White circles indicate merge of magnified images.

B Quantification of number and length of TfR-positive, TGN-associated tubules. Circles indicate values from individual cells, with columns and error bars representing mean ± SD. Number of cells analysed is shown above columns. Statistical significance was evaluated with a one-way ANOVA test with Dunnett's multiple comparisons correction. ****$P < 0.0001$.

C Quantification of AP1σ1-RFP particle intensity (left) and track duration versus mean particle intensity (right) in image series of control and *HR5B* KO live U2OS cells. n = number of particles (from 31 control and 17 KO U2OS cells). Errors represent SEM. In left-hand panel, a shift in the distribution of intensity values to the left in mutant cells indicates dimmer fluorescence (statistical significance was evaluated with a Mann–Whitney U test. ****$P < 0.0001$).

D Representative confocal images of control (parental) and *HR5B* KO U2OS cells stained with α-tubulin antibodies, showing no overt difference in the architecture of the microtubule cytoskeleton.

Data information: Scale bars: (A) 15 μm; (A) insets, 3 μm; (D) 20 μm.

 © 2023 The Authors

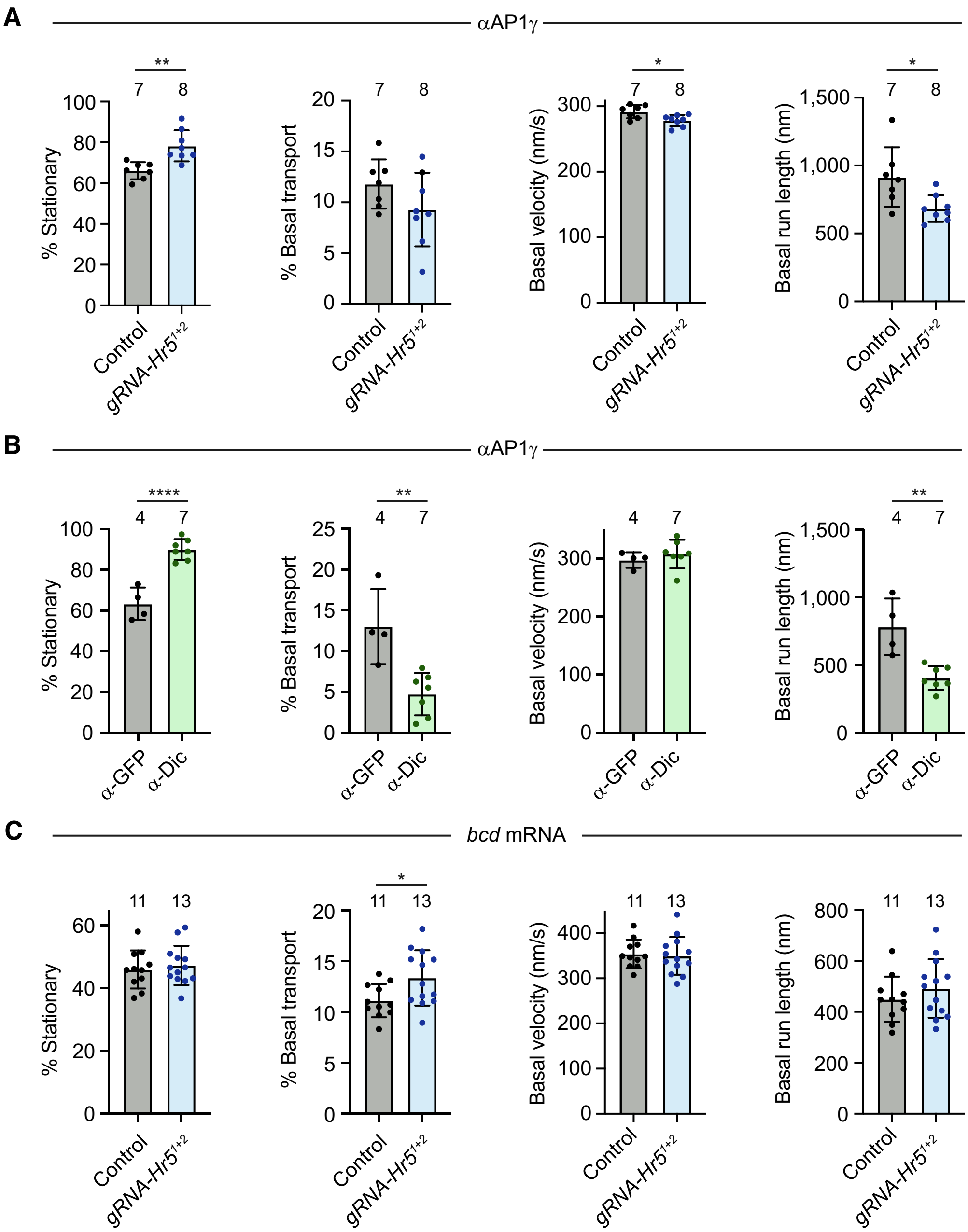

Figure EV4. Quantification of frequency of stationary events and basal transport in AP1γ and bcd mRNA motility assays.

A–C Quantification of the indicated parameters for AP1γ (A, B) and *bcd* mRNA (C) in embryos of control (*nos-cas9*) and *nos-cas9*, *gRNA-Hr5*$^{1+2}$ mothers (A, C) or wild-type embryos pre-injected with function-blocking Dic antibodies or control GFP antibodies (B). "% stationary" and "% basal" are the percentages of particle trajectory time that are classed as immobile or undergoing basal transport, respectively. Circles are mean values for individual embryos; columns and error bars represent means ± SD of these mean values; numbers of embryos injected is shown above columns. At least 24 particles were analysed per embryo. Statistical significance was evaluated with an unpaired two-tailed *t*-test: ****$P < 0.0001$; **$P < 0.01$; *$P < 0.05$.

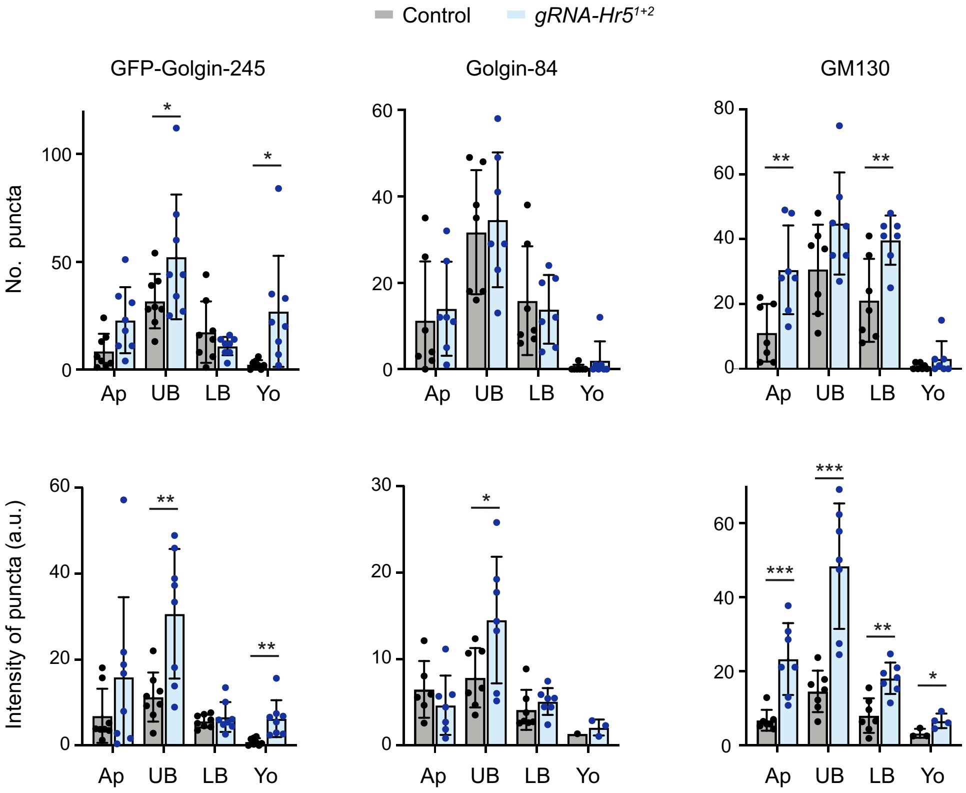

Figure EV5. Quantification of Golgi protein localisation in control and *Heatr5* mutant blastoderm embryos.

Charts show values for number of puncta and intensity of puncta for the indicated golgin proteins in different regions of the cytoplasm of embryos from control (*nos-cas9) and nos-cas9, gRNA-Hr5*$^{1+2}$ *mothers (Ap, apical to the nuclei; UB, upper basal region; LB, lower basal region; Yo, yolk). Columns and error bars show mean of values per embryo ± SD; circles show mean values for individual embryos. At least 50 puncta were analysed per embryo. Statistical significance was evaluated with an unpaired two-tailed t-test: ***P < 0.001; **P < 0.01; *P < 0.05. Consistent phenotypes were observed in two biological replicates; quantification is from a single biological replicate.*

 © 2023 The Authors
