# Supplementary material for: HEATR5B associates with dynein‐dynactin and promotes motility of AP1‐bound endosomal membranes
Source: EMBO J. 2023 Oct 24;42(23):e114473. doi: 10.15252/embj.2023114473 (PMC10690479; doi:10.15252/embj.2023114473)
Supplement: Supplementary file 3 — Movie EV1 [file EMBJ-42-e114473-s016.zip › Movie_EV1/Movie_EV1.docx]

**Movie EV1. Dual colour movie of HeLa cell expressing GFP-HEATR5B and AP1σ1-RFP.** In this and other movies, time in seconds is shown above scale bar (movie is looped). Scale bar, 10 μm.
