# Supplementary material for: HEATR5B associates with dynein‐dynactin and promotes motility of AP1‐bound endosomal membranes
Source: EMBO J. 2023 Oct 24;42(23):e114473. doi: 10.15252/embj.2023114473 (PMC10690479; doi:10.15252/embj.2023114473)
Supplement: Supplementary file 4 — Movie EV2 [file EMBJ-42-e114473-s021.zip › Movie_EV2/Movie_EV2.docx]

**Movie EV2. Example of long-distance co-transport of GFP-HEATR5B and AP1σ1-RFP in HeLa cell cytoplasm (crop of time series used to produce Movie EV1).** Shown is a composite of individual channels and the merge. Yellow arrow shows particle that will undergo long-distance movement. Nucleus is positioned to the bottom right of the frames. Scale bar, 2 μm.
