# Supplementary material for: HEATR5B associates with dynein‐dynactin and promotes motility of AP1‐bound endosomal membranes
Source: EMBO J. 2023 Oct 24;42(23):e114473. doi: 10.15252/embj.2023114473 (PMC10690479; doi:10.15252/embj.2023114473)
Supplement: Supplementary file 5 — Movie EV3 [file EMBJ-42-e114473-s026.zip › Movie_EV3/Movie_EV3.docx]

**Movie EV3. Dual colour movie of HeLa cell expressing GFP-HEATR5B and dsRed-RAB11A.** Movie is looped. Scale bar, 5 μm.
