# Supplementary material for: HEATR5B associates with dynein‐dynactin and promotes motility of AP1‐bound endosomal membranes
Source: EMBO J. 2023 Oct 24;42(23):e114473. doi: 10.15252/embj.2023114473 (PMC10690479; doi:10.15252/embj.2023114473)
Supplement: Supplementary file 6 — Movie EV4 [file EMBJ-42-e114473-s019.zip › Movie_EV4/Movie_EV4.docx]

**Movie EV4. Example of long-distance co-transport of GFP-HEATR5B and dsRed-RAB11A in HeLa cell cytoplasm (crop of time series used to produce Movie EV3).** Shown is a composite of individual channels and the merge. Yellow arrow shows particle that will undergo long-distance movement. Nucleus is positioned at the bottom of the frames. Scale bar, 5 μm.
