# Supplementary material for: HEATR5B associates with dynein‐dynactin and promotes motility of AP1‐bound endosomal membranes
Source: EMBO J. 2023 Oct 24;42(23):e114473. doi: 10.15252/embj.2023114473 (PMC10690479; doi:10.15252/embj.2023114473)
Supplement: Supplementary file 7 — Movie EV5 [file EMBJ-42-e114473-s025.zip › Movie_EV5/Movie_EV5.docx]

**Movie EV5. Behaviour of AP1σ1-RFP in control and *HEATR5B* KO U2OS cells.** Image series from movies with overlaid tracks. Note that the control movie has two cells in the field of view. Scale bar, 10 μm.
