# Supplementary material for: HEATR5B associates with dynein‐dynactin and promotes motility of AP1‐bound endosomal membranes
Source: EMBO J. 2023 Oct 24;42(23):e114473. doi: 10.15252/embj.2023114473 (PMC10690479; doi:10.15252/embj.2023114473)
Supplement: Supplementary file 10 — Movie EV8 [file EMBJ-42-e114473-s009.zip › Movie_EV8/Movie_EV8.docx]

**Movie EV8. Behaviour of injected Alexa555-secondary antibody/AP1γ primary antibody conjugates in wild-type embryo preinjected with α-GFP or α-Dic antibodies.** Apical is to the top. Scale bar, 10 μm.
