# Supplementary material for: HEATR5B associates with dynein‐dynactin and promotes motility of AP1‐bound endosomal membranes
Source: EMBO J. 2023 Oct 24;42(23):e114473. doi: 10.15252/embj.2023114473 (PMC10690479; doi:10.15252/embj.2023114473)
Supplement: Supplementary file 11 — Movie EV9 [file EMBJ-42-e114473-s023.zip › Movie_EV9/Movie_EV9.docx]

**Movie EV9. Behaviour of injected Alexa488-labelled *bcd* RNA in control (*nos-cas9*) and *cas9 gRNA-Hr5^1+2^* blastoderm embryos.** Apical is to the top. Scale bar, 10 μm.
