# Supplementary material for: HEATR5B associates with dynein‐dynactin and promotes motility of AP1‐bound endosomal membranes
Source: EMBO J. 2023 Oct 24;42(23):e114473. doi: 10.15252/embj.2023114473 (PMC10690479; doi:10.15252/embj.2023114473)
Supplement: Supplementary file 12 — Movie EV10 [file EMBJ-42-e114473-s020.zip › Movie_EV10/Movie_EV10.docx]

**Movie EV10. Behaviour of injected Alexa555-secondary antibody/AP1γ primary antibody conjugates with respect to GFP-Golgin-245.** Apical is to the top. Squares show regions and time periods in Movies EV11 and EV12. Scale bar, 10 μm.
