# Supplementary material for: HEATR5B associates with dynein‐dynactin and promotes motility of AP1‐bound endosomal membranes
Source: EMBO J. 2023 Oct 24;42(23):e114473. doi: 10.15252/embj.2023114473 (PMC10690479; doi:10.15252/embj.2023114473)
Supplement: Supplementary file 13 — Movie EV11 [file EMBJ-42-e114473-s005.zip › Movie_EV11/Movie_EV11.docx]

**Movie EV11. Example of interaction of transported AP1γ particle with GFP-Golgin-245 in the upper region of the basal cytoplasm (crop of Movie EV10).** Yellow arrow shows AP1γ punctum that is transported apically, interacts with the Golgi, and is transported basally. Apical is to the top. Movie is looped. Scale bar, 2 μm.
