# Supplementary material for: HEATR5B associates with dynein‐dynactin and promotes motility of AP1‐bound endosomal membranes
Source: EMBO J. 2023 Oct 24;42(23):e114473. doi: 10.15252/embj.2023114473 (PMC10690479; doi:10.15252/embj.2023114473)
Supplement: Supplementary file 14 — Movie EV12 [file EMBJ-42-e114473-s004.zip › Movie_EV12/Movie_EV12.docx]

**Movie EV12. Additional example of co-transport of AP1γ and GFP-Golgin-245 in the upper region of the basal cytoplasm (crop of Movie EV10).** Yellow arrow shows an example of bidirectional co-transport of AP1γ in association with Golgin-245. Apical is to the top. Movie is looped. Scale bar, 2 μm.
