# Supplementary figures and images for: HEATR5B associates with dynein‐dynactin and promotes motility of AP1‐bound endosomal membranes
Source: EMBO J. 2023 Oct 24;42(23):e114473. doi: 10.15252/embj.2023114473 (PMC10690479; doi:10.15252/embj.2023114473)

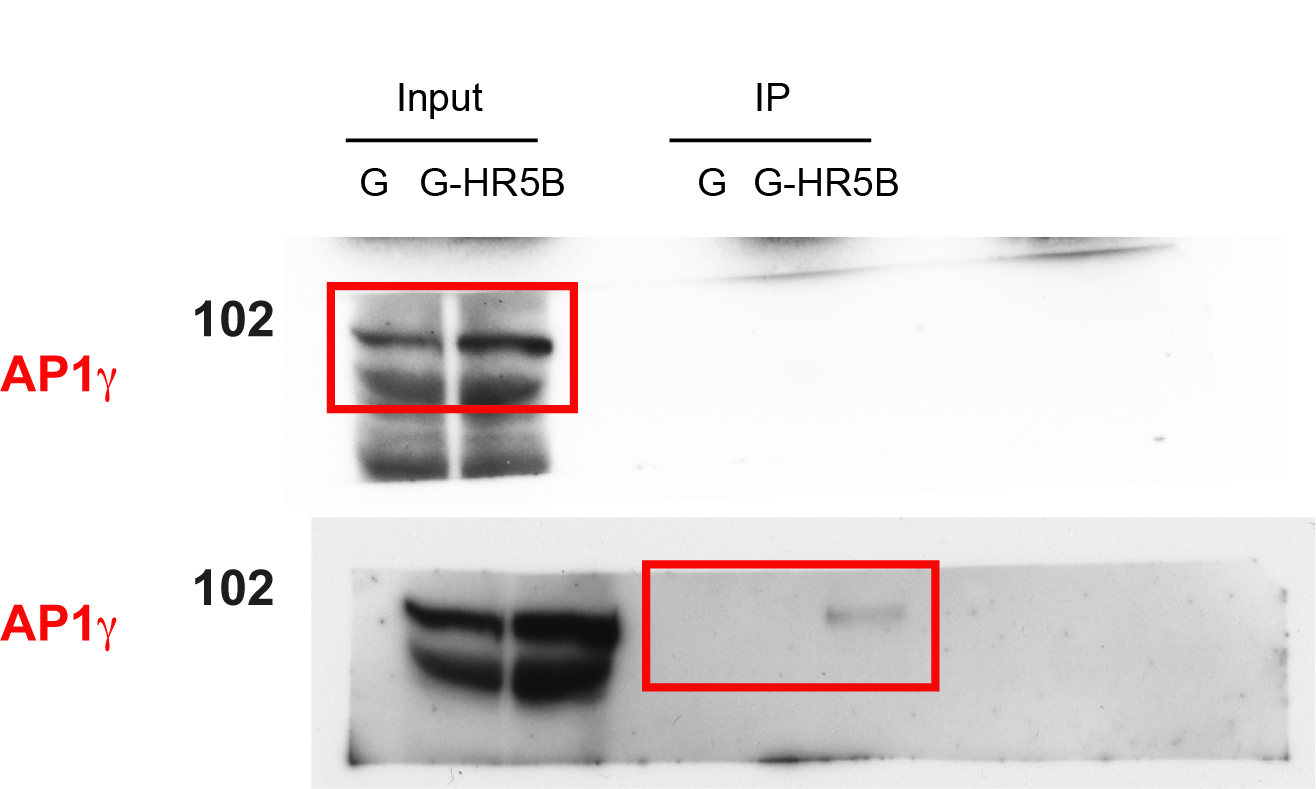

Supplement: Supplementary file 20 — Source Data for Figure 2 [file EMBJ-42-e114473-s018.zip › Figure_2/2A/western_AP1g.tif]

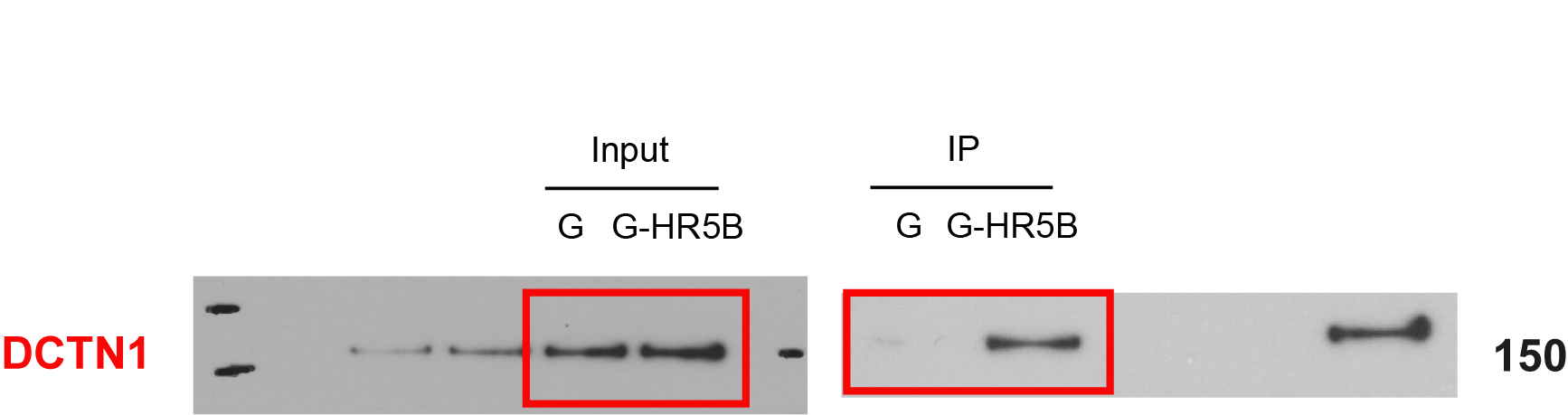

Supplement: Supplementary file 20 — Source Data for Figure 2 [file EMBJ-42-e114473-s018.zip › Figure_2/2A/western_DCTN1.tif]

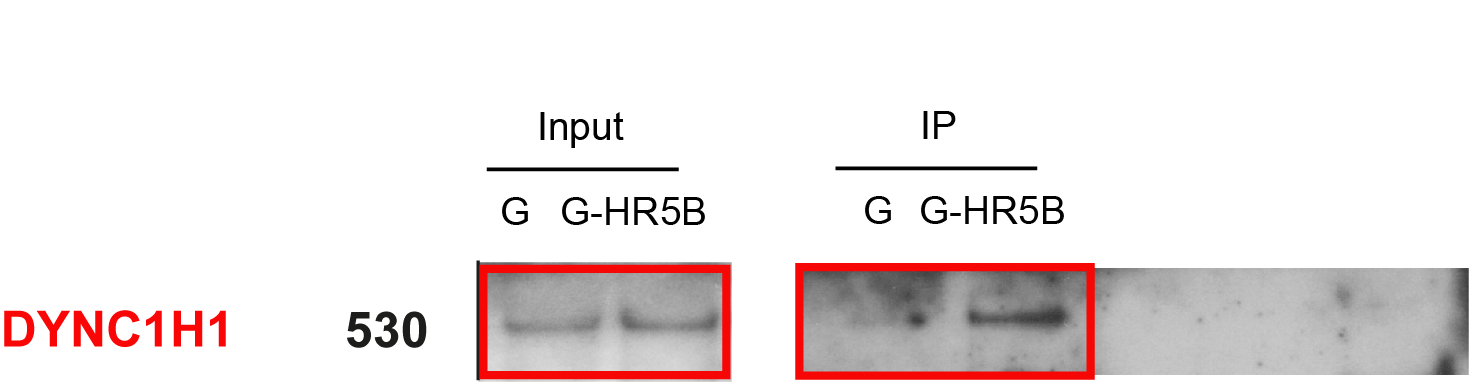

Supplement: Supplementary file 20 — Source Data for Figure 2 [file EMBJ-42-e114473-s018.zip › Figure_2/2A/western_DYNC1H1.tif]

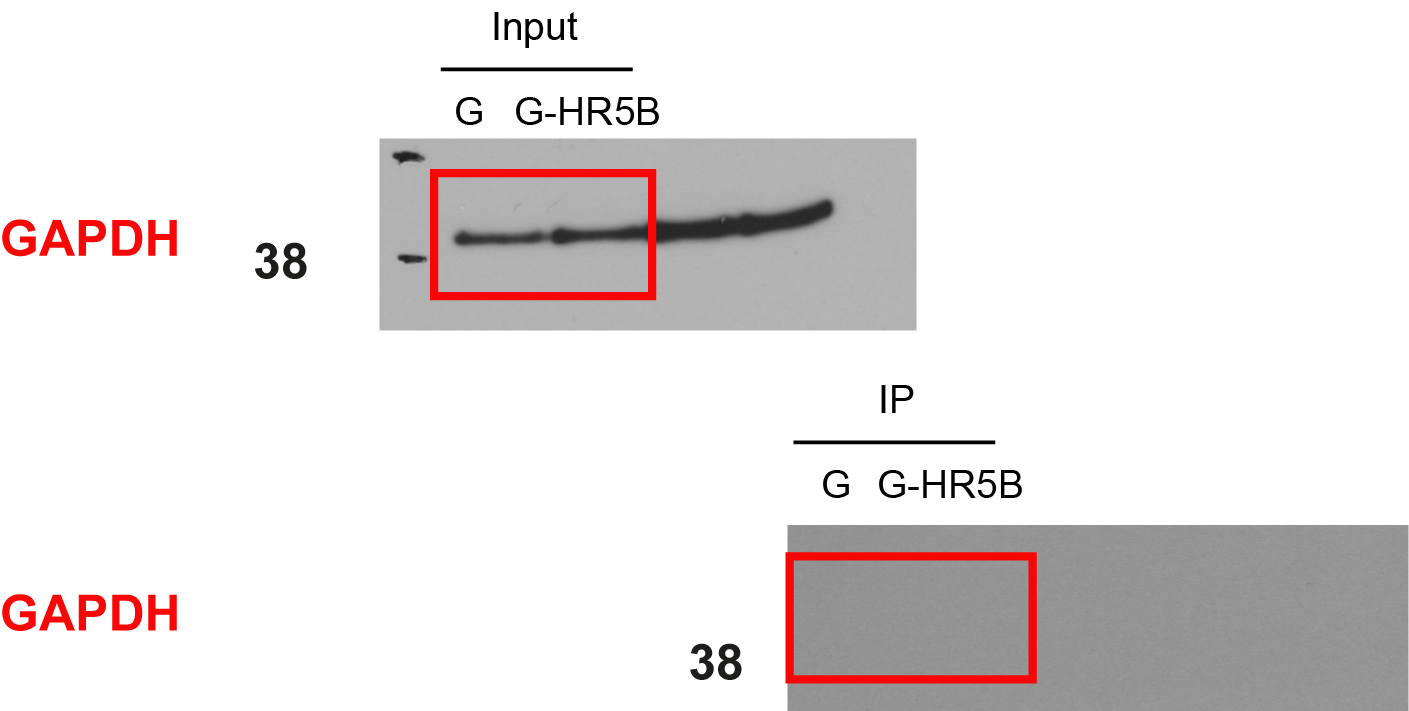

Supplement: Supplementary file 20 — Source Data for Figure 2 [file EMBJ-42-e114473-s018.zip › Figure_2/2A/western_GAPDH.tif]

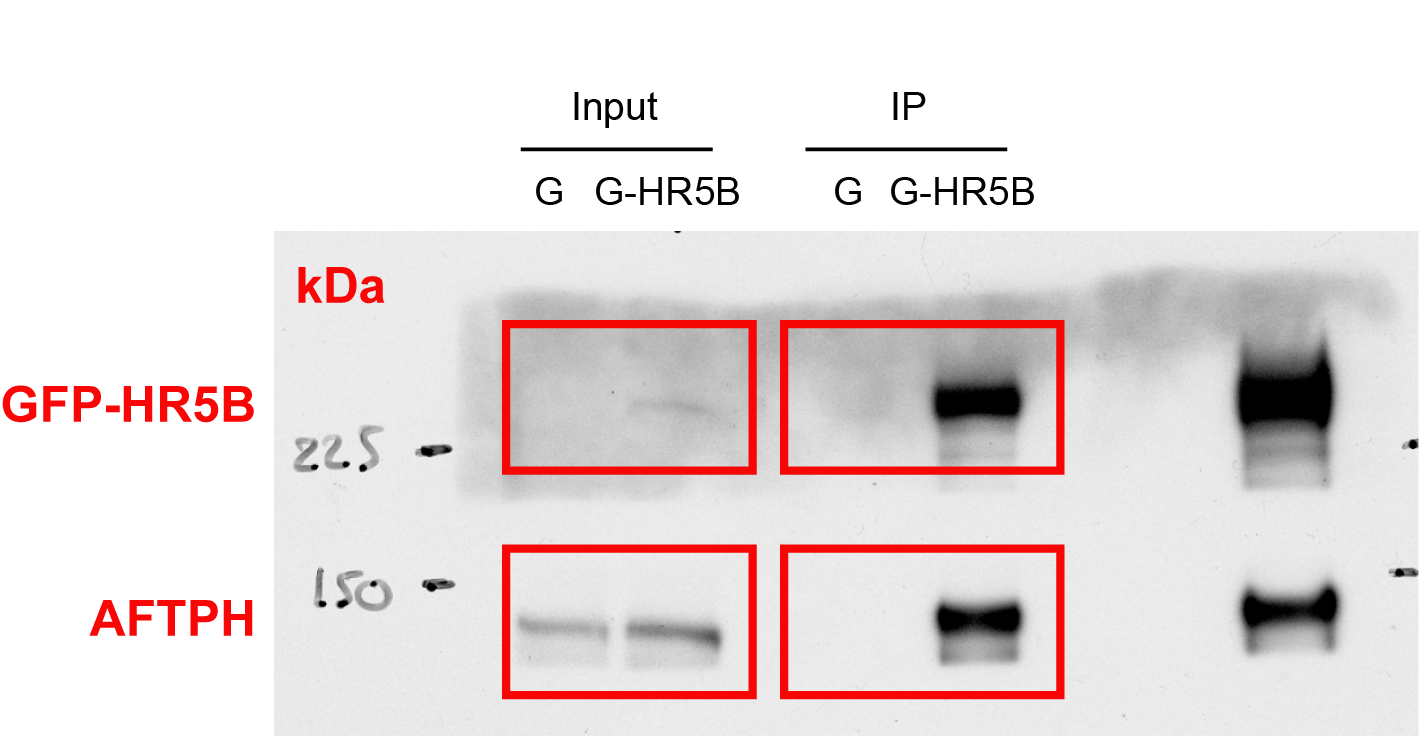

Supplement: Supplementary file 20 — Source Data for Figure 2 [file EMBJ-42-e114473-s018.zip › Figure_2/2A/western_GFP-HR5B_and_Aftiphilin.tif]

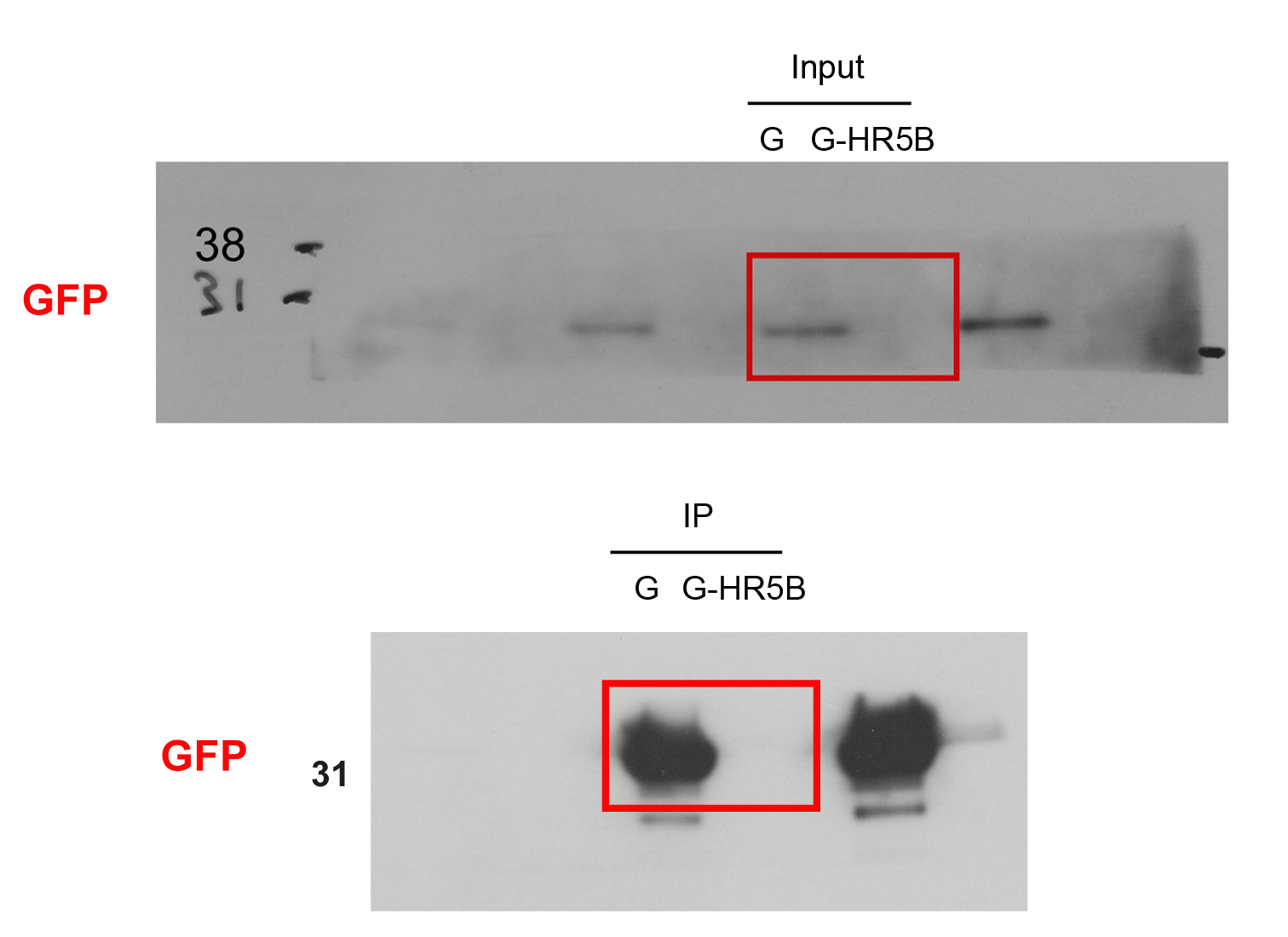

Supplement: Supplementary file 20 — Source Data for Figure 2 [file EMBJ-42-e114473-s018.zip › Figure_2/2A/western_GFP.tif]

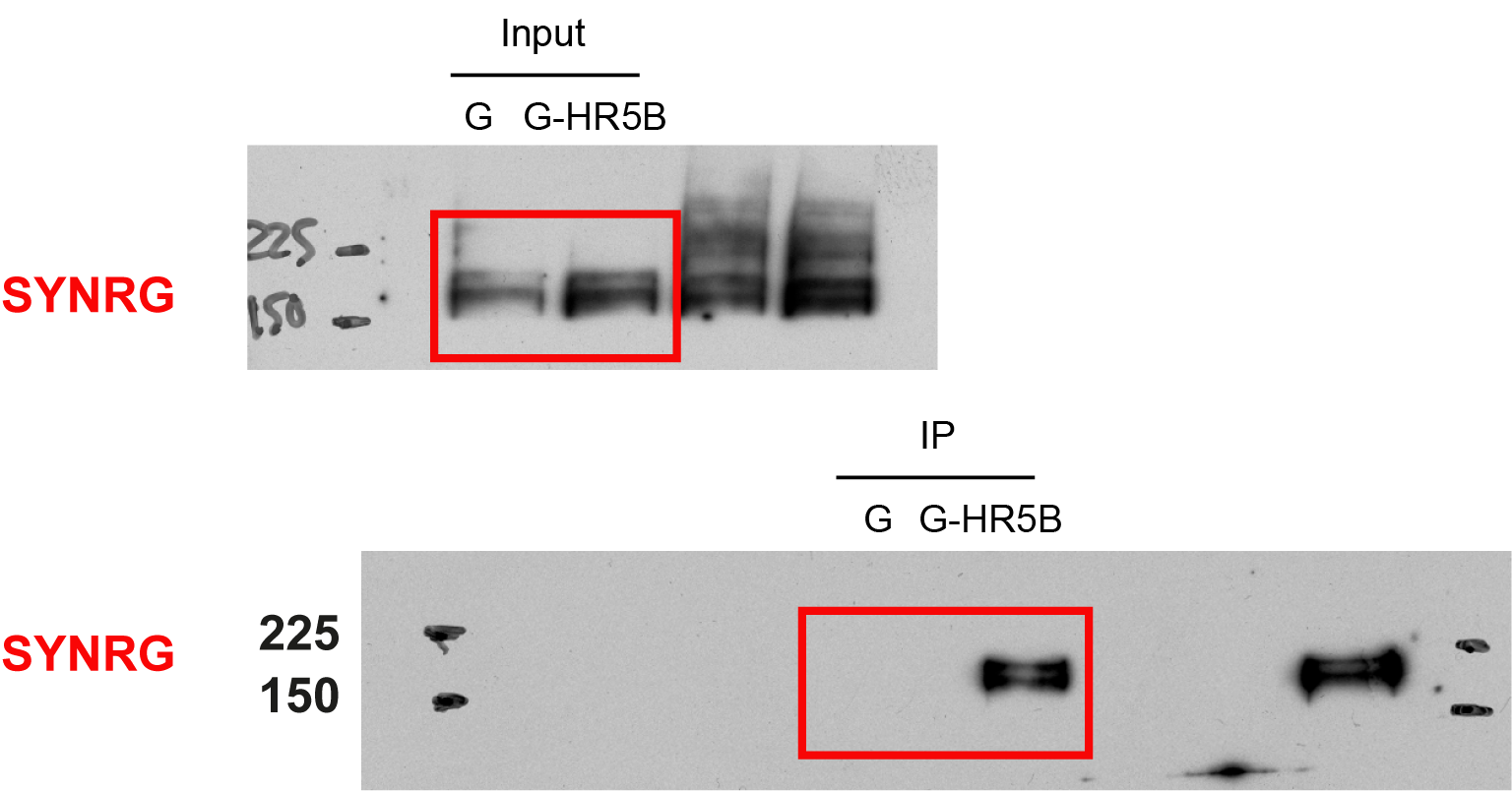

Supplement: Supplementary file 20 — Source Data for Figure 2 [file EMBJ-42-e114473-s018.zip › Figure_2/2A/western_SYNRG.tif]

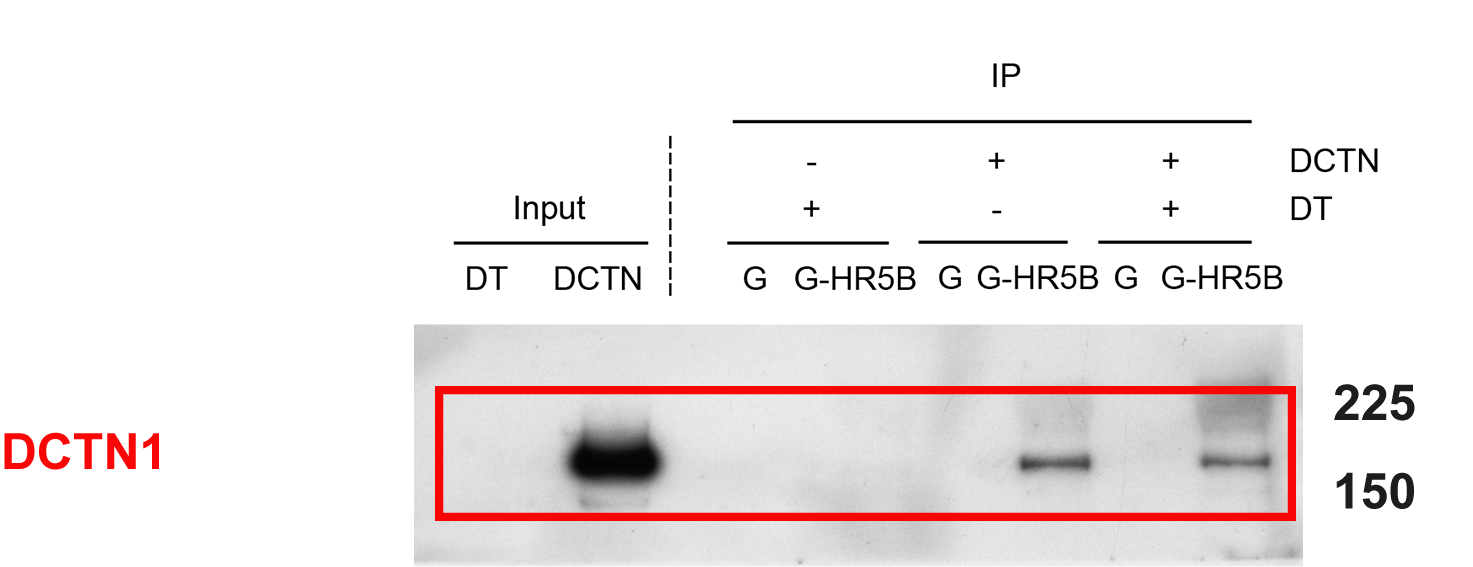

Supplement: Supplementary file 20 — Source Data for Figure 2 [file EMBJ-42-e114473-s018.zip › Figure_2/2B/western_DCTN1.tif]

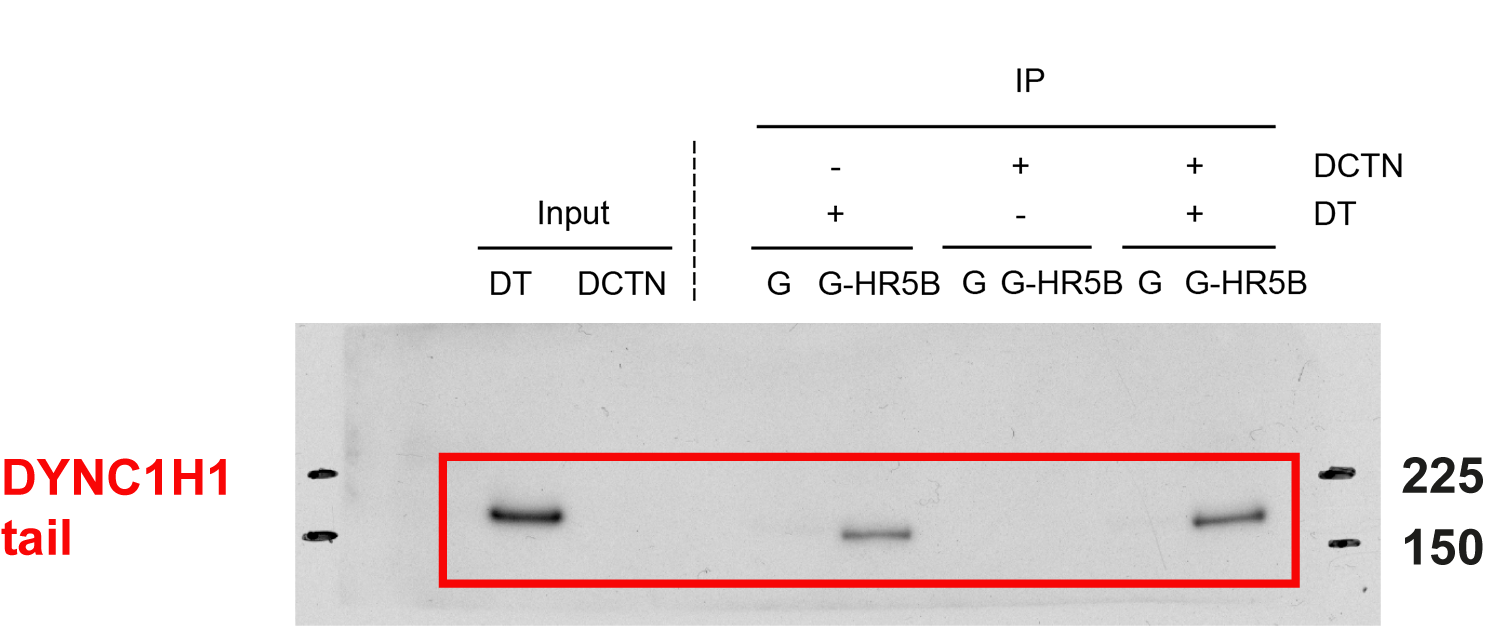

Supplement: Supplementary file 20 — Source Data for Figure 2 [file EMBJ-42-e114473-s018.zip › Figure_2/2B/western_DYNC1H1_tail.tif]

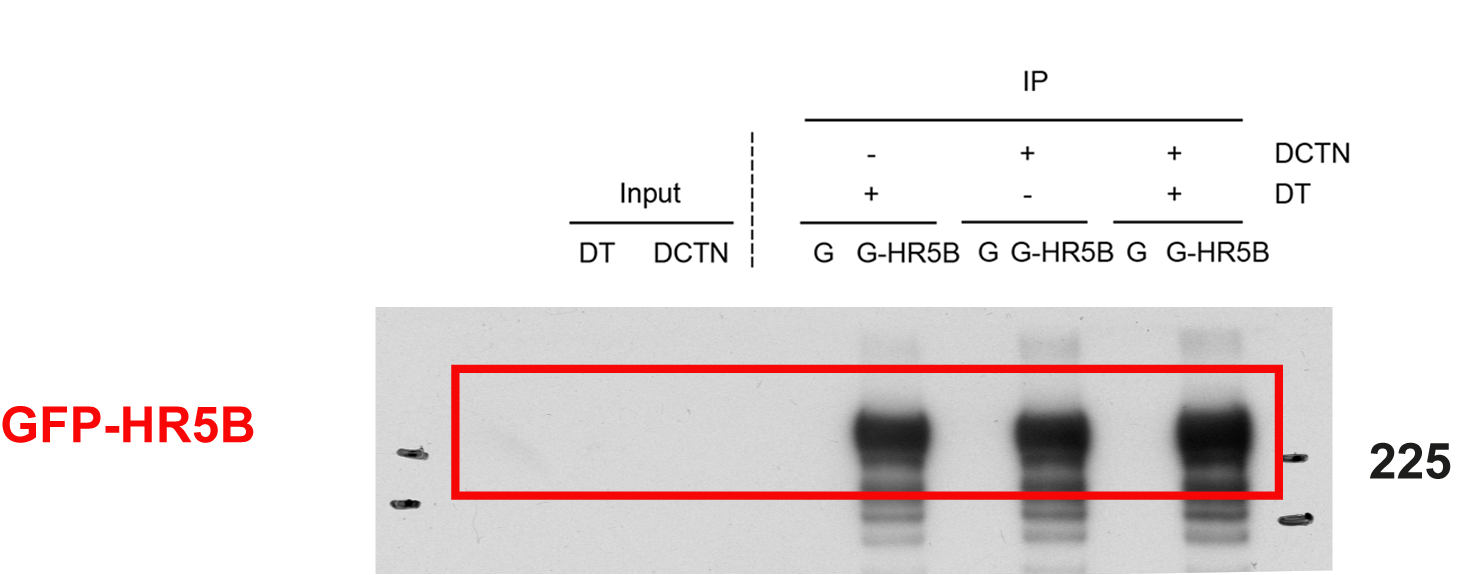

Supplement: Supplementary file 20 — Source Data for Figure 2 [file EMBJ-42-e114473-s018.zip › Figure_2/2B/western_GFP-HR5B.tif]

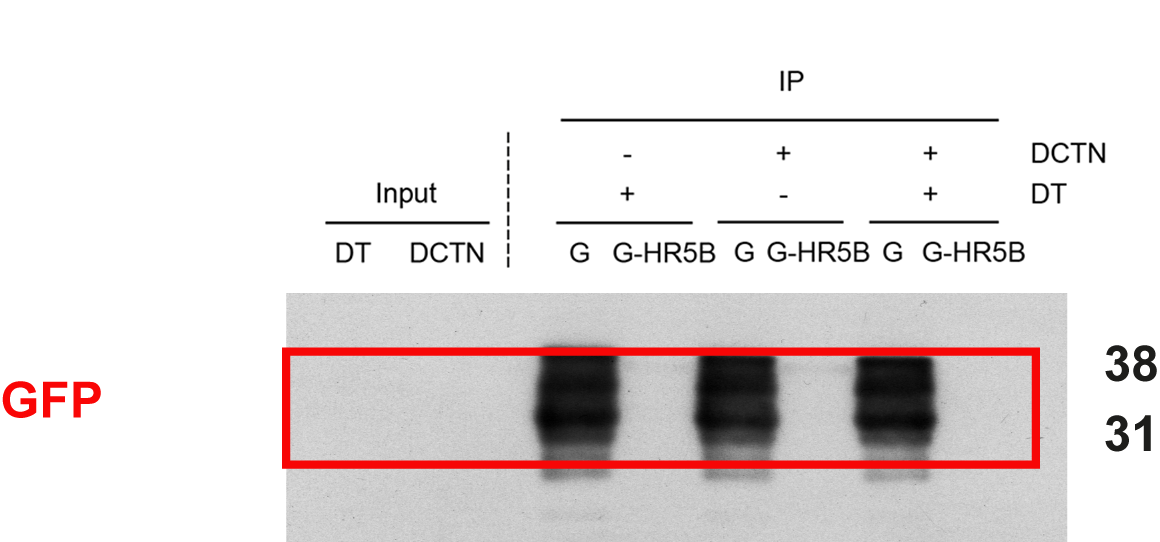

Supplement: Supplementary file 20 — Source Data for Figure 2 [file EMBJ-42-e114473-s018.zip › Figure_2/2B/western_GFP.tif]

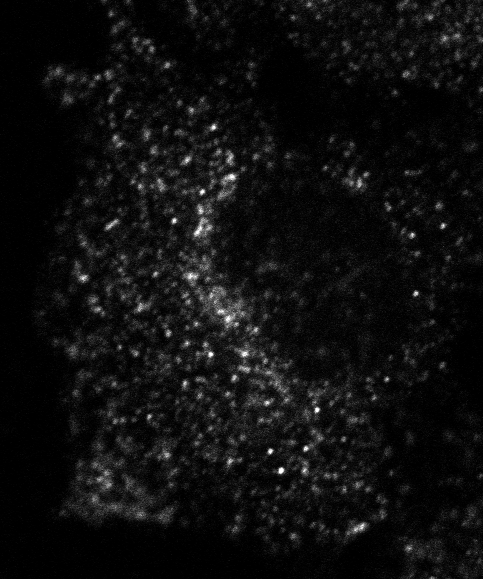

Supplement: Supplementary file 21 — Source Data for Figure 3 [file EMBJ-42-e114473-s024.zip › Figure_3/3A/AP1g.tif]

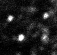

Supplement: Supplementary file 21 — Source Data for Figure 3 [file EMBJ-42-e114473-s024.zip › Figure_3/3A/Crop_AP1g.tif]

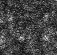

Supplement: Supplementary file 21 — Source Data for Figure 3 [file EMBJ-42-e114473-s024.zip › Figure_3/3A/Crop_eGFP-HR5B.tif]

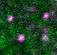

Supplement: Supplementary file 21 — Source Data for Figure 3 [file EMBJ-42-e114473-s024.zip › Figure_3/3A/Crop_Merge.tif]

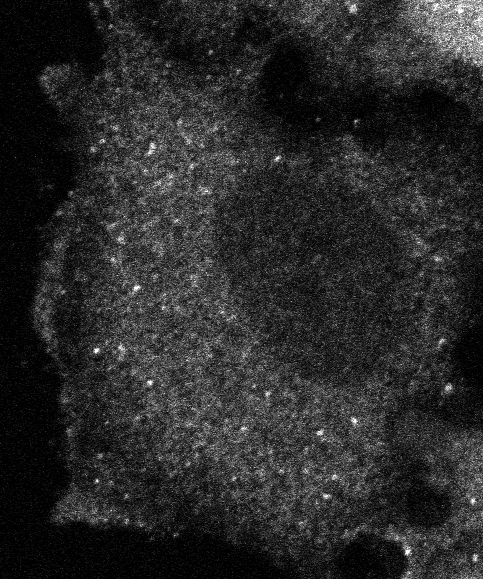

Supplement: Supplementary file 21 — Source Data for Figure 3 [file EMBJ-42-e114473-s024.zip › Figure_3/3A/GFP-HR5B.tif]

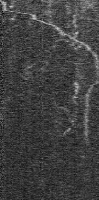

Supplement: Supplementary file 21 — Source Data for Figure 3 [file EMBJ-42-e114473-s024.zip › Figure_3/3D/AP1-sigma_RFP.tif]

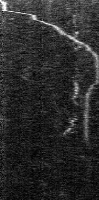

Supplement: Supplementary file 21 — Source Data for Figure 3 [file EMBJ-42-e114473-s024.zip › Figure_3/3D/GFP-HR5B.tif]

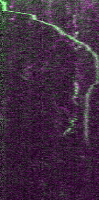

Supplement: Supplementary file 21 — Source Data for Figure 3 [file EMBJ-42-e114473-s024.zip › Figure_3/3D/Merge.tif]

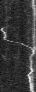

Supplement: Supplementary file 21 — Source Data for Figure 3 [file EMBJ-42-e114473-s024.zip › Figure_3/3F/DsRed-RAB11A.tif]

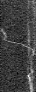

Supplement: Supplementary file 21 — Source Data for Figure 3 [file EMBJ-42-e114473-s024.zip › Figure_3/3F/GFP-HR5B.tif]

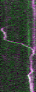

Supplement: Supplementary file 21 — Source Data for Figure 3 [file EMBJ-42-e114473-s024.zip › Figure_3/3F/Merge.tif]

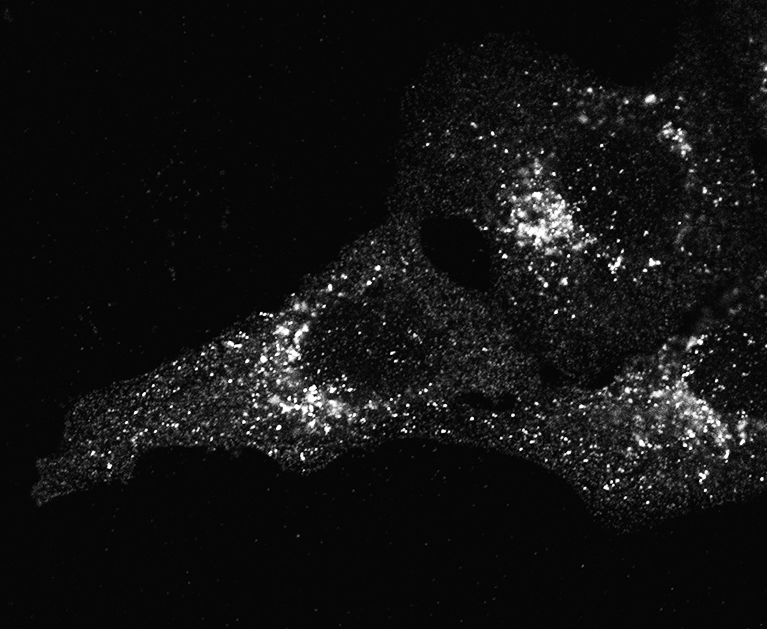

Supplement: Supplementary file 21 — Source Data for Figure 3 [file EMBJ-42-e114473-s024.zip › Figure_3/3G/Control_AP1_gamma.tif]

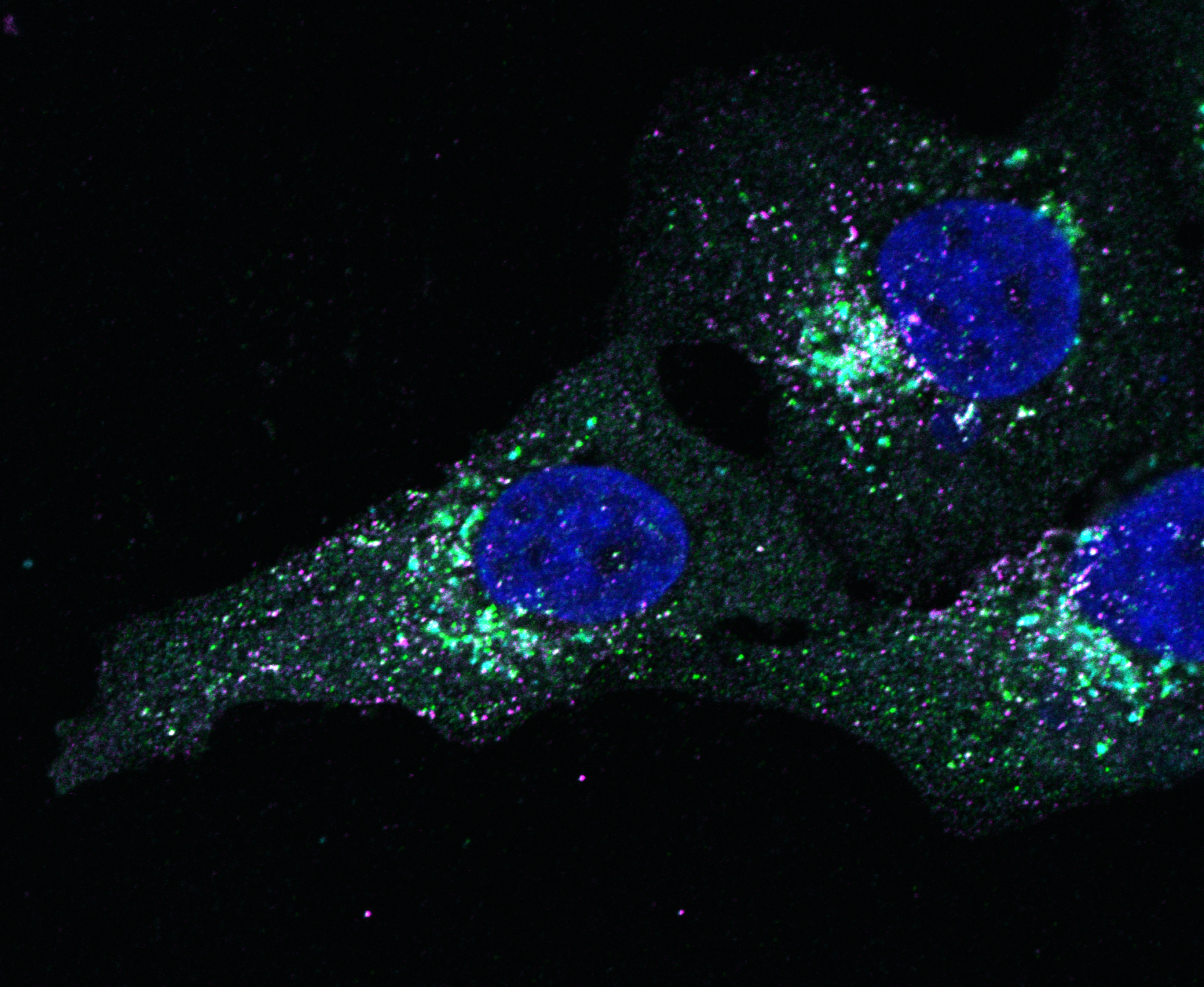

Supplement: Supplementary file 21 — Source Data for Figure 3 [file EMBJ-42-e114473-s024.zip › Figure_3/3G/Control_merge.tif]

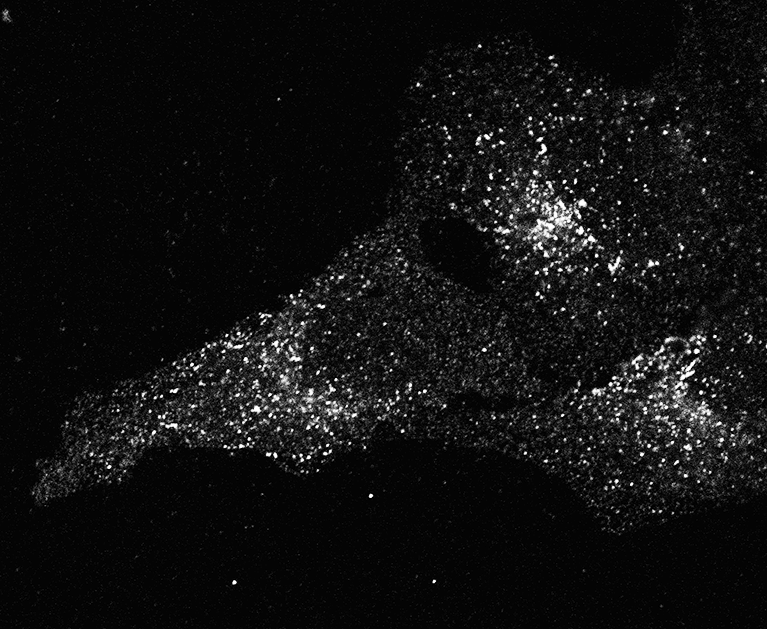

Supplement: Supplementary file 21 — Source Data for Figure 3 [file EMBJ-42-e114473-s024.zip › Figure_3/3G/Control_RAB11A.tif]

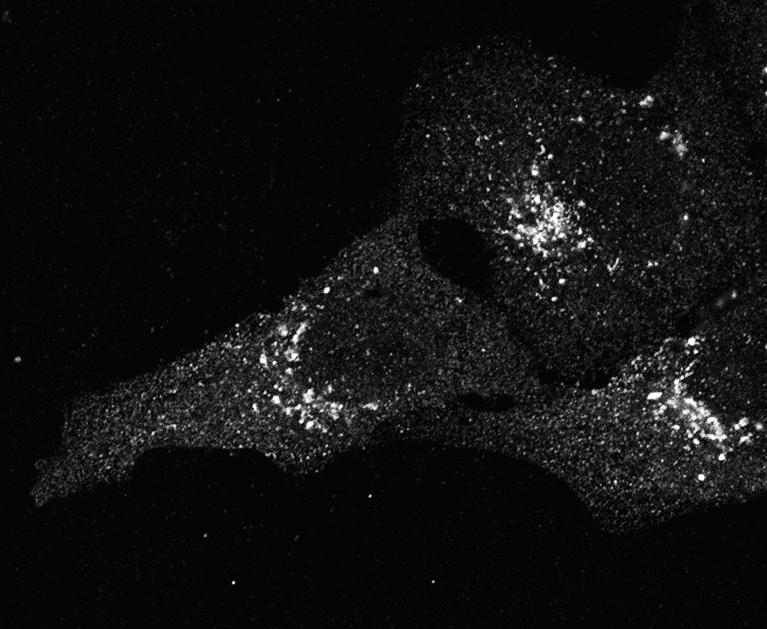

Supplement: Supplementary file 21 — Source Data for Figure 3 [file EMBJ-42-e114473-s024.zip › Figure_3/3G/Control_TGN46.tif]

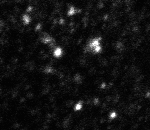

Supplement: Supplementary file 21 — Source Data for Figure 3 [file EMBJ-42-e114473-s024.zip › Figure_3/3G/Crop_control_AP1_gamma.tif]

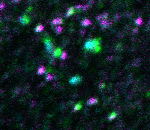

Supplement: Supplementary file 21 — Source Data for Figure 3 [file EMBJ-42-e114473-s024.zip › Figure_3/3G/Crop_control_merge.tif]

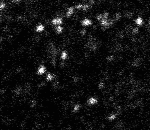

Supplement: Supplementary file 21 — Source Data for Figure 3 [file EMBJ-42-e114473-s024.zip › Figure_3/3G/Crop_control_RAB11A.tif]

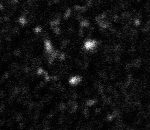

Supplement: Supplementary file 21 — Source Data for Figure 3 [file EMBJ-42-e114473-s024.zip › Figure_3/3G/Crop_control_TGN46.tif]

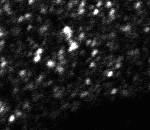

Supplement: Supplementary file 21 — Source Data for Figure 3 [file EMBJ-42-e114473-s024.zip › Figure_3/3G/Crop_siRNA_DYNC1H1_AP1_gamma.tif]

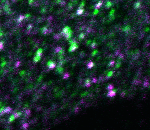

Supplement: Supplementary file 21 — Source Data for Figure 3 [file EMBJ-42-e114473-s024.zip › Figure_3/3G/Crop_siRNA_DYNC1H1_merge.tif]

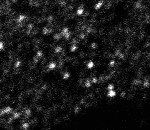

Supplement: Supplementary file 21 — Source Data for Figure 3 [file EMBJ-42-e114473-s024.zip › Figure_3/3G/Crop_siRNA_DYNC1H1_RAB11A.tif]

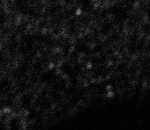

Supplement: Supplementary file 21 — Source Data for Figure 3 [file EMBJ-42-e114473-s024.zip › Figure_3/3G/Crop_siRNA_DYNC1H1_TGN.tif]

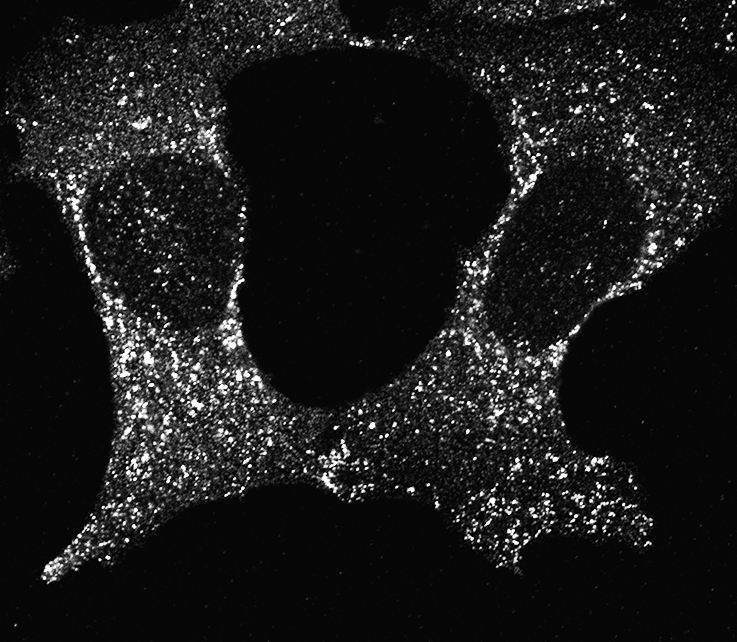

Supplement: Supplementary file 21 — Source Data for Figure 3 [file EMBJ-42-e114473-s024.zip › Figure_3/3G/siRNA_DYNC1H1_AP1_gamma.tif]

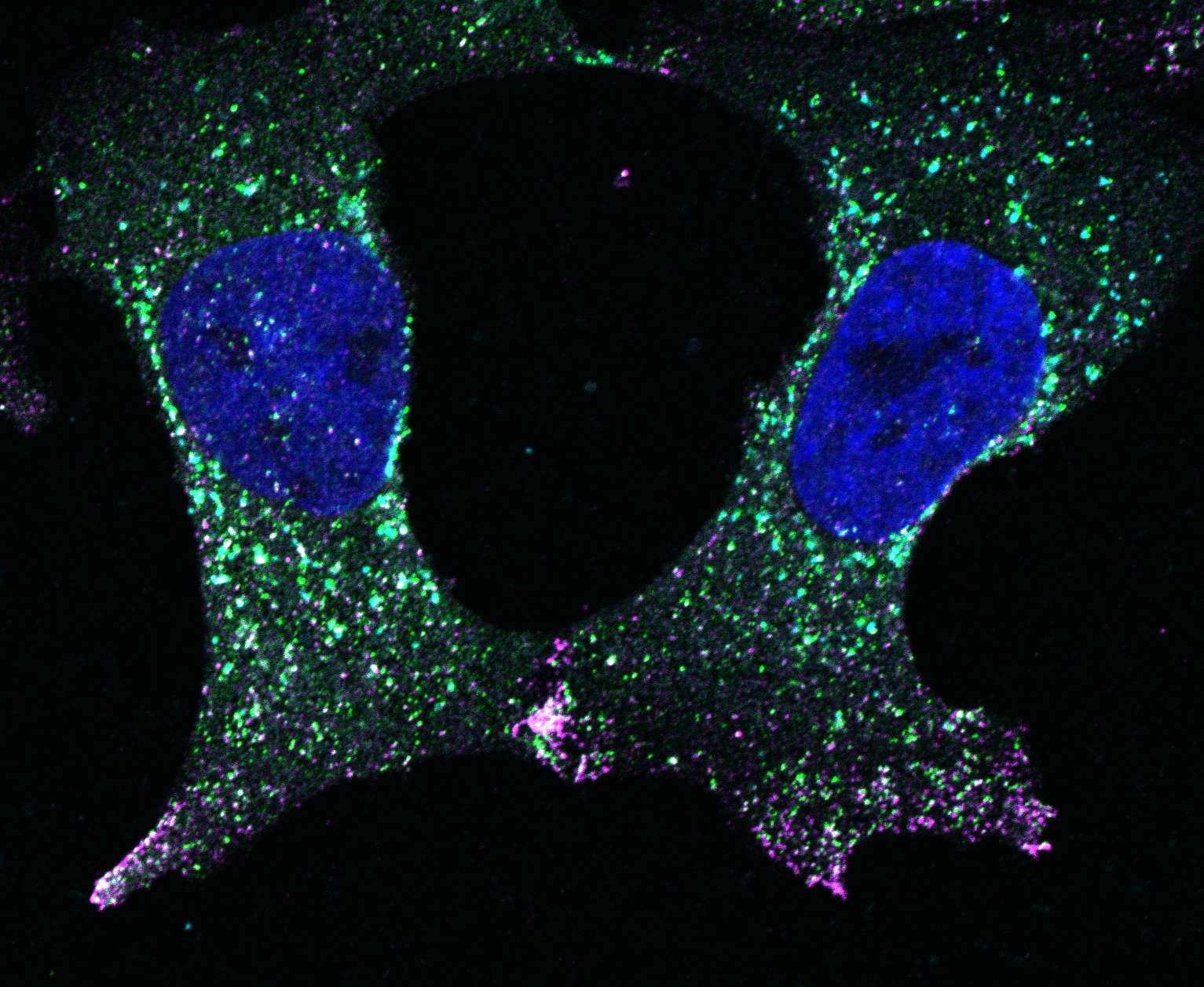

Supplement: Supplementary file 21 — Source Data for Figure 3 [file EMBJ-42-e114473-s024.zip › Figure_3/3G/siRNA_DYNC1H1_merge.tif]

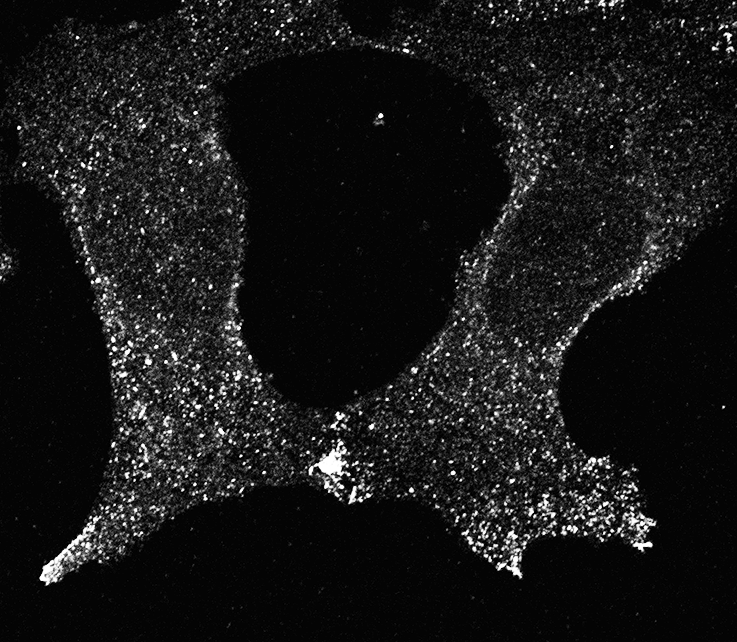

Supplement: Supplementary file 21 — Source Data for Figure 3 [file EMBJ-42-e114473-s024.zip › Figure_3/3G/siRNA_DYNC1H1_RAB11A.tif]

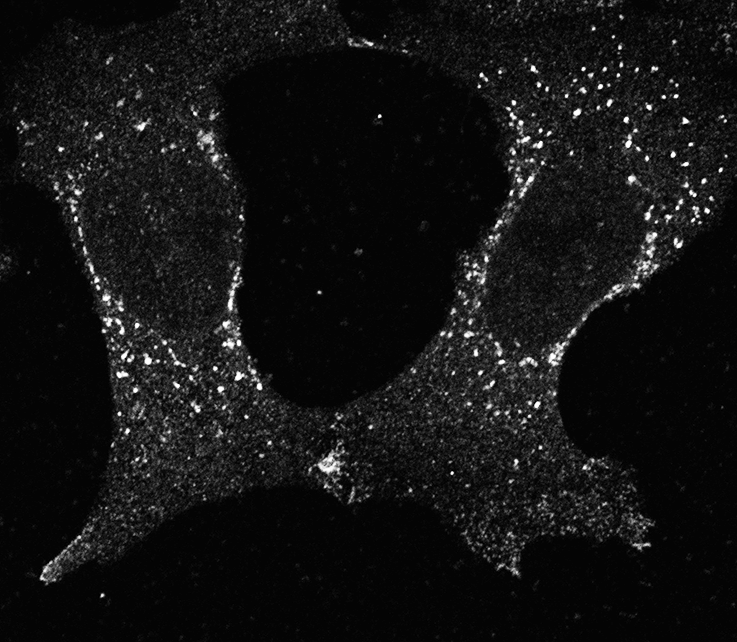

Supplement: Supplementary file 21 — Source Data for Figure 3 [file EMBJ-42-e114473-s024.zip › Figure_3/3G/siRNA_DYNC1H1_TGN46.tif]

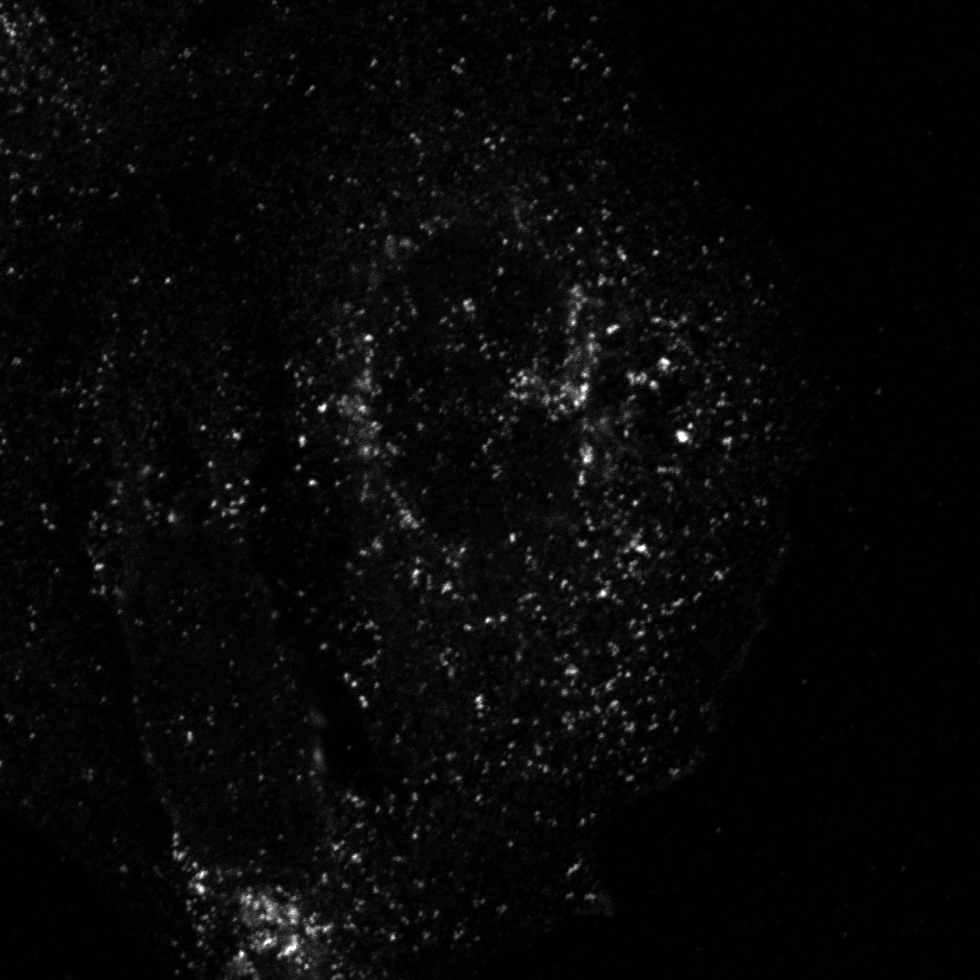

Supplement: Supplementary file 22 — Source Data for Figure 4 [file EMBJ-42-e114473-s001.zip › Figure_4/4A/AP1g_control.tif]

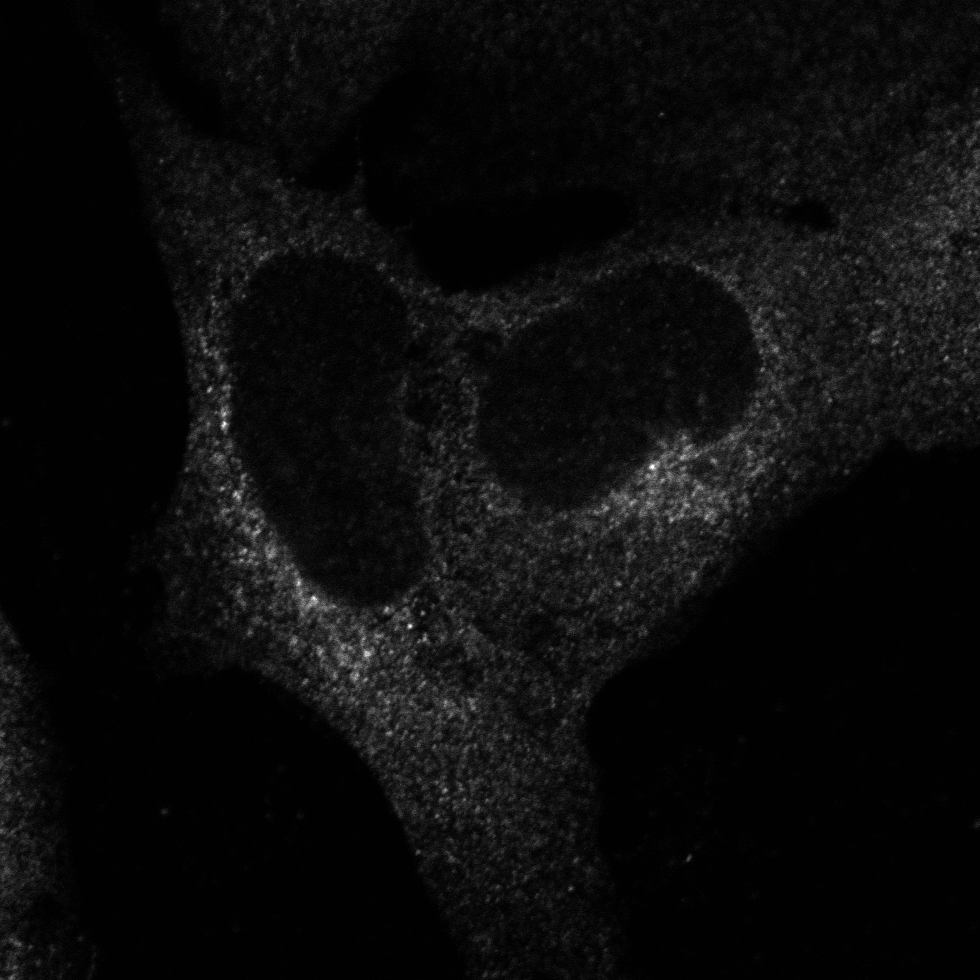

Supplement: Supplementary file 22 — Source Data for Figure 4 [file EMBJ-42-e114473-s001.zip › Figure_4/4A/AP1g_KO.tif]

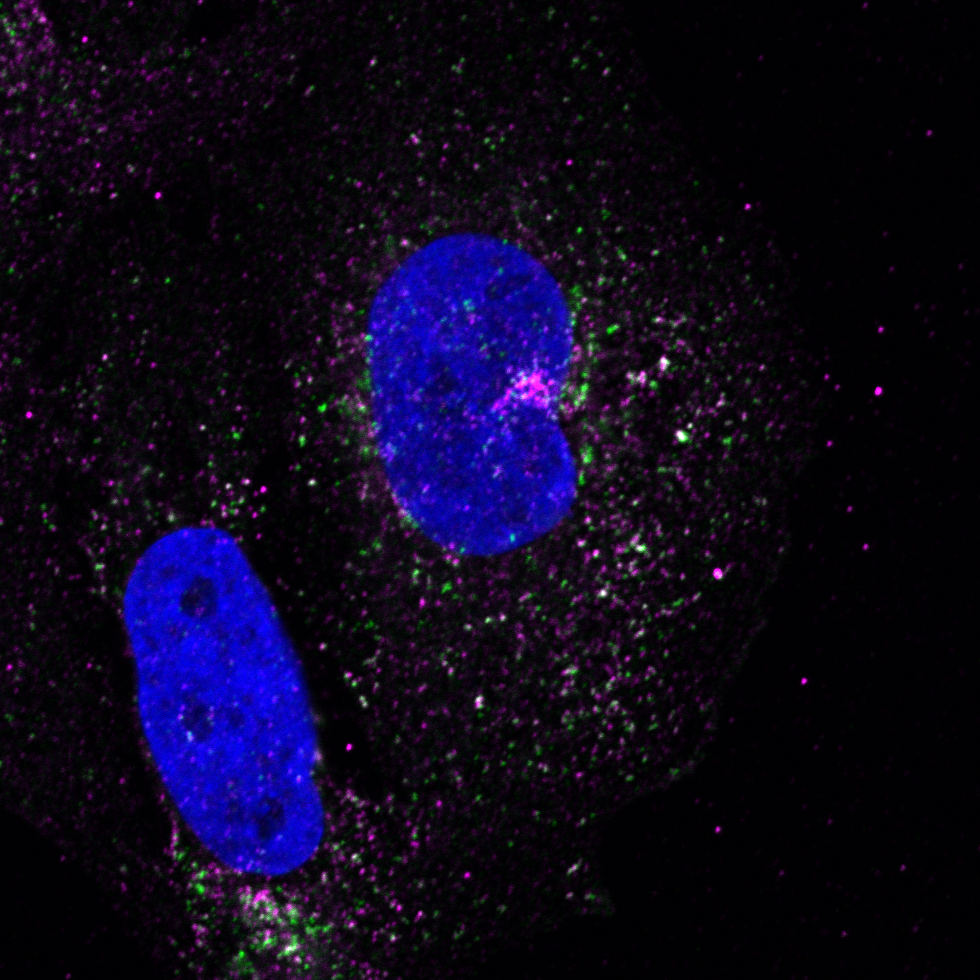

Supplement: Supplementary file 22 — Source Data for Figure 4 [file EMBJ-42-e114473-s001.zip › Figure_4/4A/composite_AP1g_RAB11A_DAPI_control.tif]

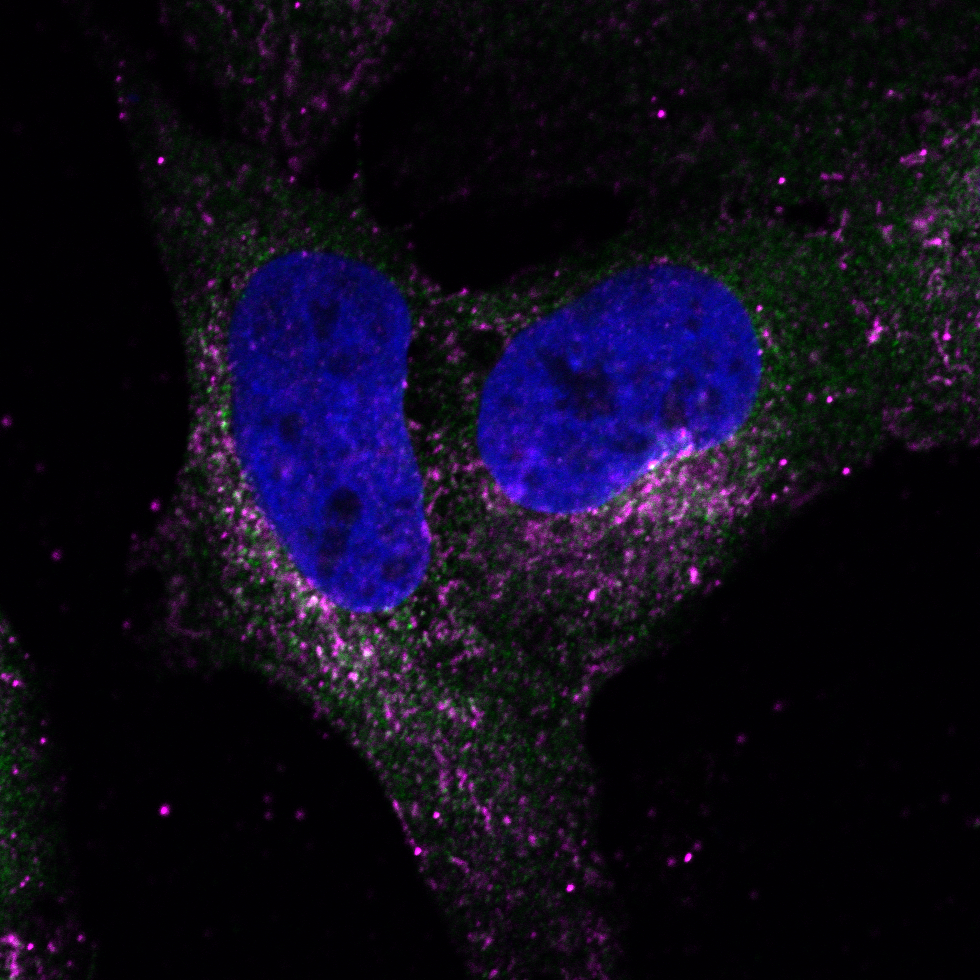

Supplement: Supplementary file 22 — Source Data for Figure 4 [file EMBJ-42-e114473-s001.zip › Figure_4/4A/composite_AP1g_RAB11A_DAPI_KO.tif]

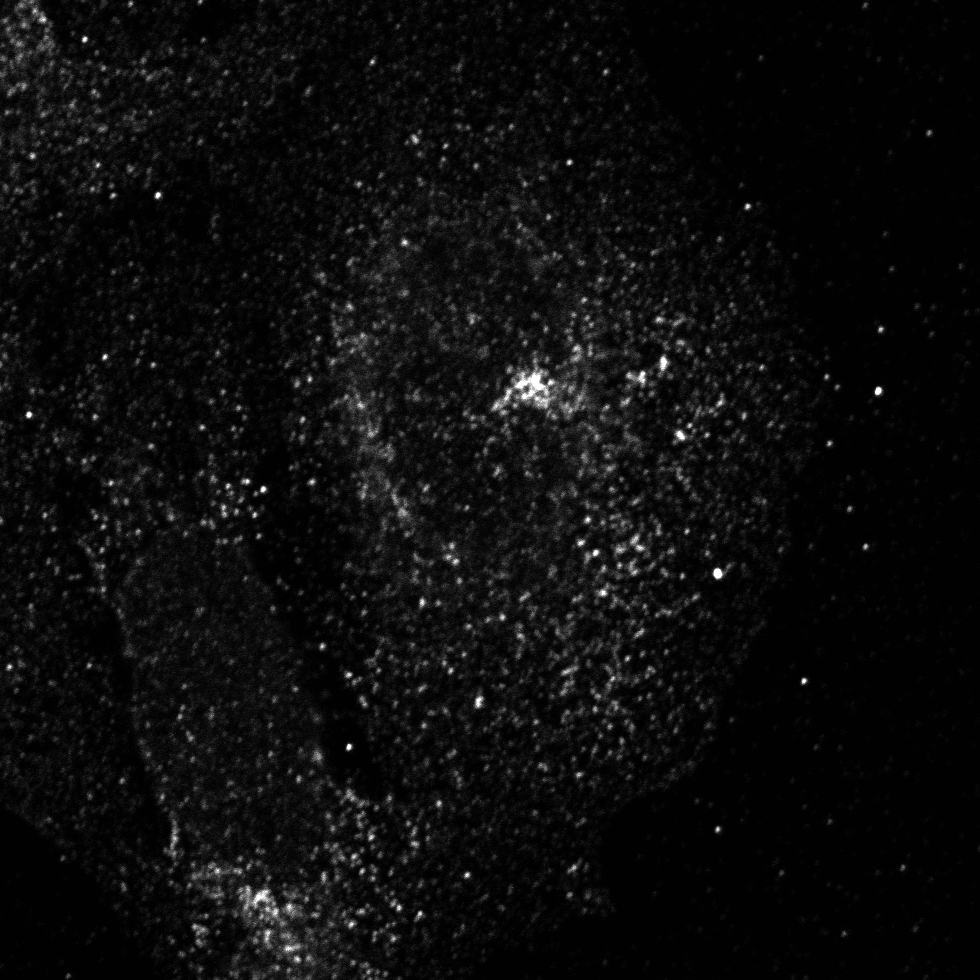

Supplement: Supplementary file 22 — Source Data for Figure 4 [file EMBJ-42-e114473-s001.zip › Figure_4/4A/RAB11A_control.tif]

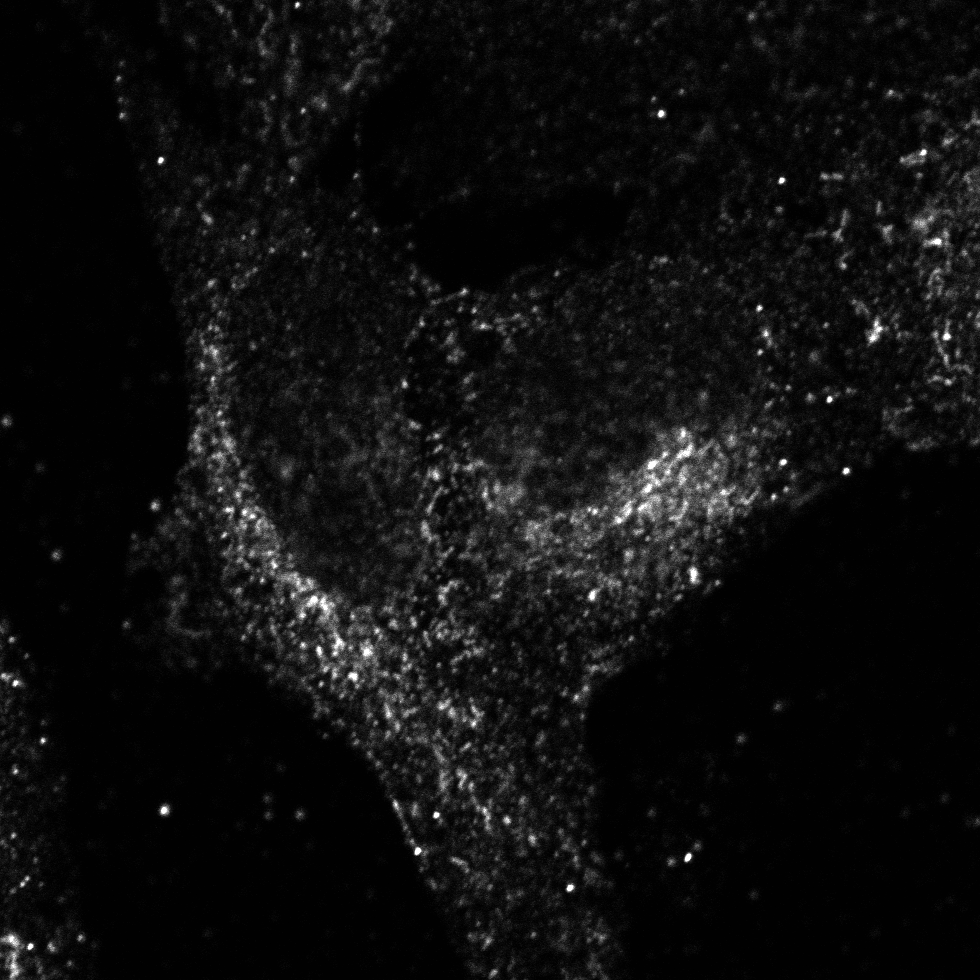

Supplement: Supplementary file 22 — Source Data for Figure 4 [file EMBJ-42-e114473-s001.zip › Figure_4/4A/RAB11A_KO.tif]

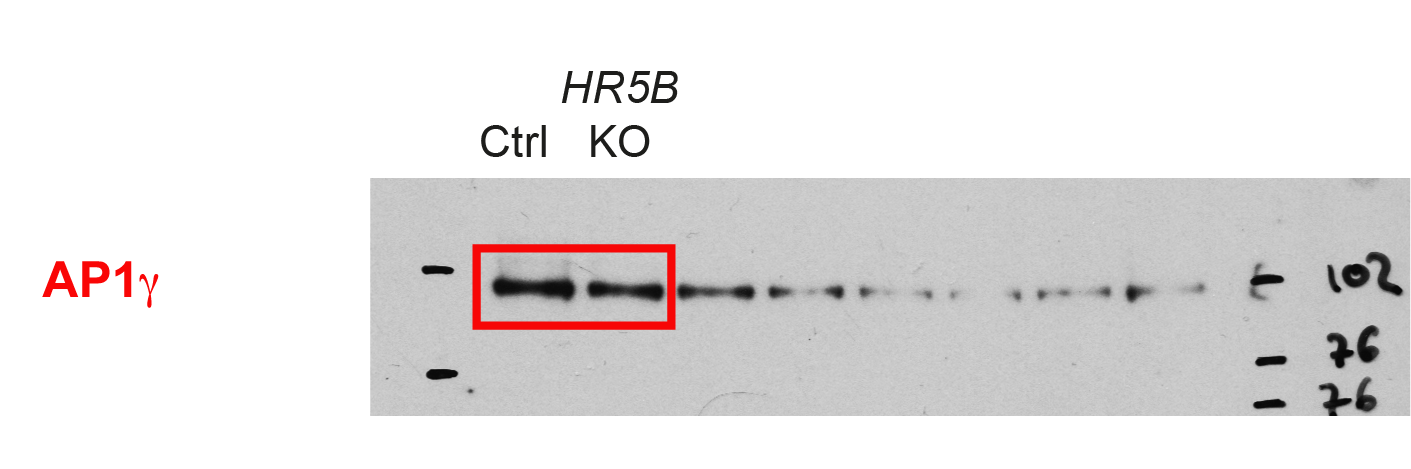

Supplement: Supplementary file 22 — Source Data for Figure 4 [file EMBJ-42-e114473-s001.zip › Figure_4/4D/western_AP1g.tif]

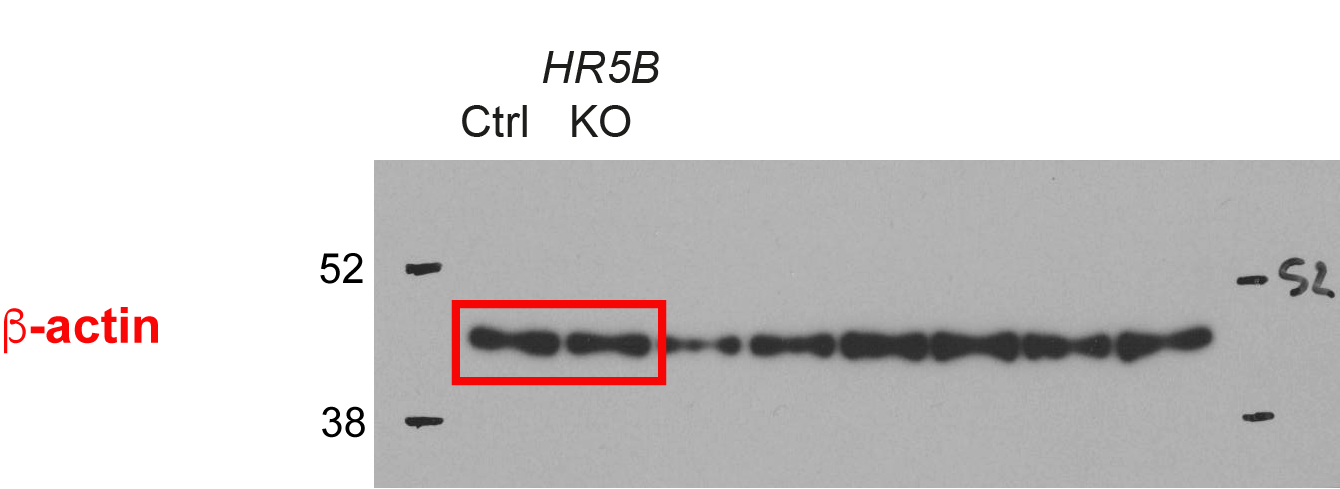

Supplement: Supplementary file 22 — Source Data for Figure 4 [file EMBJ-42-e114473-s001.zip › Figure_4/4D/western_b-actin.tif]

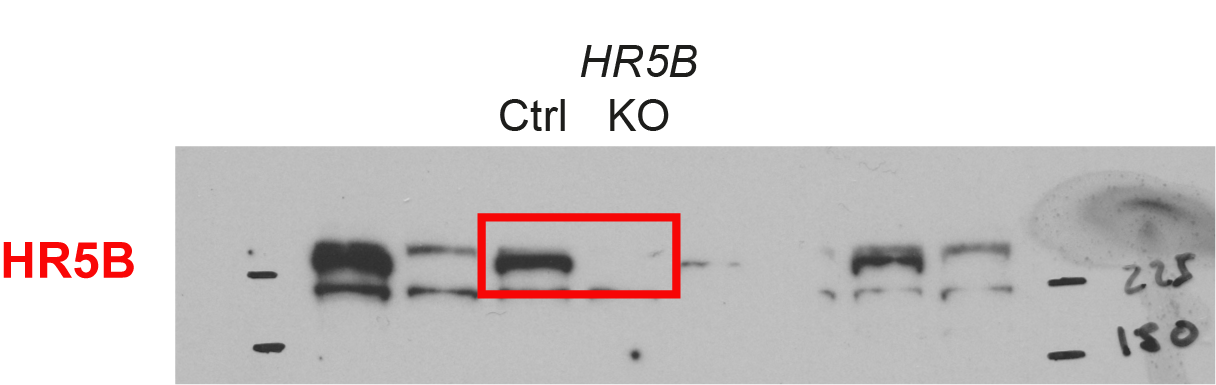

Supplement: Supplementary file 22 — Source Data for Figure 4 [file EMBJ-42-e114473-s001.zip › Figure_4/4D/western_HR5B.tif]

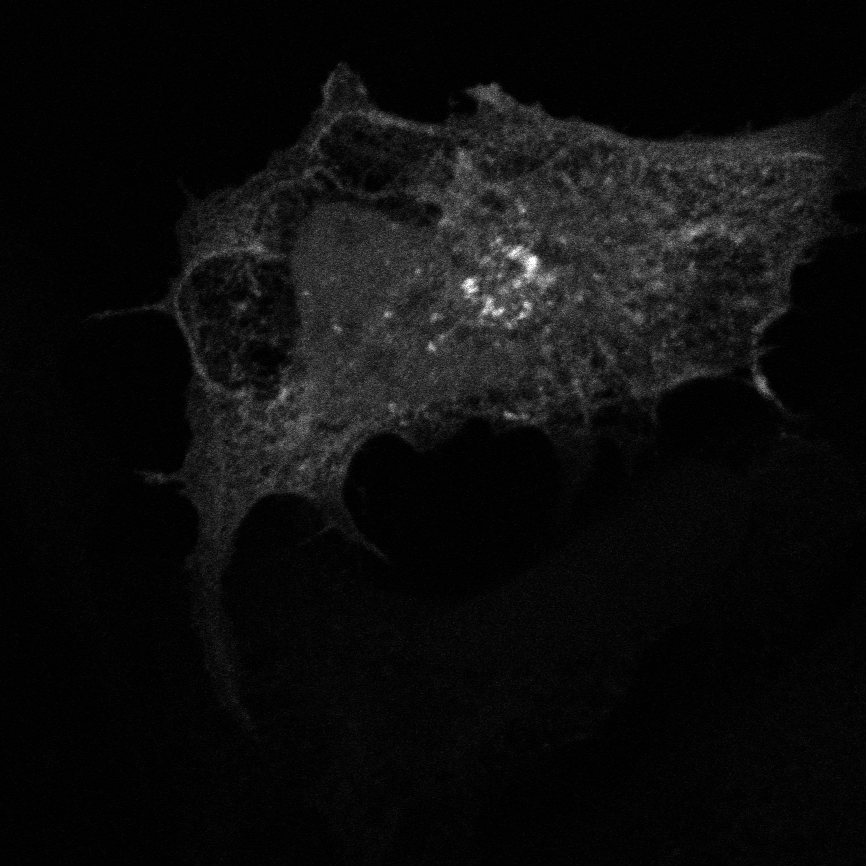

Supplement: Supplementary file 22 — Source Data for Figure 4 [file EMBJ-42-e114473-s001.zip › Figure_4/4G/GFP-HR5B.tif]

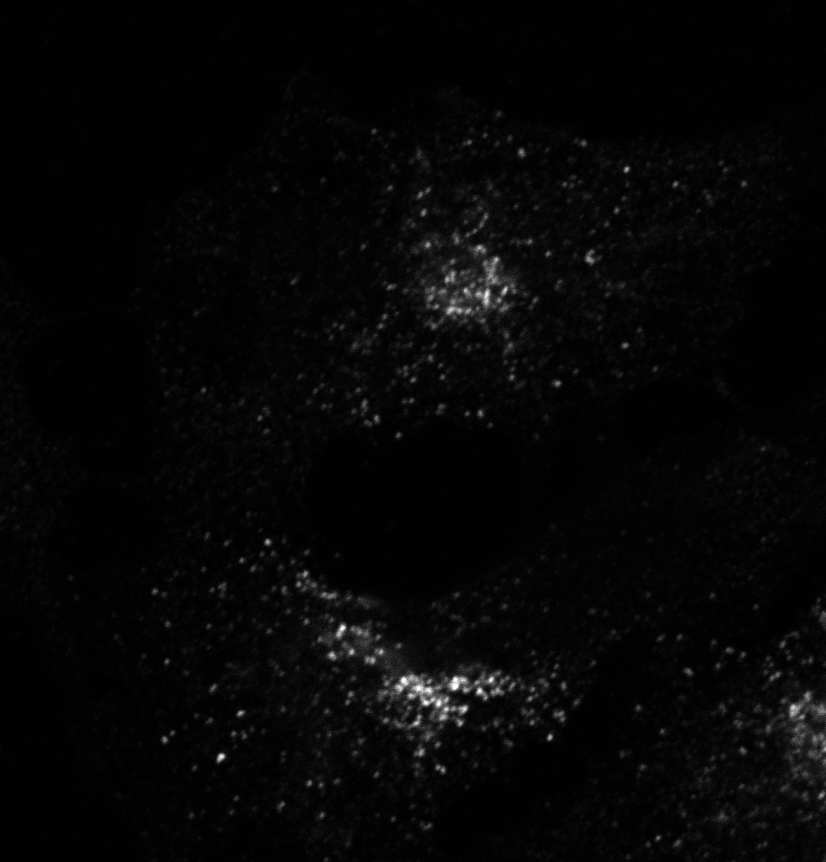

Supplement: Supplementary file 22 — Source Data for Figure 4 [file EMBJ-42-e114473-s001.zip › Figure_4/4G/GFP-HR5B_AP1_gamma.tif]

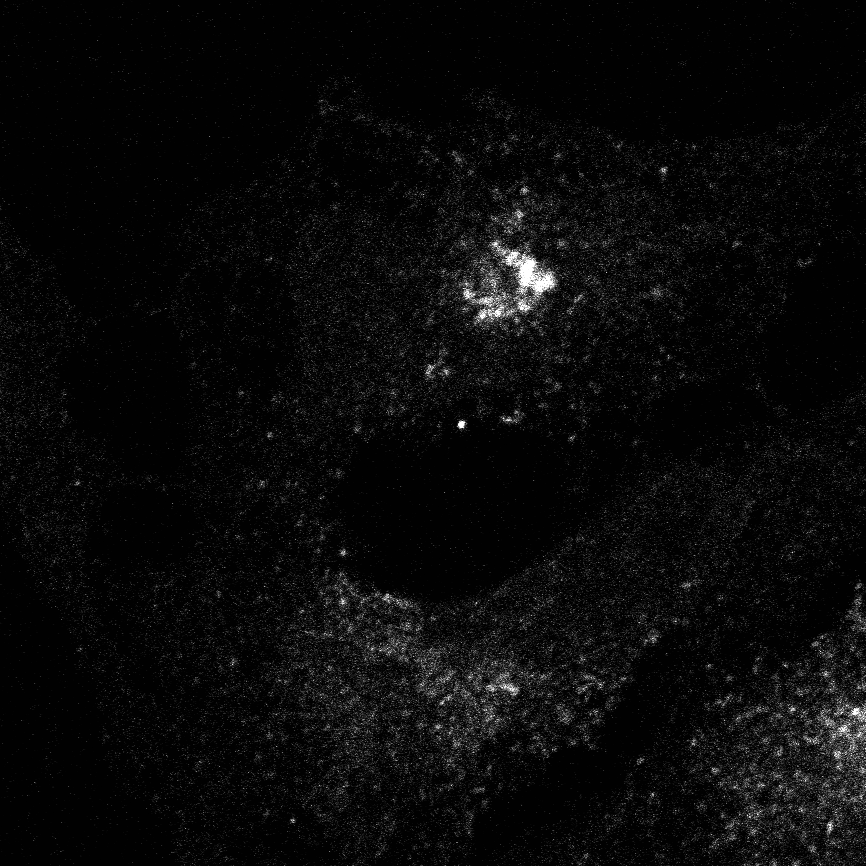

Supplement: Supplementary file 22 — Source Data for Figure 4 [file EMBJ-42-e114473-s001.zip › Figure_4/4G/GFP-HR5B_Rab11.tif]

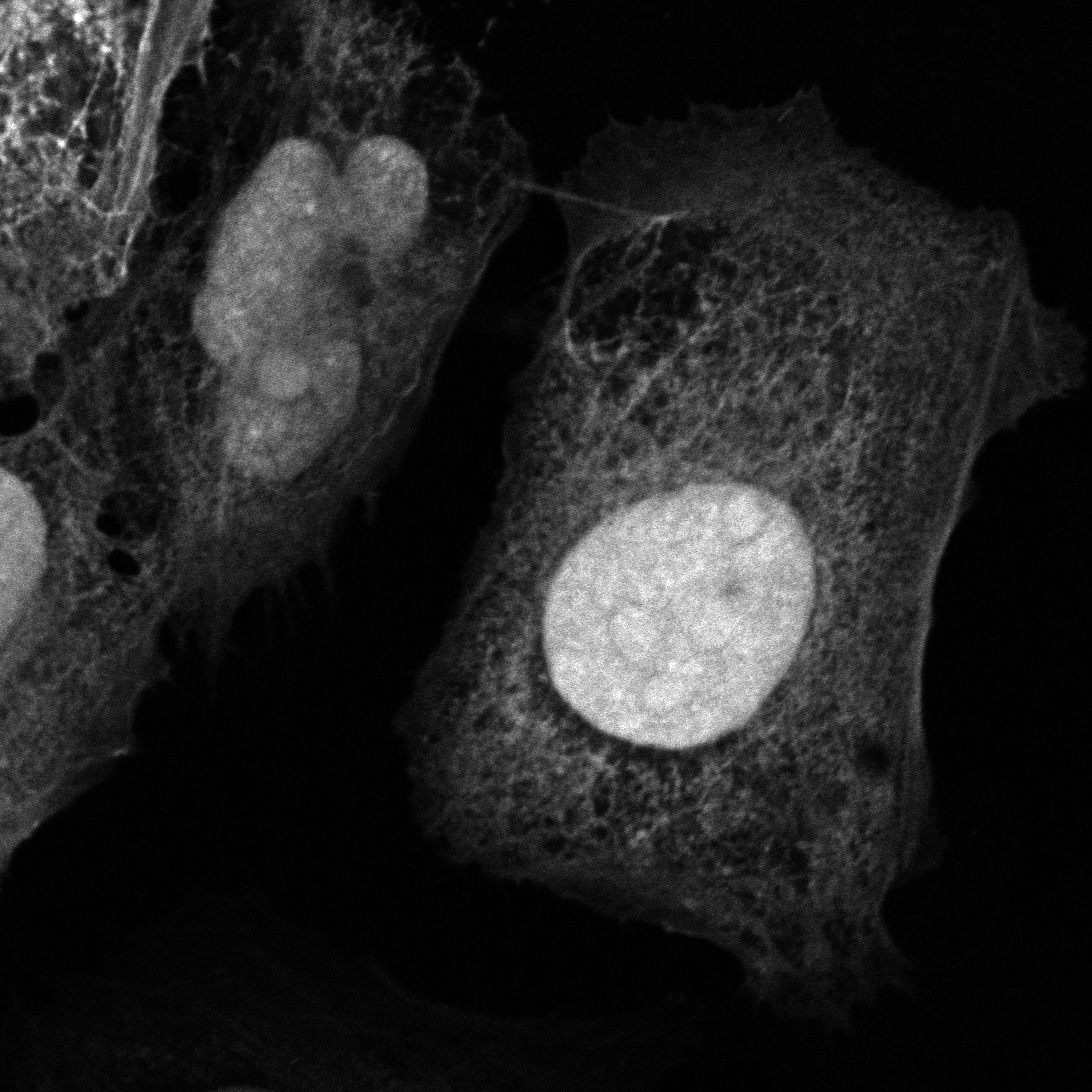

Supplement: Supplementary file 22 — Source Data for Figure 4 [file EMBJ-42-e114473-s001.zip › Figure_4/4G/GFP.tif]

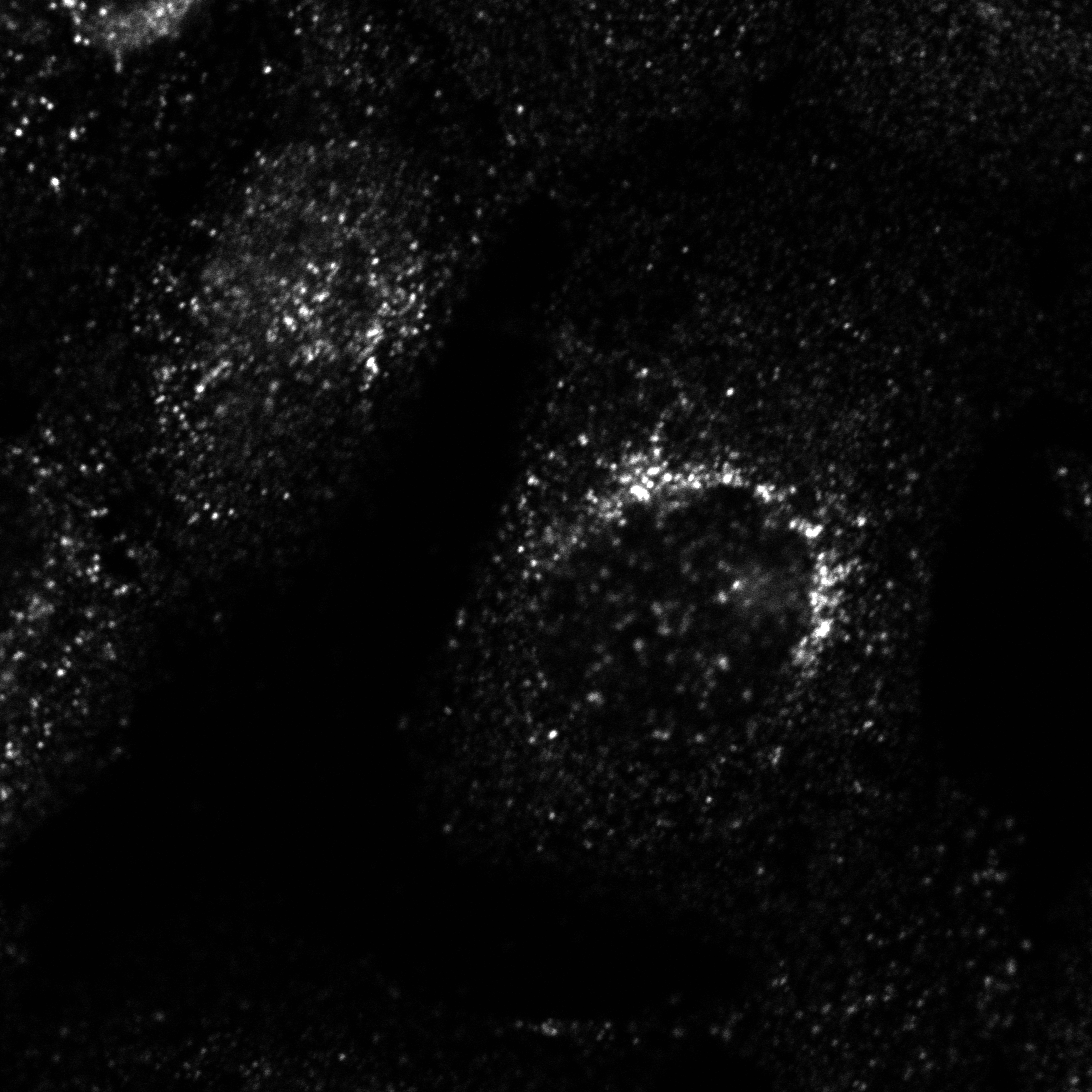

Supplement: Supplementary file 22 — Source Data for Figure 4 [file EMBJ-42-e114473-s001.zip › Figure_4/4G/GFP_AP1_gamma.tif]

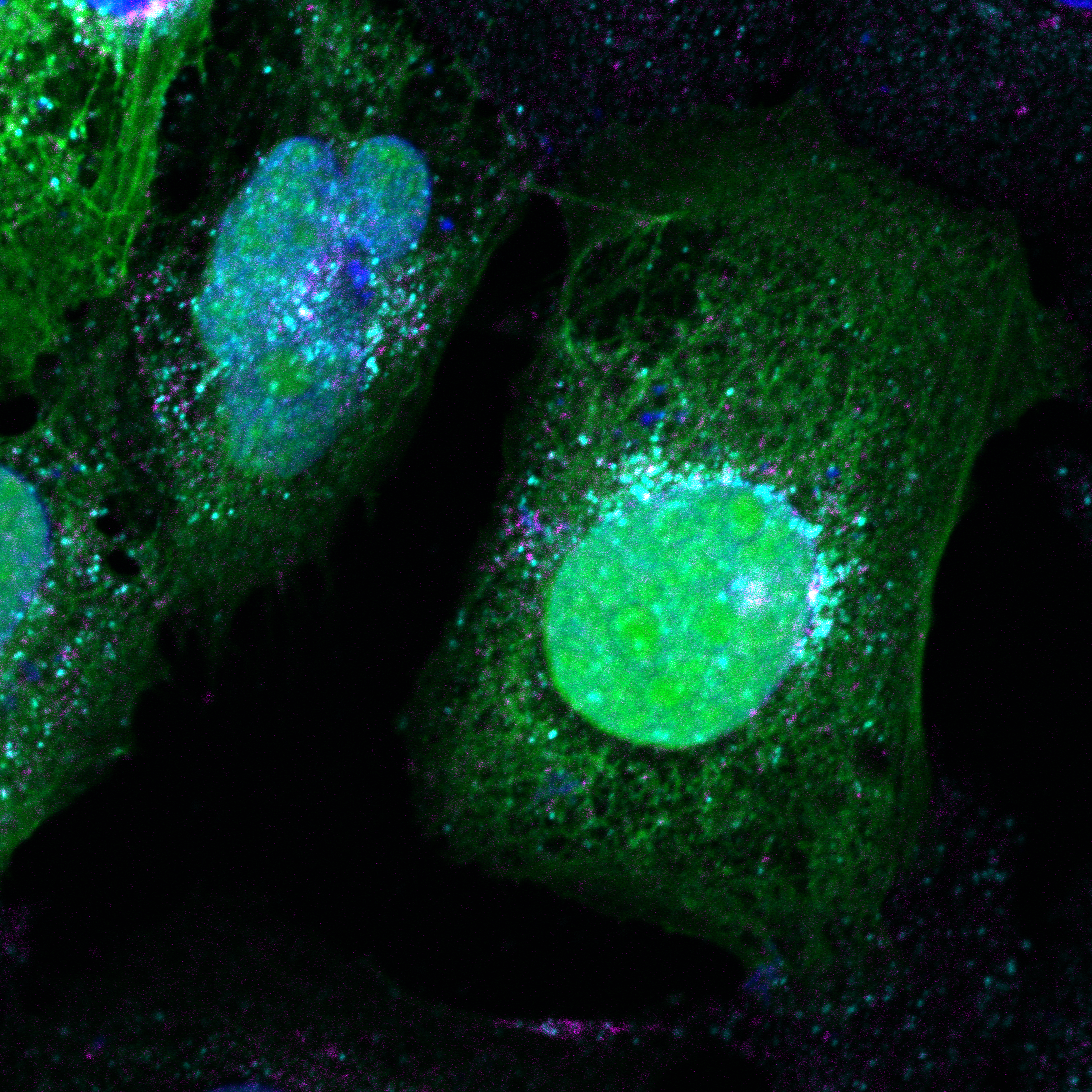

Supplement: Supplementary file 22 — Source Data for Figure 4 [file EMBJ-42-e114473-s001.zip › Figure_4/4G/GFP_merge.tiff]

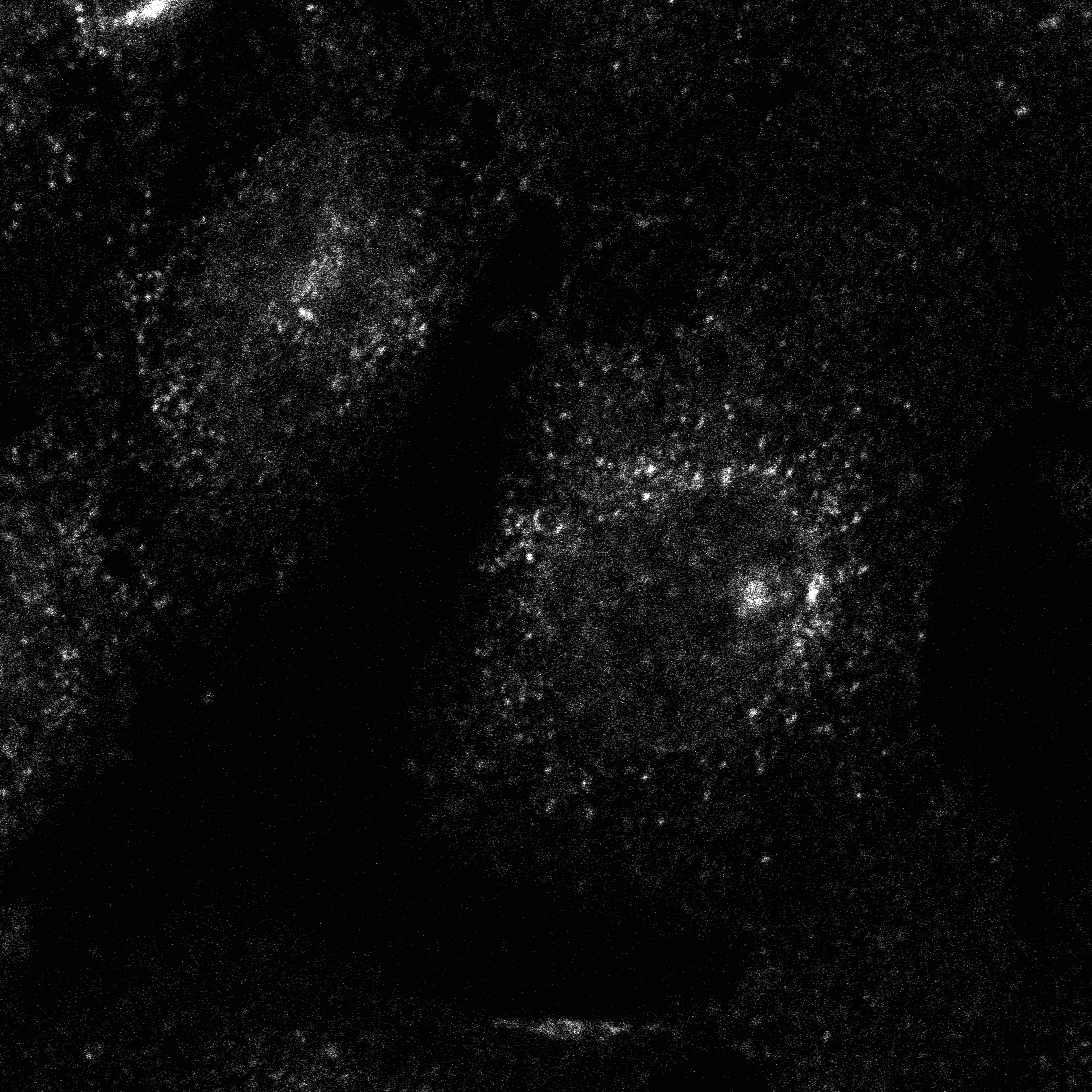

Supplement: Supplementary file 22 — Source Data for Figure 4 [file EMBJ-42-e114473-s001.zip › Figure_4/4G/GFP_Rab11.tif]

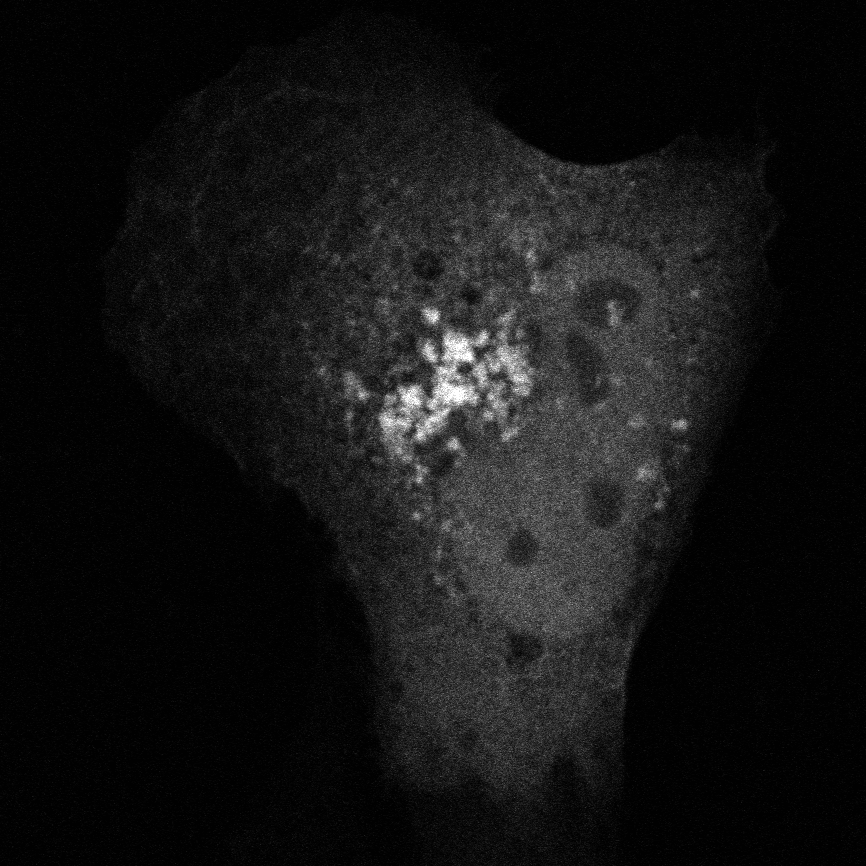

Supplement: Supplementary file 22 — Source Data for Figure 4 [file EMBJ-42-e114473-s001.zip › Figure_4/4H/GFP-HR5B.tif]

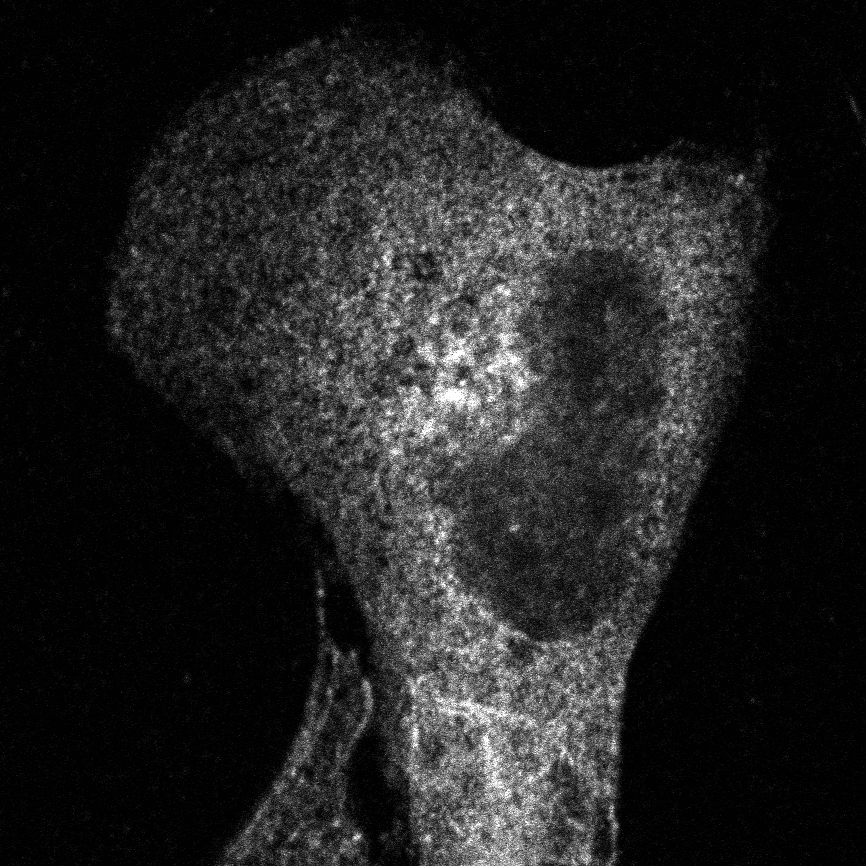

Supplement: Supplementary file 22 — Source Data for Figure 4 [file EMBJ-42-e114473-s001.zip › Figure_4/4H/GFP-HR5B_DCTN1.tif]

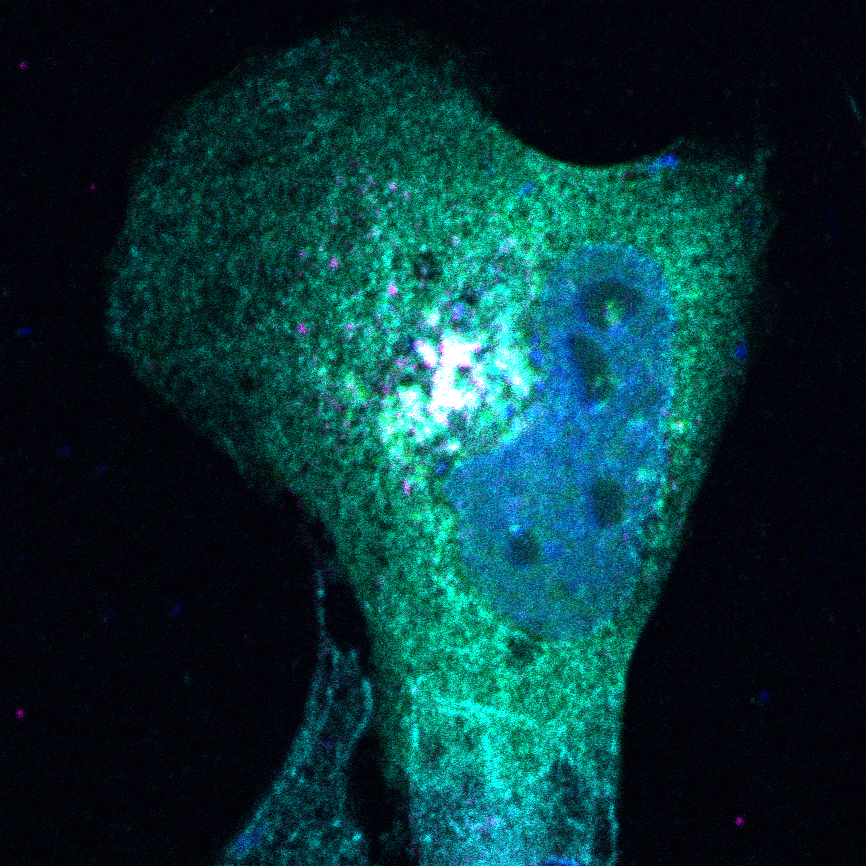

Supplement: Supplementary file 22 — Source Data for Figure 4 [file EMBJ-42-e114473-s001.zip › Figure_4/4H/GFP-HR5B_merge.tiff]

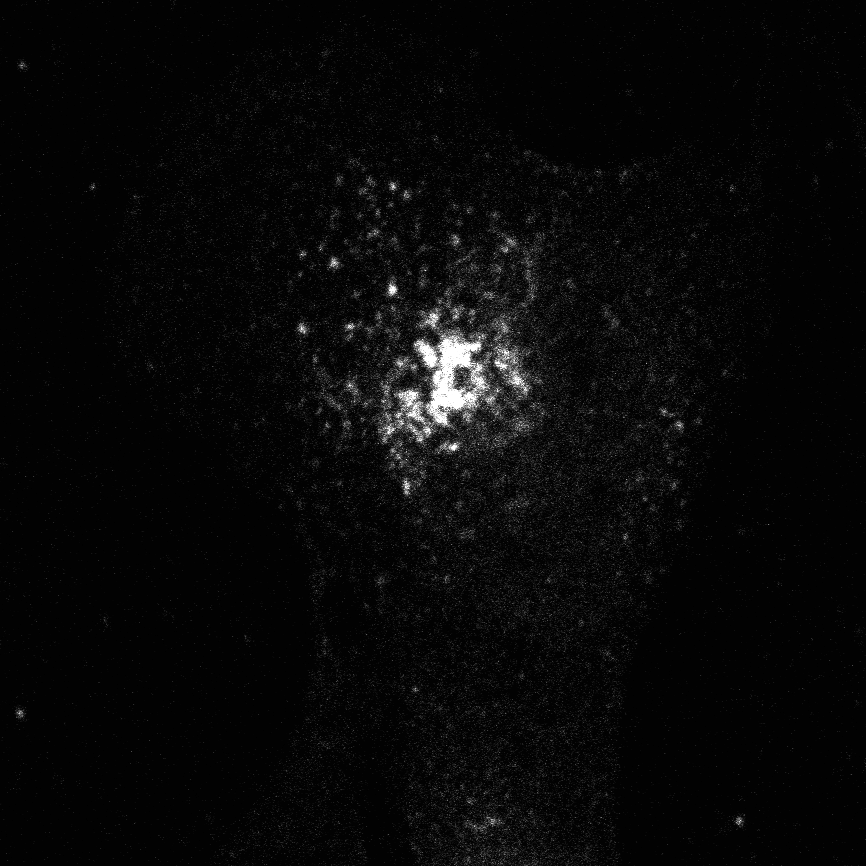

Supplement: Supplementary file 22 — Source Data for Figure 4 [file EMBJ-42-e114473-s001.zip › Figure_4/4H/GFP-HR5B_Rab11.tif]

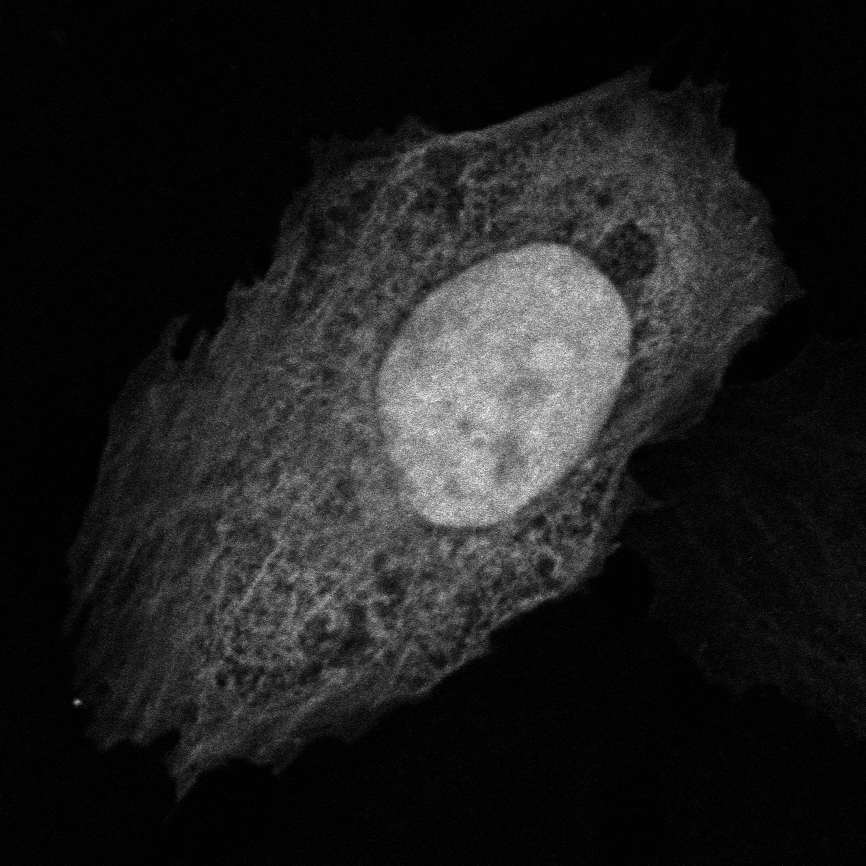

Supplement: Supplementary file 22 — Source Data for Figure 4 [file EMBJ-42-e114473-s001.zip › Figure_4/4H/GFP.tif]

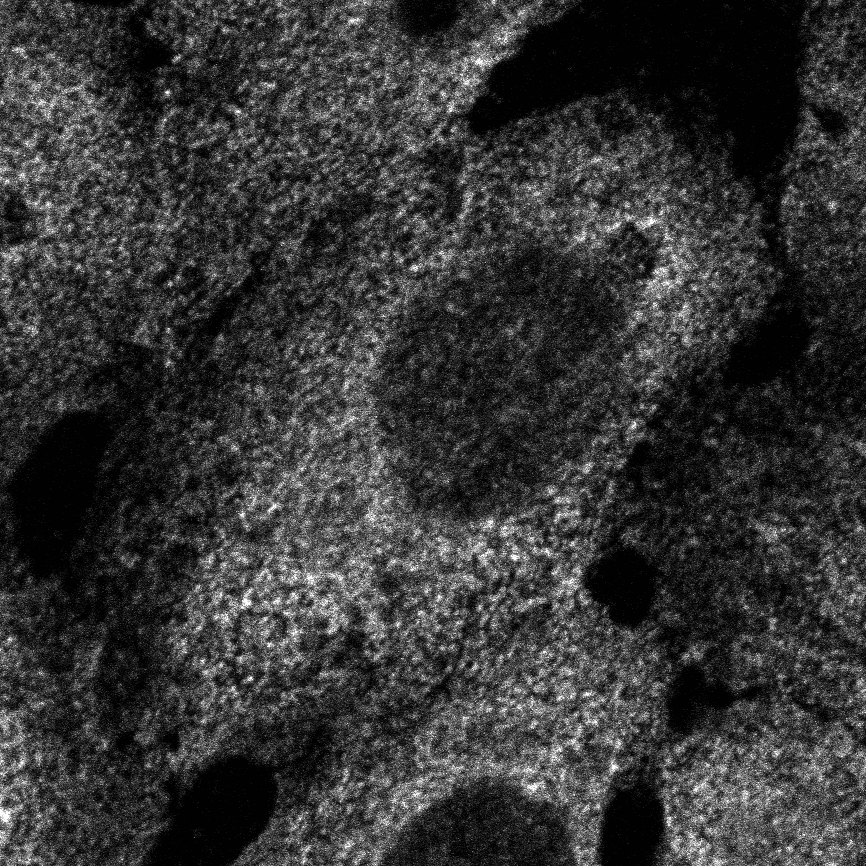

Supplement: Supplementary file 22 — Source Data for Figure 4 [file EMBJ-42-e114473-s001.zip › Figure_4/4H/GFP_DCTN1.tif]

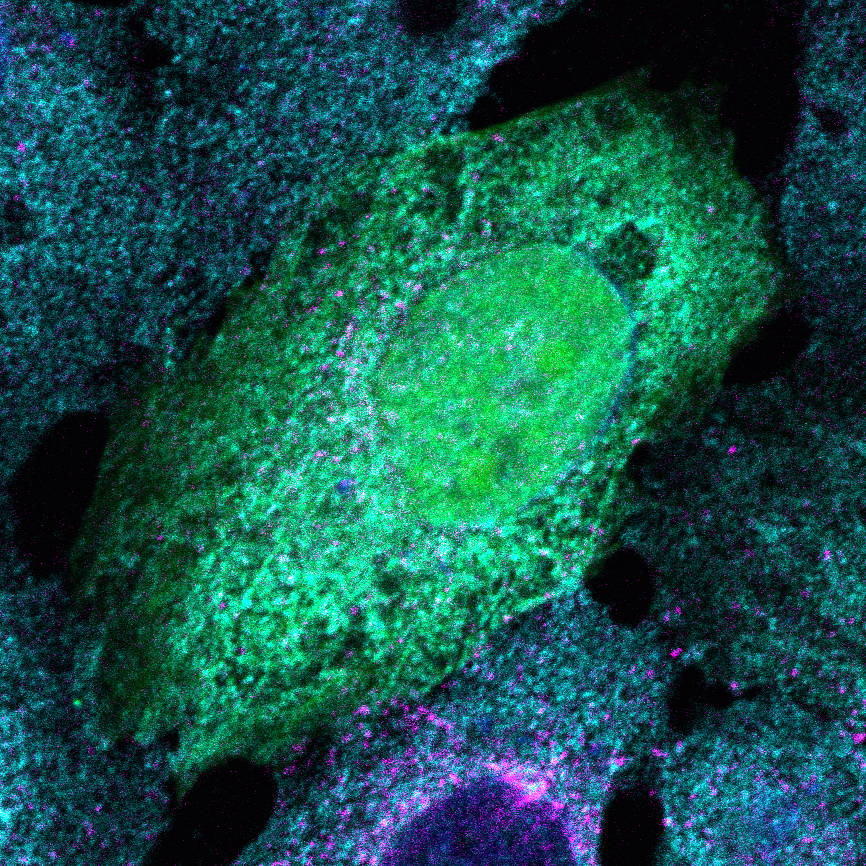

Supplement: Supplementary file 22 — Source Data for Figure 4 [file EMBJ-42-e114473-s001.zip › Figure_4/4H/GFP_merge.tiff]

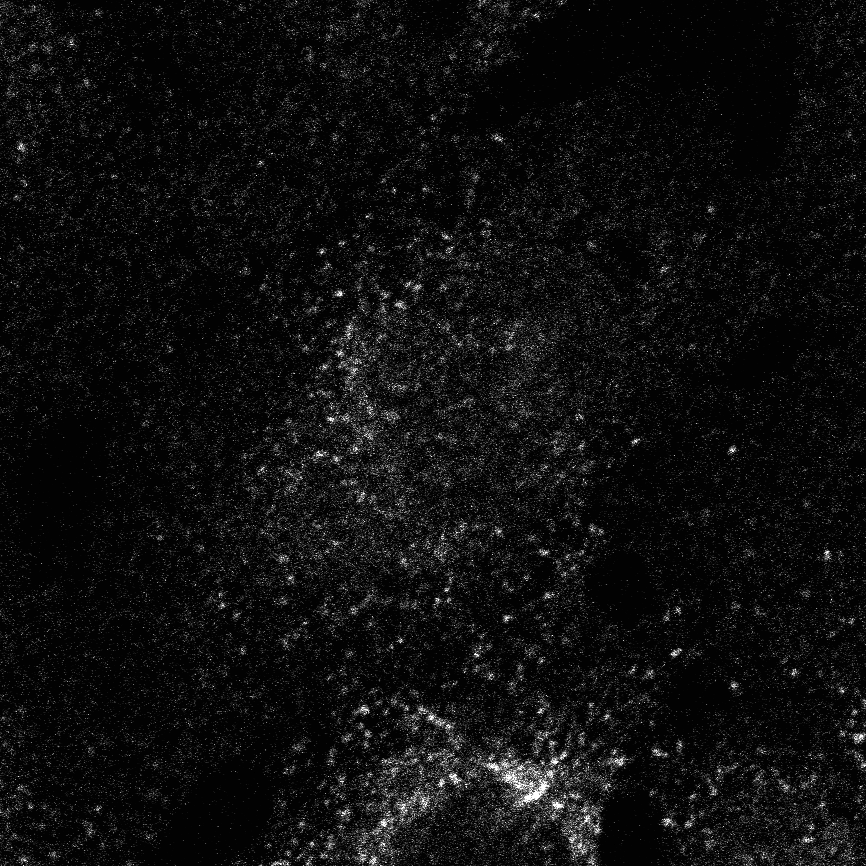

Supplement: Supplementary file 22 — Source Data for Figure 4 [file EMBJ-42-e114473-s001.zip › Figure_4/4H/GFP_Rab11.tif]

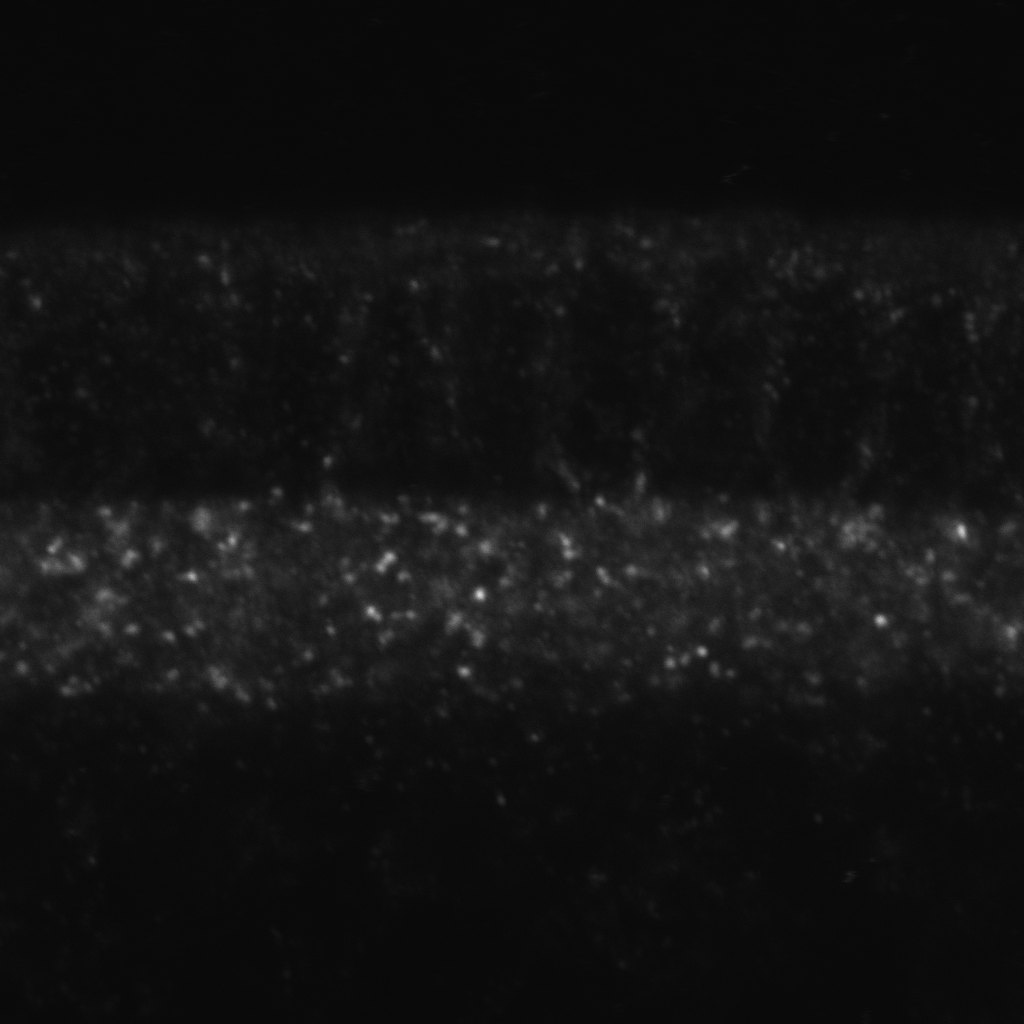

Supplement: Supplementary file 23 — Source Data for Figure 5 [file EMBJ-42-e114473-s013.zip › Figure_5/5C/AP1g_control.tif]

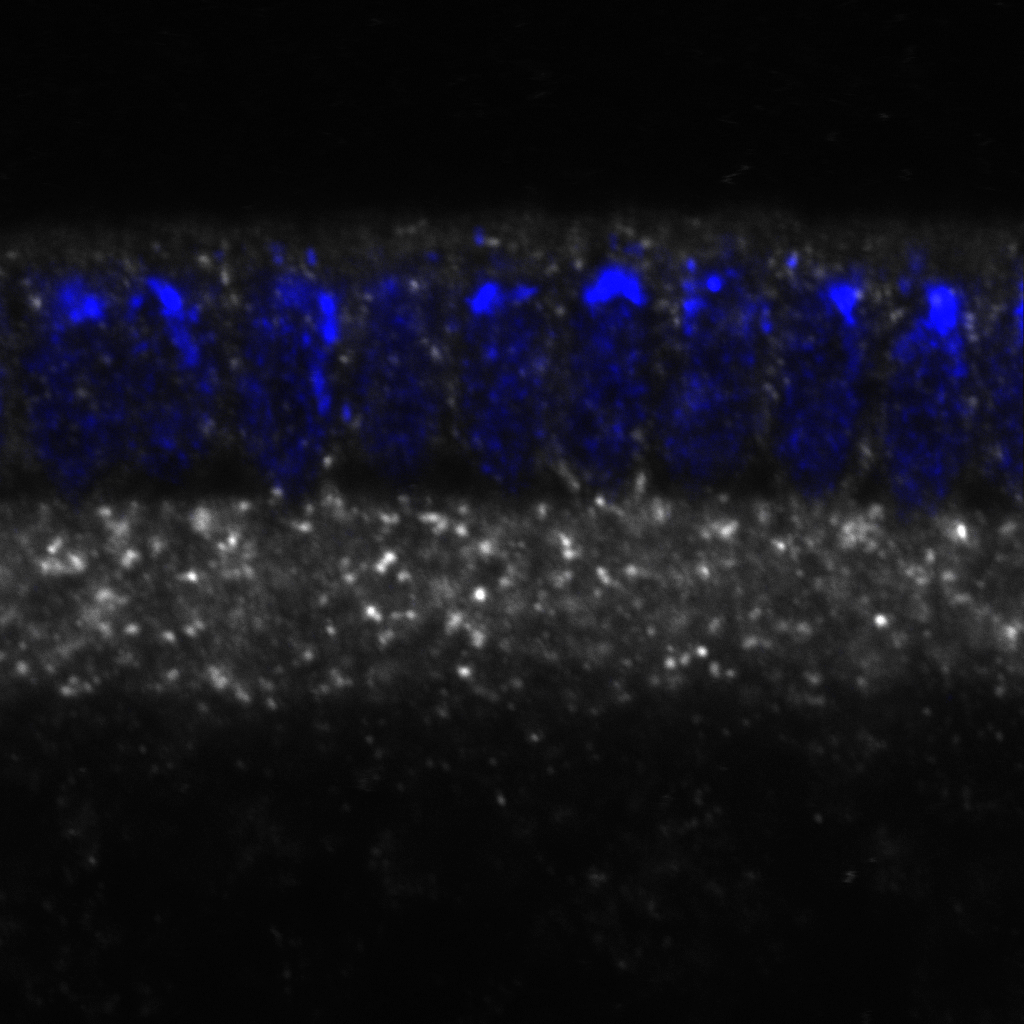

Supplement: Supplementary file 23 — Source Data for Figure 5 [file EMBJ-42-e114473-s013.zip › Figure_5/5C/AP1g_DAPI_control.tif]

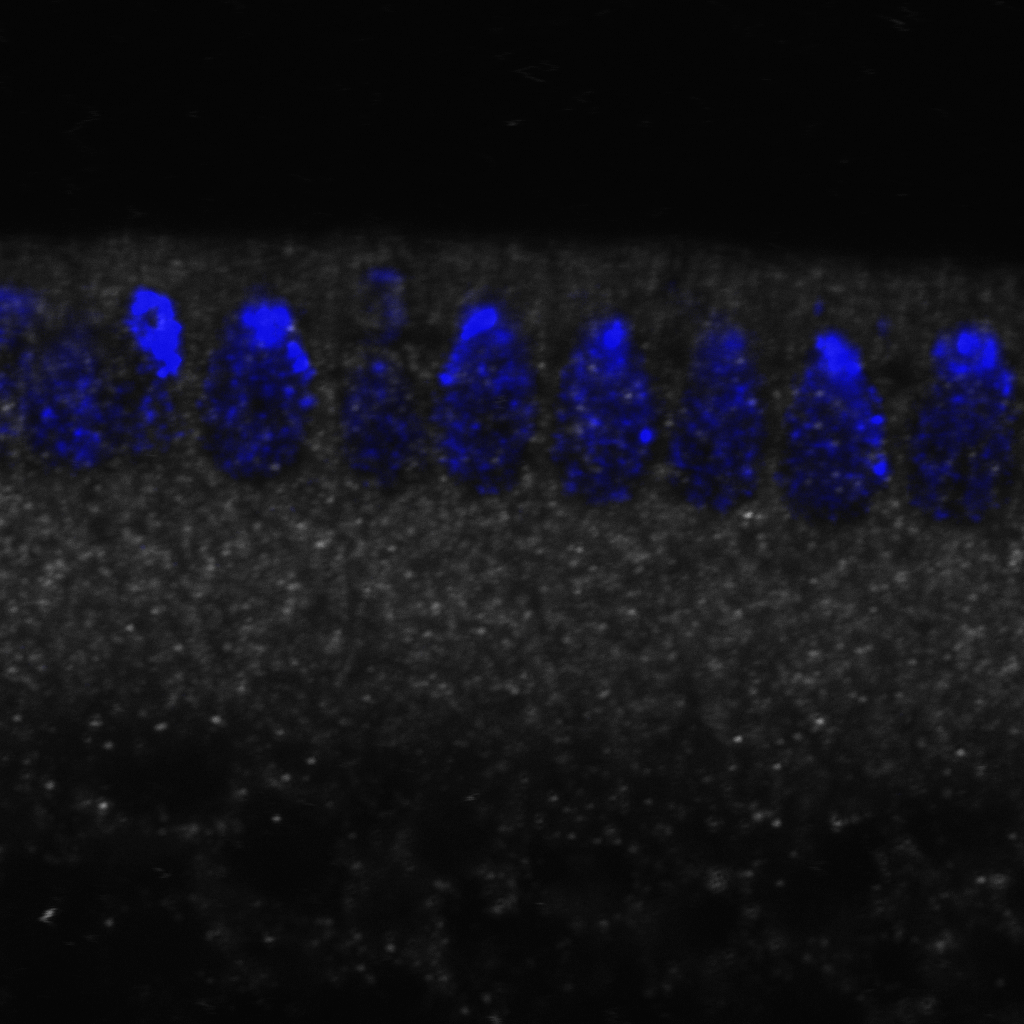

Supplement: Supplementary file 23 — Source Data for Figure 5 [file EMBJ-42-e114473-s013.zip › Figure_5/5C/AP1g_DAPI_hr5gRNA1+2.tif]

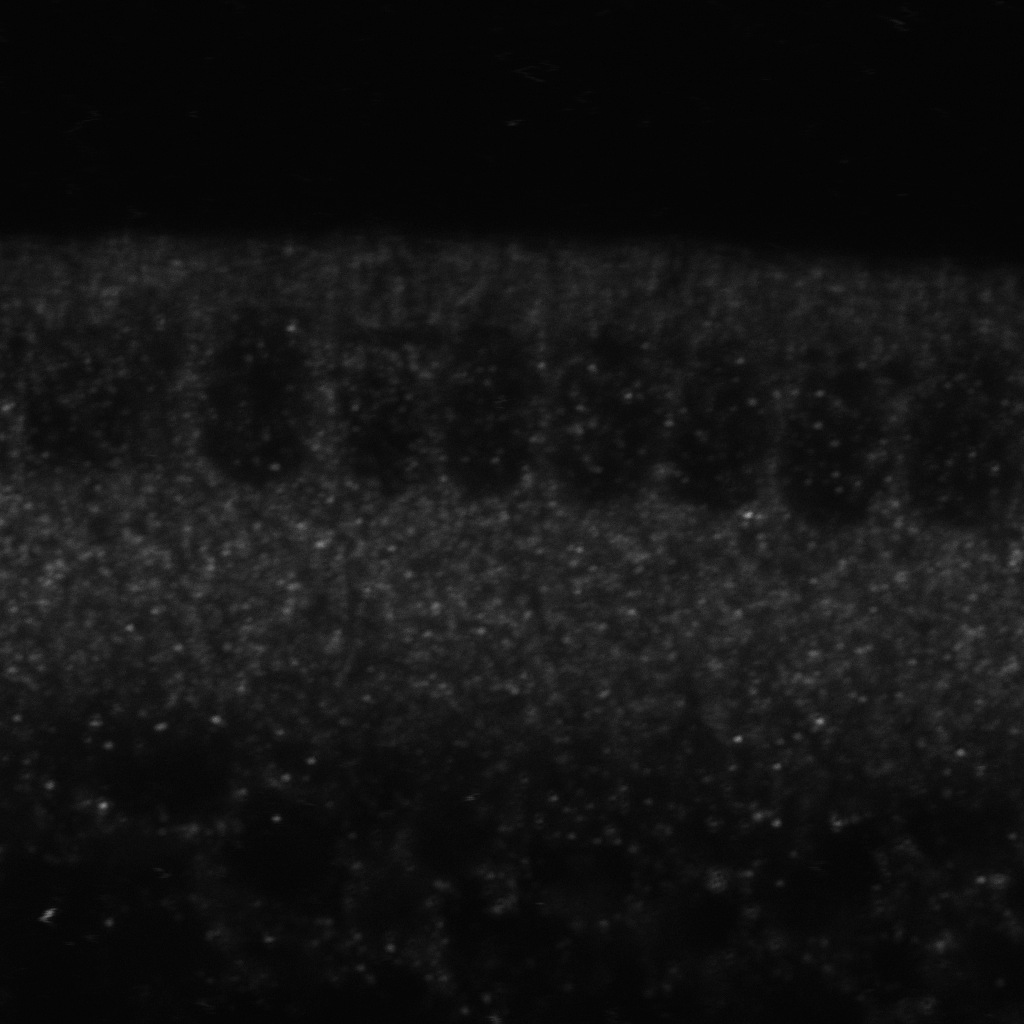

Supplement: Supplementary file 23 — Source Data for Figure 5 [file EMBJ-42-e114473-s013.zip › Figure_5/5C/AP1g_hr5gRNA1+2.tif]

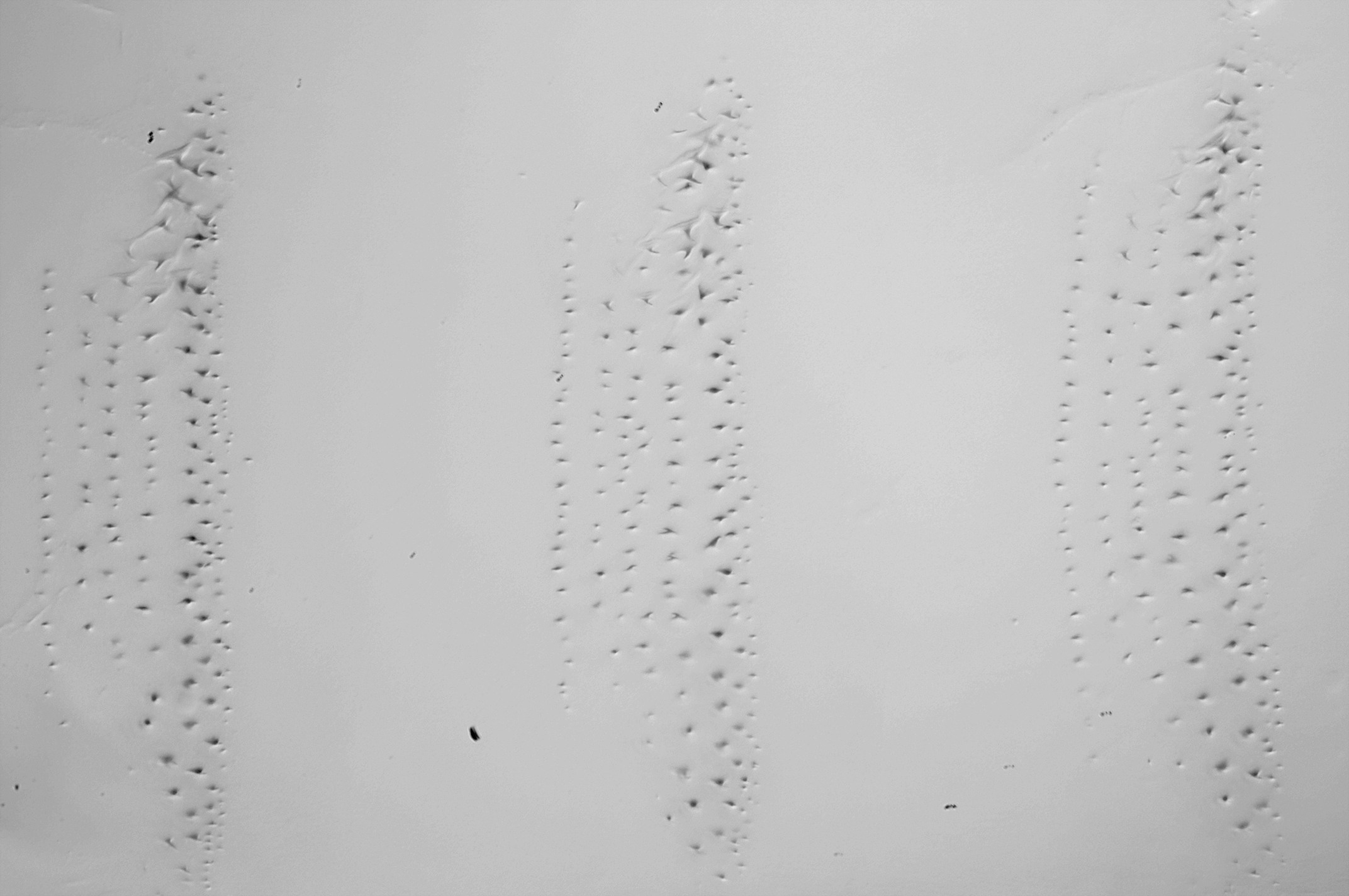

Supplement: Supplementary file 23 — Source Data for Figure 5 [file EMBJ-42-e114473-s013.zip › Figure_5/5C/cuticle_control.tif]

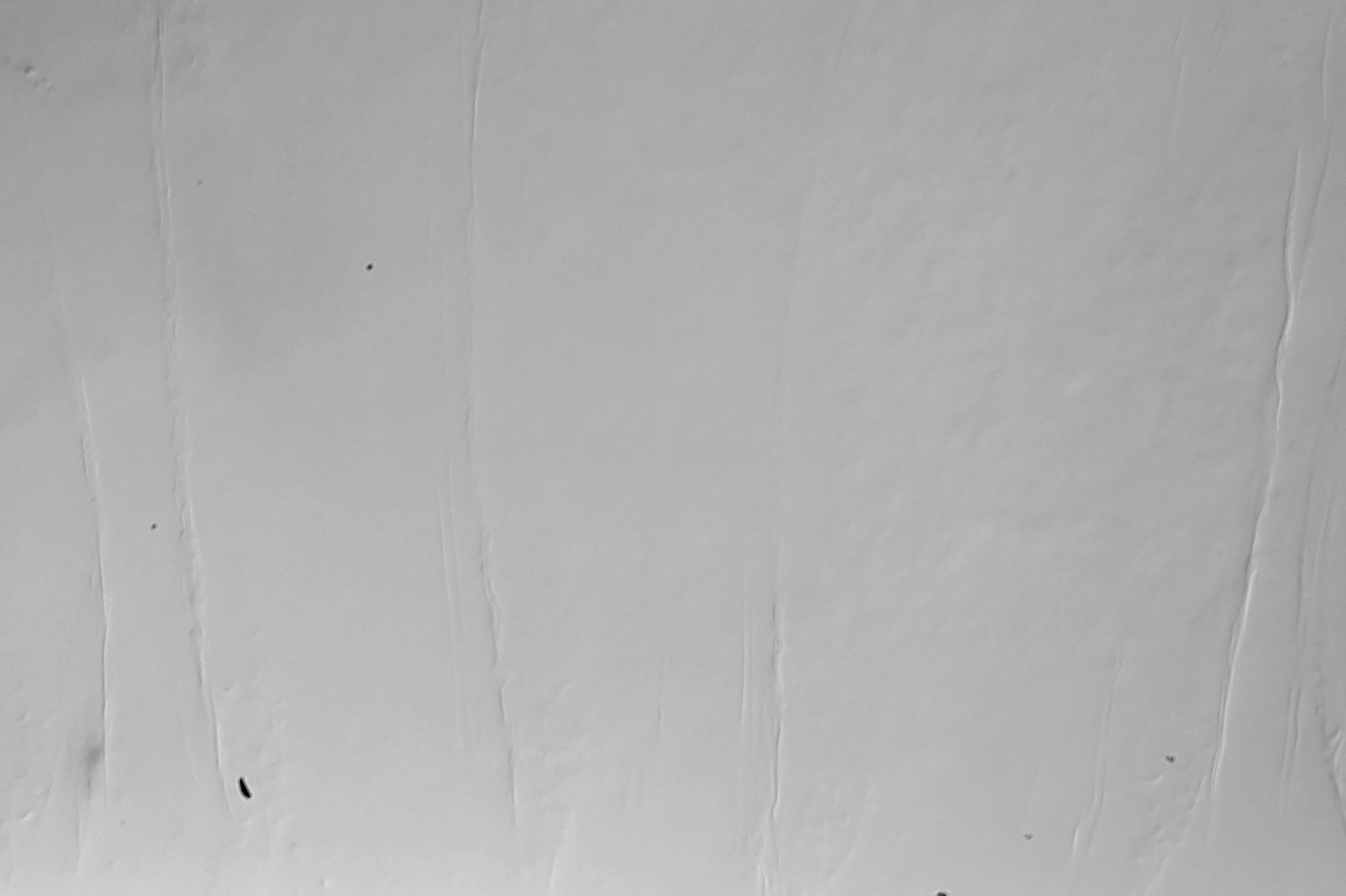

Supplement: Supplementary file 23 — Source Data for Figure 5 [file EMBJ-42-e114473-s013.zip › Figure_5/5C/cuticle_hr5gRNA1+2.tif]

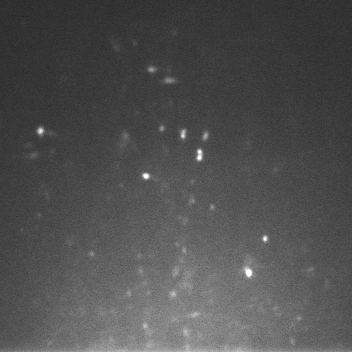

Supplement: Supplementary file 24 — Source Data for Figure 6 [file EMBJ-42-e114473-s012.zip › Figure_6/6A/555AP1_control_150_A.tif]

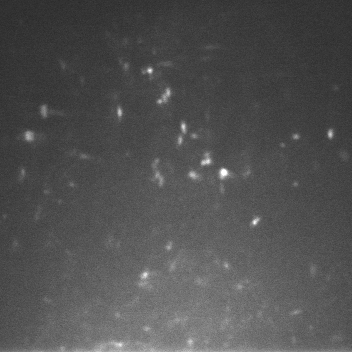

Supplement: Supplementary file 24 — Source Data for Figure 6 [file EMBJ-42-e114473-s012.zip › Figure_6/6A/555AP1_control_210_A.tif]

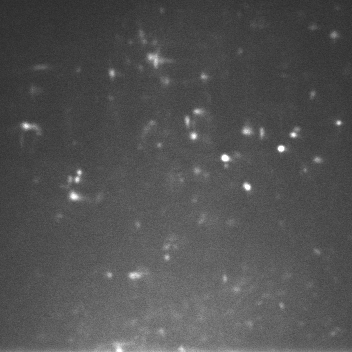

Supplement: Supplementary file 24 — Source Data for Figure 6 [file EMBJ-42-e114473-s012.zip › Figure_6/6A/555AP1_control_270_A.tif]

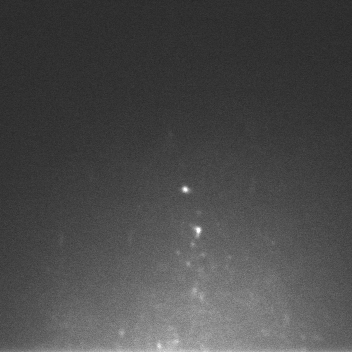

Supplement: Supplementary file 24 — Source Data for Figure 6 [file EMBJ-42-e114473-s012.zip › Figure_6/6A/555AP1_control_30_A.tif]

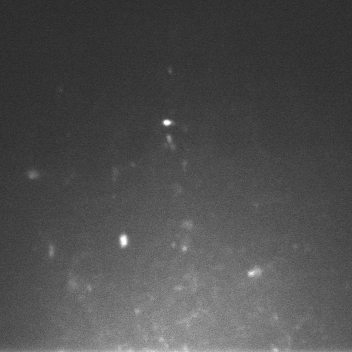

Supplement: Supplementary file 24 — Source Data for Figure 6 [file EMBJ-42-e114473-s012.zip › Figure_6/6A/555AP1_control_90_A.tif]

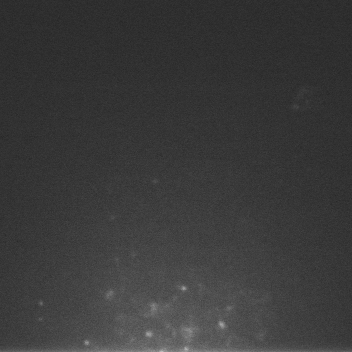

Supplement: Supplementary file 24 — Source Data for Figure 6 [file EMBJ-42-e114473-s012.zip › Figure_6/6A/555AP1_gRNAHr5_1+2_150_A.tif]

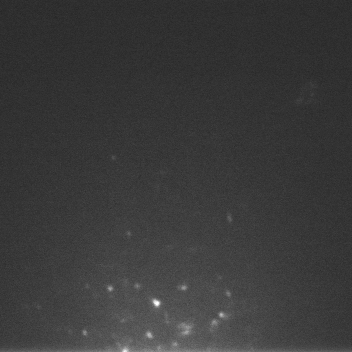

Supplement: Supplementary file 24 — Source Data for Figure 6 [file EMBJ-42-e114473-s012.zip › Figure_6/6A/555AP1_gRNAHr5_1+2_210_A.tif]

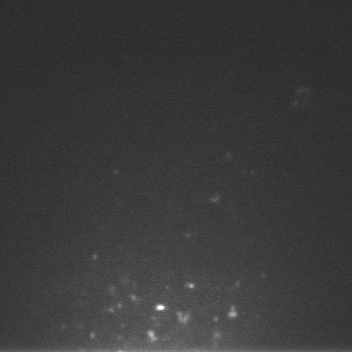

Supplement: Supplementary file 24 — Source Data for Figure 6 [file EMBJ-42-e114473-s012.zip › Figure_6/6A/555AP1_gRNAHr5_1+2_270_A.tif]

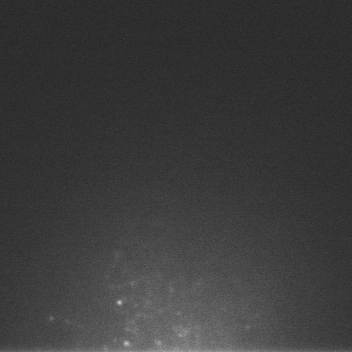

Supplement: Supplementary file 24 — Source Data for Figure 6 [file EMBJ-42-e114473-s012.zip › Figure_6/6A/555AP1_gRNAHr5_1+2_30_A.tif]

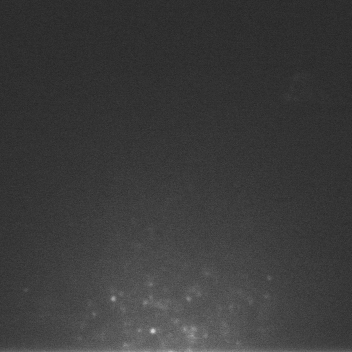

Supplement: Supplementary file 24 — Source Data for Figure 6 [file EMBJ-42-e114473-s012.zip › Figure_6/6A/555AP1_gRNAHr5_1+2_90_A.tif]

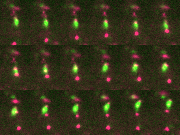

Supplement: Supplementary file 25 — Source Data for Figure 7 [file EMBJ-42-e114473-s017.zip › Figure_7/7A/GFP-G245_AP1g_ImageJmontage.tif]

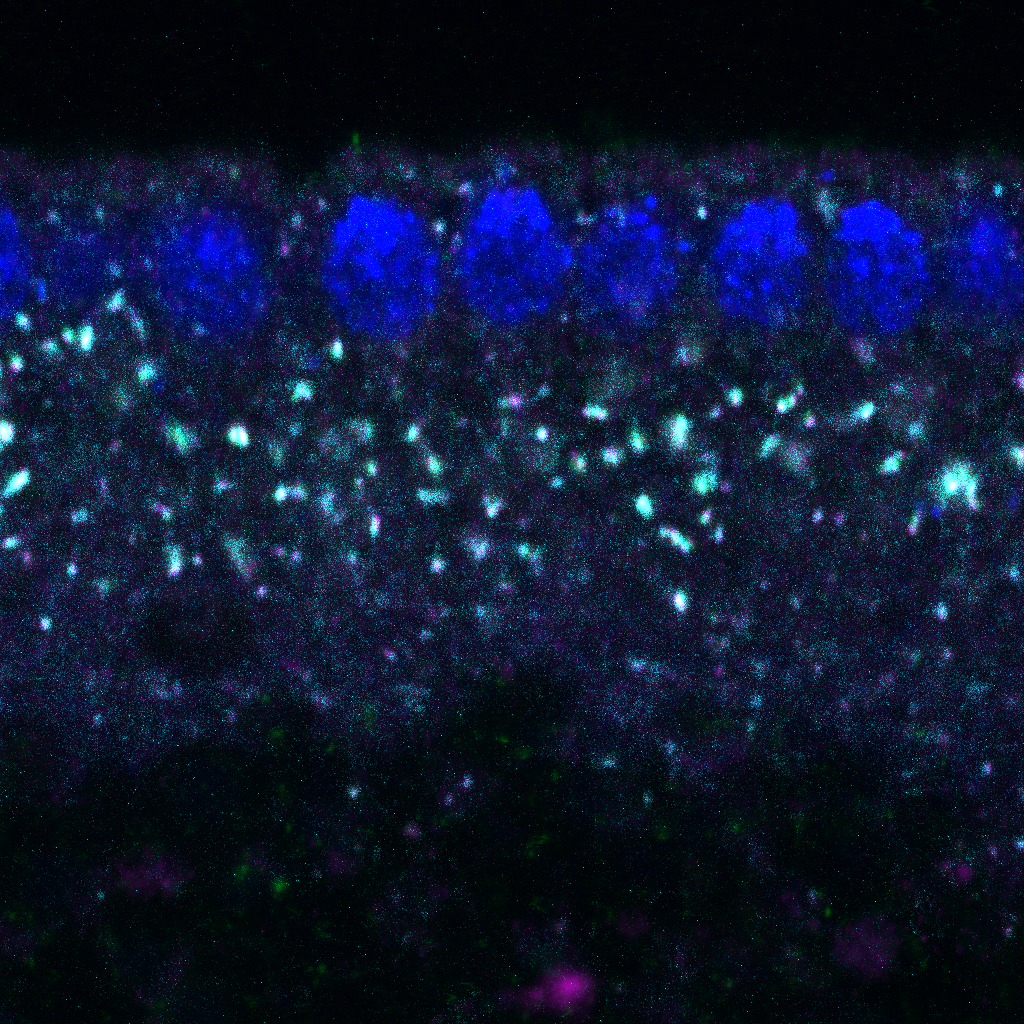

Supplement: Supplementary file 25 — Source Data for Figure 7 [file EMBJ-42-e114473-s017.zip › Figure_7/7B/Composite_G84_GM130_G245_control.tif]

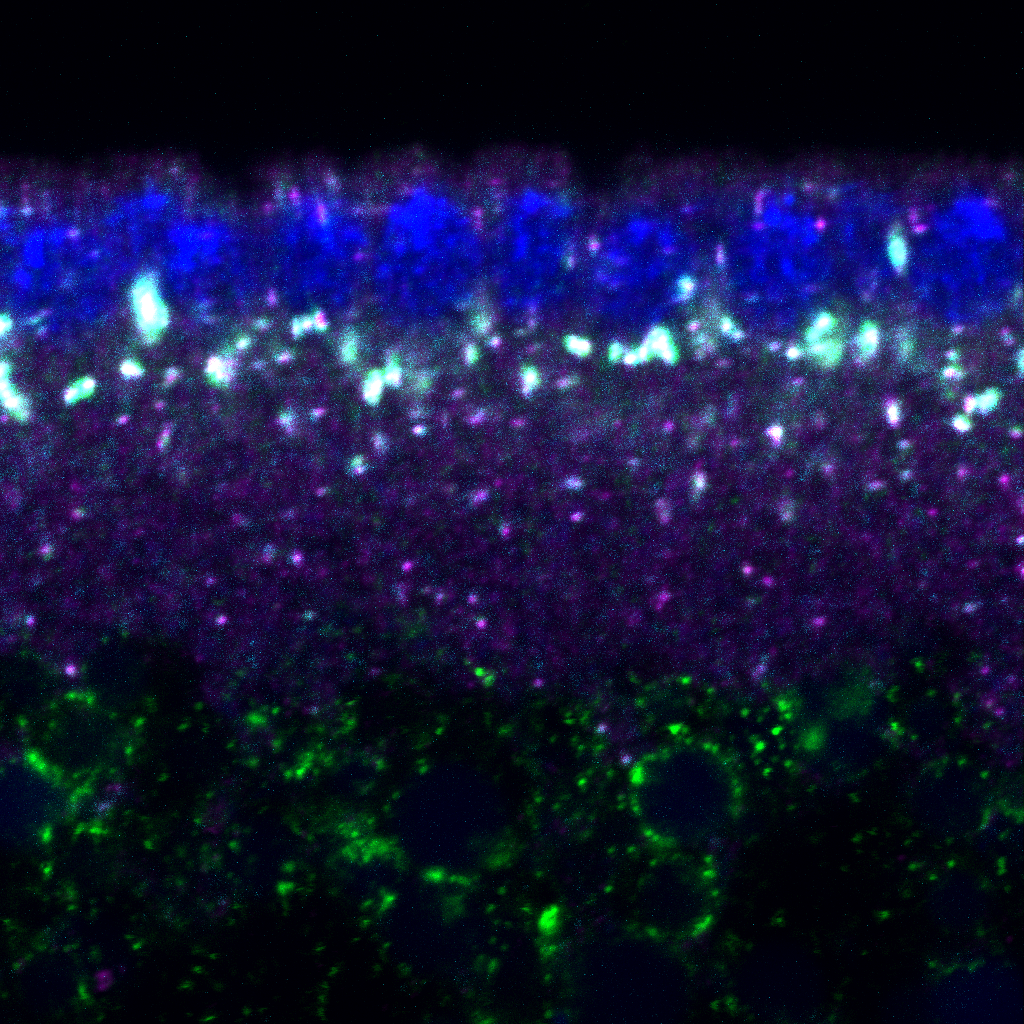

Supplement: Supplementary file 25 — Source Data for Figure 7 [file EMBJ-42-e114473-s017.zip › Figure_7/7B/Composite_G84_GM130_G245_gRNAHr5.tif]

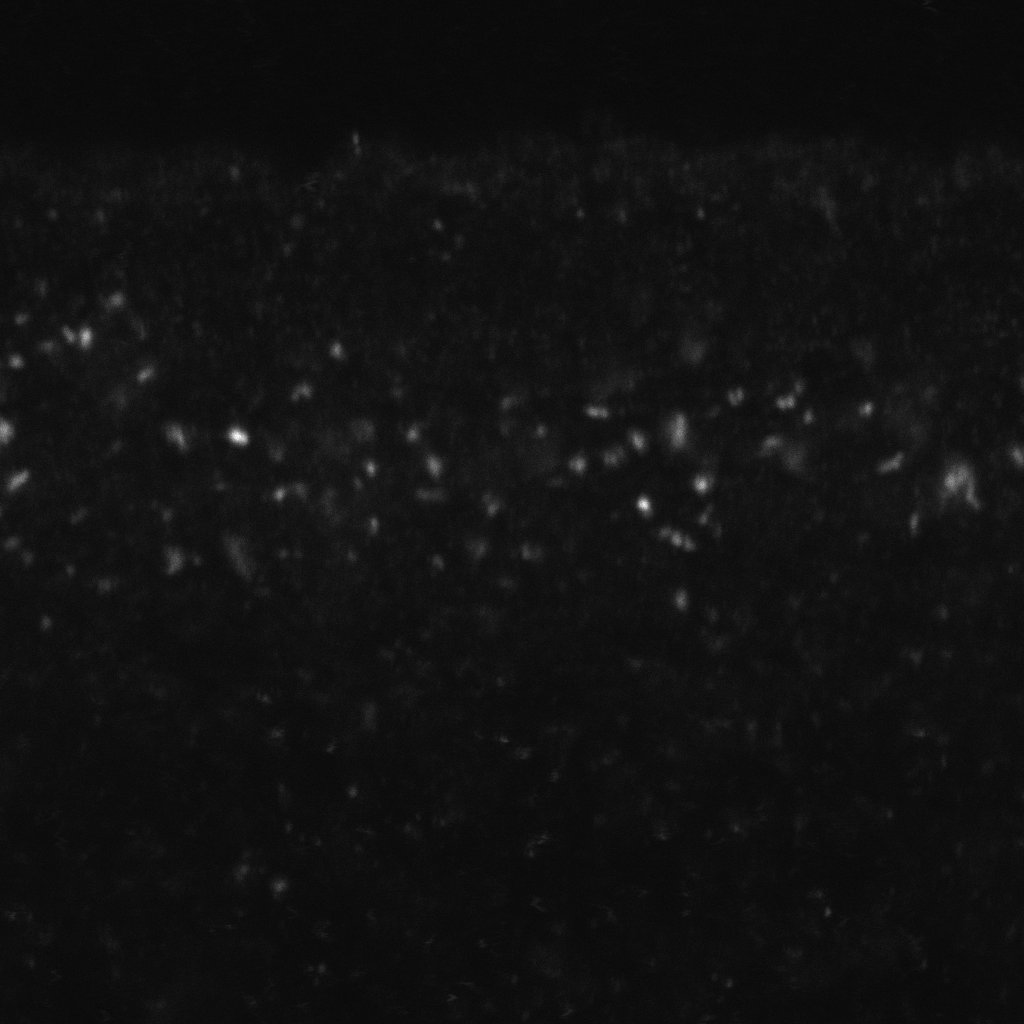

Supplement: Supplementary file 25 — Source Data for Figure 7 [file EMBJ-42-e114473-s017.zip › Figure_7/7B/G245_control.tif]

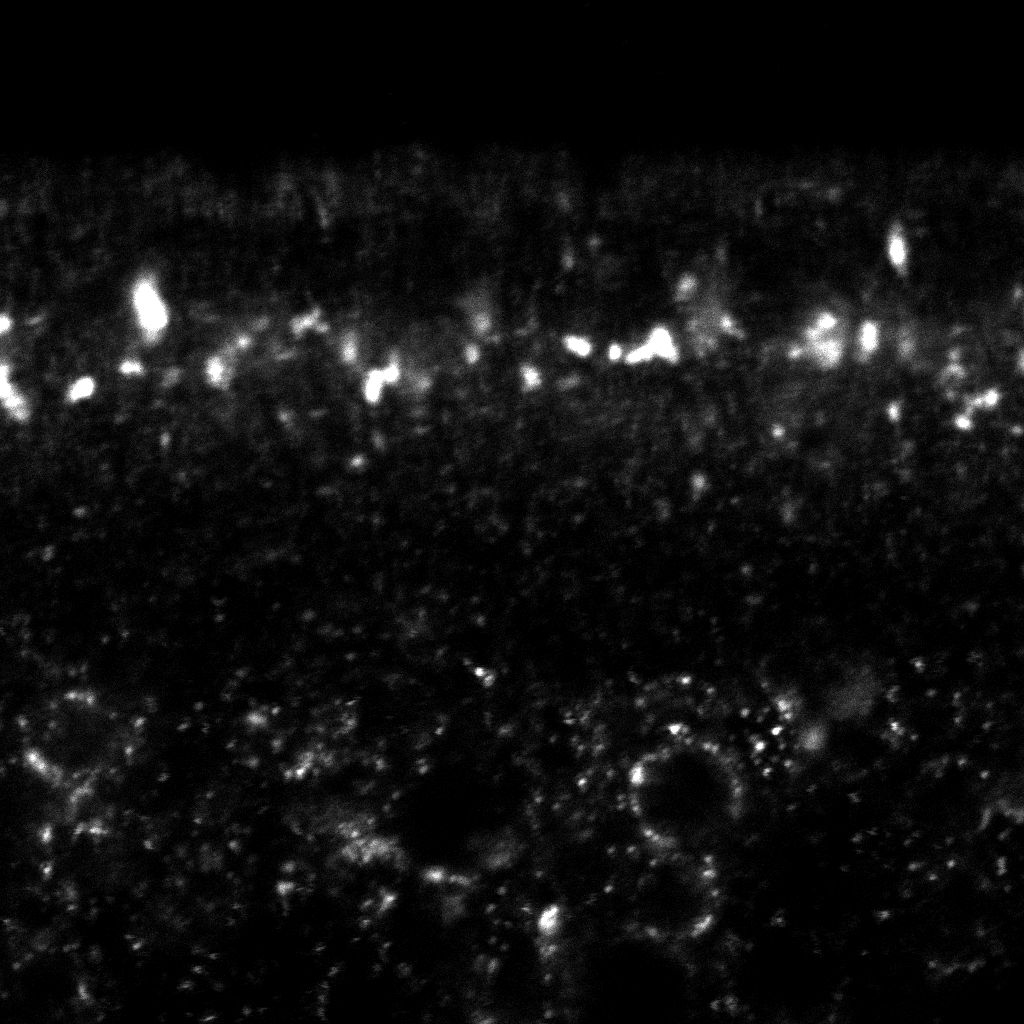

Supplement: Supplementary file 25 — Source Data for Figure 7 [file EMBJ-42-e114473-s017.zip › Figure_7/7B/G245_gRNAHr5.tif]

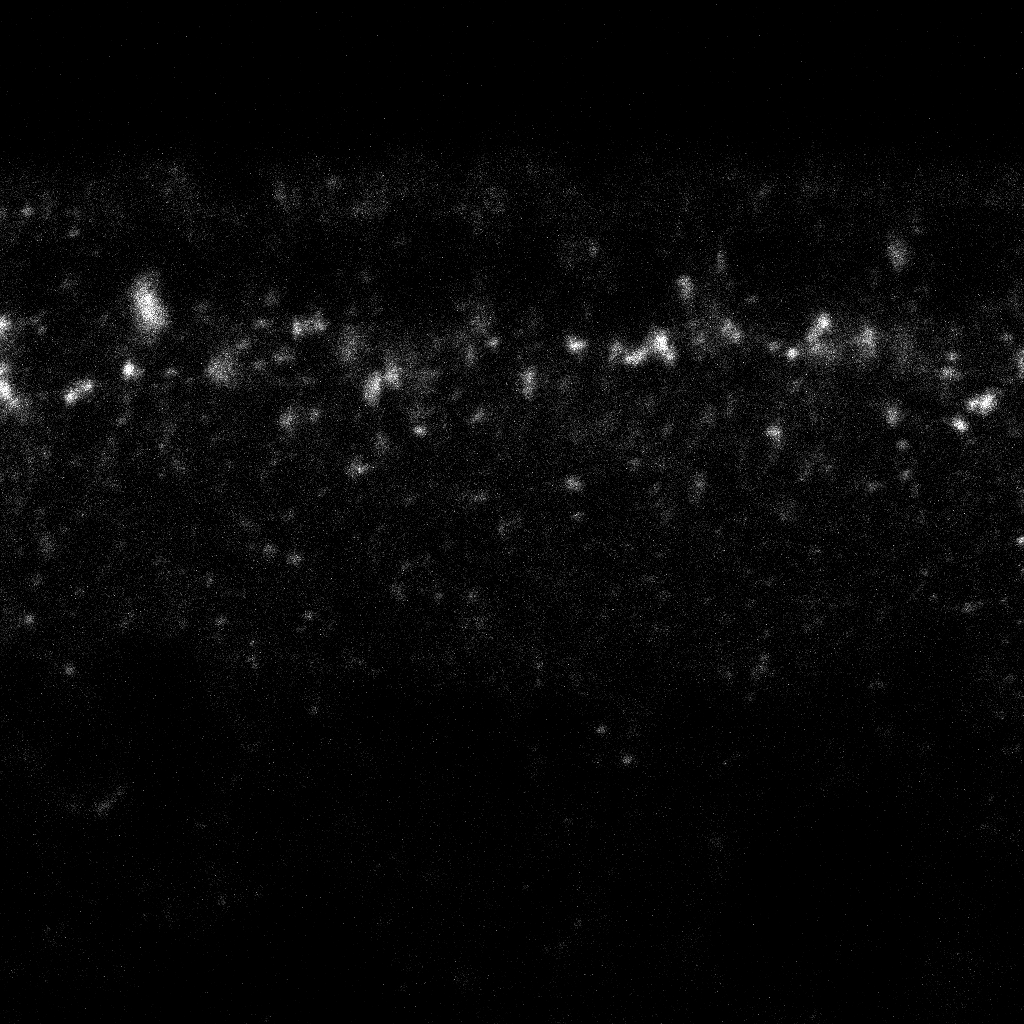

Supplement: Supplementary file 25 — Source Data for Figure 7 [file EMBJ-42-e114473-s017.zip › Figure_7/7B/G84_gRNAHr5.tif]

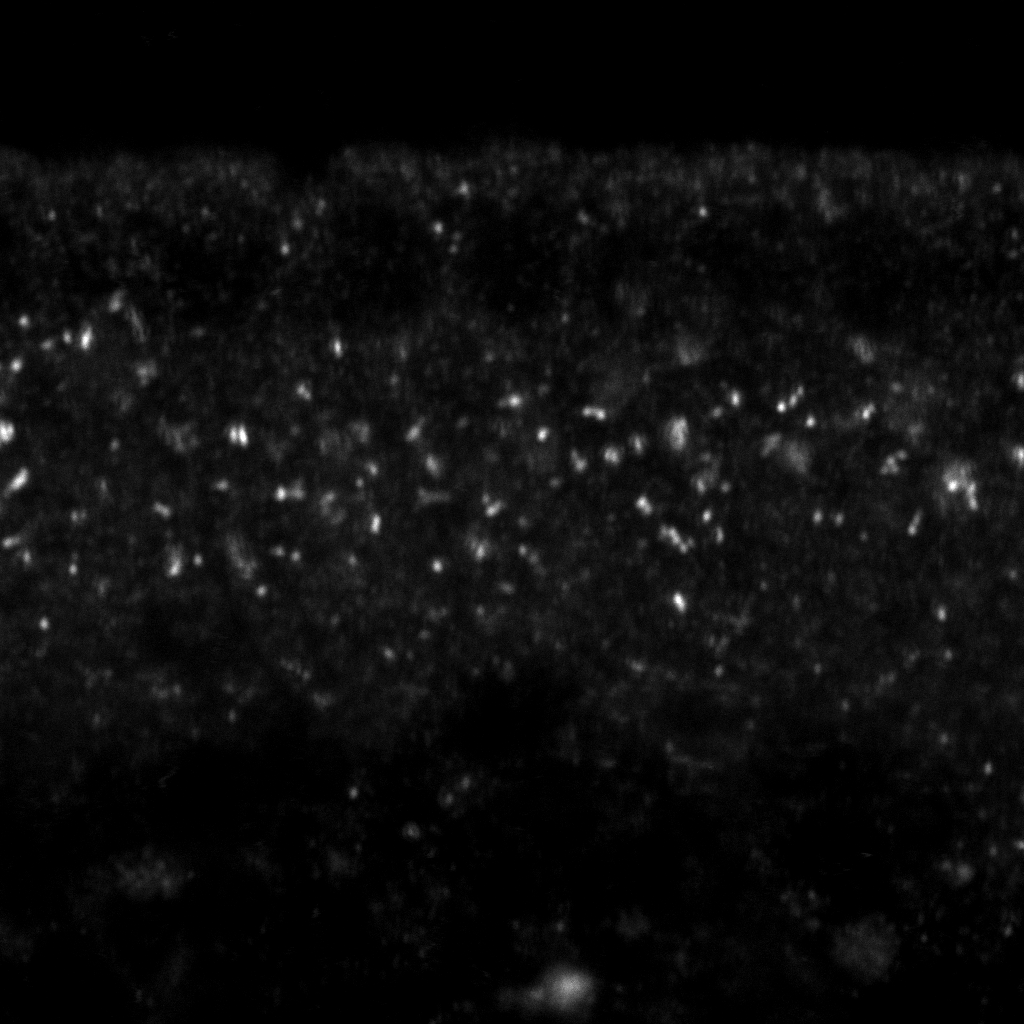

Supplement: Supplementary file 25 — Source Data for Figure 7 [file EMBJ-42-e114473-s017.zip › Figure_7/7B/GM130_control.tif]

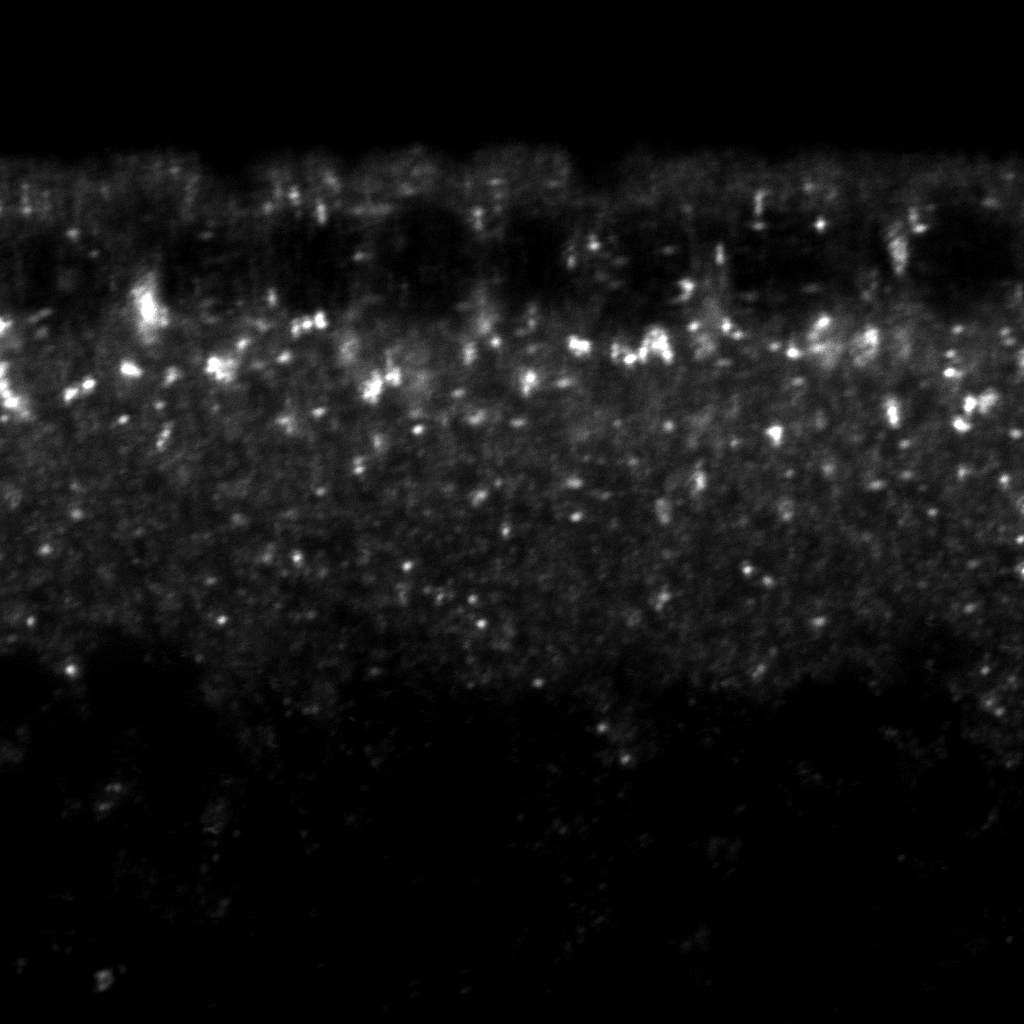

Supplement: Supplementary file 25 — Source Data for Figure 7 [file EMBJ-42-e114473-s017.zip › Figure_7/7B/GM130_gRNAHr5.tif]
