# Supplementary material for: HEATR5B associates with dynein‐dynactin and promotes motility of AP1‐bound endosomal membranes
Source: EMBO J. 2023 Oct 24;42(23):e114473. doi: 10.15252/embj.2023114473 (PMC10690479; doi:10.15252/embj.2023114473)
Supplement: Supplementary file 25 — Source Data for Figure 7 [file EMBJ-42-e114473-s017.zip › Figure_7/7A/README_7A.rtf]

Created from Movie EV10 using Montage tool in Fiji. In Figure 7A, white lines have been added using Adobe Illustrator to assist with delineation of panels. 
