# Supplementary material for: HEATR5B associates with dynein‐dynactin and promotes motility of AP1‐bound endosomal membranes
Source: EMBO J. 2023 Oct 24;42(23):e114473. doi: 10.15252/embj.2023114473 (PMC10690479; doi:10.15252/embj.2023114473)
Supplement: Supplementary file 26 [file EMBJ-42-e114473-s015.pdf]

THE EMBO JOURNAL

HEATR5B associates with dynein-dynactin and promotes motility of AP1-bound endosomal membranes

Vanesa Madan, Lucas Albacete Albacete, Li Jin, Pietro Scaturro, Joseph Watson, Nadine Muschalik, Farida Begum, Jerome Boulanger, Karl Bauer, Michael Kiebler, Emmanuel Derivery, and Simon Bullock
DOI: 10.15252/embj.2023114473

Corresponding author(s): Simon Bullock (sbullock@mrc-lmb.cam.ac.uk)

Review Timeline:

Transfer from Review Commons:	9th May 23
Editorial Decision:	11th May 23
Revision Received:	25th Aug 23
Editorial Decision:	19th Sep 23
Revision Received:	20th Sep 23
Accepted:	26th Sep 23

Review COMMONS

Editor: William Teale

Transaction Report: This manuscript was transferred to The EMBO JOURNAL following peer review at Review Commons.

Review #1

1. Evidence, reproducibility and clarity:

Evidence, reproducibility and clarity (Required)

This work is the first systematic attempt to identify and characterize a diverse set of adapters that attach cytoplasmic dynein to its different cargoes and thus activate the motor. It is an important work because in animal cells dynein is the only efficient motor that can perform processive transport toward the minus ends of microtubules, and therefore the specificity of transport for multiple cargoes along microtubules is determined by these adapters.

The authors use the recombinant tail of dynein for pulling down interacting proteins from the cell extract. This is a straightforward approach, but its main problem is the large number of non-specific proteins that bind to the column. To solve the problem, the authors use a very smart approach. It is based on the fact that in all known cases so far dynein does not transport cargoes without dynactin, and, therefore, potential adaptors are unlikely to bind to the affinity column very efficiently. They compare pull-downs in the presence and absence of dynactin paying specific attention to proteins that bind in the presence of both dynein and dynactin but not dynein alone.

Among the proteins that have been identified by this assay is Heatr5B, the protein known to associate with AP1 clathrin adaptor. Functional characterization of the protein can be divided into two parts, work with mammalian Heatr5B in tissue culture cells and analysis of its function in Drosophila.

In my opinion, the main strength of this work is in the development and use of the original assay for adapter identification. As I already indicated, this is a biologically very important problem for cytoplasmic dynein. Another important strength of the paper is the extension of the work to Drosophila. Demonstration of the fact that Heatr5B is an essential gene, and that the product of this gene is involved in dynein-dependent trafficking in fly embryos makes the results significantly more important.

I do not think there are many problems with the results in this manuscript. Generally speaking, the data on biochemical interactions are not as strong as I would like them to be. This is explained mainly by the fact that the authors do not have an expressed

recombinant Heatr5B that they can use in biochemical experiments, and they limit their biochemistry by pulling down the protein from cell extracts. This creates one of the few experimental problems with the paper. The authors claim that dynein and dynactin do not compete for Heatr5B binding, and therefore they can bind to both components of the complex at the same time. Unfortunately, I do not think that this claim is justified because concentrations of dynein and dynactin in their pull-down assay are much higher than the concentration of GFP-HEATR5B, and likely that HEATR5B does not saturate the binding sites on the motor complex. Therefore, it is unclear whether dynein and dynactin compete for Heatr5B binding. In any case, the conclusion about the competition cannot be seriously made without analysis of saturation curves.

My second concern with this paper is the quality of imaging in mammalian cells. Unfortunately, not much can be done with live cell imaging because GFP-HEATR5B is expressed in cells at a low level (see, for example, Fig. 3A). However, in fixed cells GFP-HEATR5B signal could be easily amplified using anti-GFP antibodies.

A minor problem with movie presentations is that the authors should include both a timer and a scale bar for all their live cell sequences, especially because the movies are looped. The authors did it for Movie 5, and they should do it for the rest of their live cell sequences.

In my opinion, the main novelty of this paper is in its pull-down assay, I would like to have it discussed more extensively. The authors state that they "were particularly drawn to Heatr5B". Is there an objective reason for this choice? If so, it should be specified. Furthermore, I would like to see the authors discuss the other hits. Their list of hits includes a large number of ribosomal proteins. Do ribosomes really interact with dynein? Can the authors speculate on the number of true hits? Finally, it is likely that dynein interacts with some of the cargoes only transiently. How can the assay be modified to capture these transient interactions?

2. Significance:

Significance (Required)

The bottom line is very clear. For me, it is an excellent technical paper with biological results that clearly demonstrate the validity of the technique. As such, it can and should be published.

3. How much time do you estimate the authors will need to complete the suggested revisions:

Estimated time to Complete Revisions (Required)

(Decision Recommendation)

Less than 1 month

4. *Review Commons* values the work of reviewers and encourages them to get credit for their work. Select 'Yes' below to register your reviewing activity at Web of Science Reviewer Recognition Service (formerly Publons); note that the content of your review will not be visible on Web of Science.

Web of Science Reviewer Recognition

Yes

Review #2

1. Evidence, reproducibility and clarity:

Evidence, reproducibility and clarity (Required)

Major points:

As a view of non-expert of light microscopy cellular imaging, some confocal images are difficult to accept as proofs of their conclusion that mutation to decrease HEAT5B/AP1 interaction results in diffusion from perinucleolar surface. For example, fluorescent signals in Control of Fig.4A seem more diffused than HR5B KO, which have fluorescence clearly localized on the surfaces of nuclei. Can they have explanation how it ends up with their statistical analysis in Fig.4E?

When they mention statistically more distance between target molecules and the perinuleolar surface, are dynein/dynactin connected to AP1 via HEAT5B stalled on the microtubule before reaching the minus end, or dissociate from the microtubule? Clarifying this will improve impact of this work. If the current data is not enough to

answer, this reviewer will propose another confocal microscopy with also tubulin labeled. With this, the location of HEAT5B, AP1 etc. with respect to both nuclei and microtubule cytoskeleton will be clarified.
In Line168-169, they concluded AP1gamma associated with TGN rarely overlapped with HEATR5B, based on Fig.3A (where HR5B and AP1 seem overlapped in HeLa cells), Fig.S2A (where AP1gamma and TGN46 seem overlapped in U2OS cells) and Fig.S2B (where HR5B and TGN46 are not overlapped in HeLa cells). Is Fig.3A not contradictory to their conclusion (AP1gamma and HEATR5B not overlapped)? Why did they not directly check the overlap between AP1gamma and HR5B in the same cell in U2OS cells?

Minor points:

Line100-105 and Fig.1EF are not clear. Is it correct that proteins in red bold letters and in blue letters in Fig.1EF are 28 proteins enriched on the dynactin tail? Do authors have any idea why the "dynactin-stimulated" ones (in blue) are localized at left end of this group (relatively less significance of dynactin tail binding, if this reviewer understands correctly)?

Fig.S7: More explanation how to conclude that HR5B KO is dimmer than Ctrl based on this plot would be helpful.

2. Significance:

Significance (Required)

In this work, Madan and colleagues studied dynein adaptor proteins, which are stimulated by dynactin, using proteomics, fluorescent microscopy, live cell imaging techniques for U2OS and fly embryo cells. They especially focused on HEATR5B and proved its role to bind AP1 membrane associate protein for intracellular transport. They first conducted proteomic studies and presented novel lists of dynein-associated proteins and proteins stimulated by dynactin. Among them they decided to prioritize HEATR5B protein (it would be interesting to know their motivation to choose this protein) and carried on fluorescent microscopy studies to characterize roles of HEATR5B in microtubule-based motility. Their approach using U2OS cells is to correlate distribution of HEATR5B and such proteins as AP1gamma, TGN46, RAB11A, which they expect interaction with HEATR5B, between WT and mutants. They remarkably demonstrated distance from perinucleolar membrane is heavily influenced by defect of adaptor function of HEATR5B, by fluorescent microscopy and statistical analysis. Next they made HEATR5B depleted Drosophila embryo by CRSPR-CAS9. They proved its influence on AP1 trafficking to Golgi, which is

another novel finding of this study, consistent with the case of U2OS cells.

In general the whole study proved importance of HEATR5 proteins on AP1 trafficking. Many data are presented in convincing way and carefully statistically analyzed. This work will attract attention of wide audience from the field of cytoskeleton, motor proteins and membrane trafficking. After addressing a few points, the manuscript will be ready for publication.

3. How much time do you estimate the authors will need to complete the suggested revisions:

Estimated time to Complete Revisions (Required)

(Decision Recommendation)

Between 1 and 3 months

4. *Review Commons* values the work of reviewers and encourages them to get credit for their work. Select 'Yes' below to register your reviewing activity at Web of Science Reviewer Recognition Service (formerly Publons); note that the content of your review will not be visible on Web of Science.

Web of Science Reviewer Recognition

Yes

Review #3

1. Evidence, reproducibility and clarity:

Evidence, reproducibility and clarity (Required)

Summary:

The goal of the authors is to identify dynein regulators which control how dynein and dynactin complexes orchestrate trafficking of diverse cargoes. To do so, the authors have performed a well thought proteomic screen for novel interacting proteins of the dynein tail potentially enhanced by dynactin. These pull-down experiments identified about 50 new dynein tail-interacting proteins, many of which were enhanced by dynactin.

The authors focused on one candidate, HEATR5B, because it was robustly isolated from the screens and its association with the dynein tail was stimulated by exogenous dynactin.
HEATR5B is known to interact with AP1 complex, as adaptors that orchestrate cargo loading of clathrin-coated vesicles from intracellular membranes.

The authors further show that HEATR5B complexes with endogenous dynactin and dynein as reveal by immuno precipitation from human cells extracts and can interact with both complexes directly. Then by using Hela cell line stably expressing GFP-HEATR5B, they show that HEATR5B is selectively enriched on the AP1 structure, some of which can be subjected to long-distance transport. They provide evidences that a large proportion of the HEATR5B-positive structures are associated with endosomal recycling membranes, as revealed by colocalization with RAB11A. They further show that the HEATR5B/ AP1 and HEATR5B/ RAB11 membrane structures show similar dynamics, indicating that HEATR5B associate with endosomal membranes that are capable of directed movement. SiRNA depletion of DYNC1H1 reveals that dynein promotes retrograde trafficking of AP-1 associated endosomal membranes.

The authors then investigate the contribution of HEATR5B to AP1-associated membrane trafficking by CRIPR/cas9-mediated mutagenesis in human U2OS cells that disrupt HEATR5B protein expression. They provide evidences that in HEATR5B mutant cells, there is a reduction in the amount of AP1 signal associated with RAB11A-positive structures indicating that disrupting HEATR5B reduces the association of AP1with endosomal membranes. This indicates that HEATR5B promotes AP1 recruitment to endosomal membranes.
HEATR5B overexpression in U2OS cells increased perinuclear clustering of Rab11A/AP1/dynactin-associated membrane, suggesting that HEATR5B can stimulate retrograde trafficking of AP1-associated endosomal membranes by dynein-dynactin.

To assess the importance of HEATR5B function at the organismal level, as well as in polarized cell type the authors investigate its function in Drosophila in which there is a single HEATR5B homologue (Heatr5). They generated via crisper an Heatr5 mutant

strain. Heatr5 homozygous mutants are zygotic lethal that died in second larval instar stage. They further provide evidence by investigating nos-cas9 gRNA-Hr51+2 mothers, that Heatr5 plays maternal function essential for embryogenesis. They further show that in early embryos from nos-cas9 gRNA-Hr5 females AP1 puncta are strongly reduced and dimmer.

Next, to understand the effect of Heatr5 disruption on AP1-based trafficking in Drosophila, they used the syncytial blastoderm embryo in which the microtubule cytoskeleton is highly polarized with less apically nucleated ends above the nuclei and more basally extended ends. In this system, the activity of minus end-directed motor, such as dynein, and minus end-directed motor, such as kinesin, can be distinguished by the direction of cargo movement.

By injecting AP1 antibodies into wild-type and Heatr5 mutant embryos, they provide evidence that AP1 undergoes net apical transport in the Drosophila embryo and that this process is strongly promoted by Heatr5. They further show that this process is microtubule and dynein dependent and that Heatr5 selectively promotes dynein-mediated transport of AP1 structures in the embryo.
They then show that Heatr5-dependent AP1 trafficking pathways in the embryo involves the endosomal and Golgi membranes and that Heatr5 is also required for Golgi organization.

Major Comments

This study is very comprehensive and multi-scale. It ranges from the identification of a dynein motor adaptor for membrane trafficking by a proteomic screen, to its functional characterization in human cells and then during development with Drosophila embryo as model organism.
The data are of high quality and are supported by very convincing quantitative analyses. The results are conclusive and the experiments have been carried out and presented in a very constructive way. This combination makes the manuscript very interesting.

Minor comments

HEATR5B overexpression in U2OS cells increased perinuclear clustering of Rab11A/AP1/dynactin-associated membrane. To which compartment are these vesicles directed and associated, the Golgi apparatus? Could the authors show which compartment it is?

2. Significance:

Significance (Required)

This study is important in two aspects. Firstly, it has identified HEATR5B as a new adaptor of the dynein motor for intracellular membrane trafficking. It is important to mention that this motor is involved in many transport processes and it is still unclear how a single motor orchestrates the traffic of so many cargoes.
Second, this work shed new light on the retrograde trafficking from endosomal material to the Golgi apparatus, in particular with HEATR5B, a known interactor of the AP1 clathrin adapter complex. This study highlights a role of HEATR5B in a novel dynein-based process for retrograde trafficking of AP1-associated endosomal vesicle to the Golgi apparatus. It also indicates that HEATR5B promotes association of AP1 with endosomal membrane in a dynein-independent manner.

This work is particularly important for the cell biology field.

3. How much time do you estimate the authors will need to complete the suggested revisions:

Estimated time to Complete Revisions (Required)

(Decision Recommendation)

Between 1 and 3 months

4. *Review Commons* values the work of reviewers and encourages them to get credit for their work. Select 'Yes' below to register your reviewing activity at Web of Science Reviewer Recognition Service (formerly Publons); note that the content of your review will not be visible on Web of Science.

Web of Science Reviewer Recognition

Yes

Revision Plan

Manuscript number: #RC-2023-01926
Corresponding author(s): Simon Bullock

1. General Statements

This section is optional. Insert here any general statements you wish to make about the goal of the study or about the reviews.

Please see cover letter for statements about the goal of the study. Please note that most of the reviewers' introductory comments, as well as their significance statements, are not included in the point-by-point replies in the plan.

2. Description of the planned revisions

Insert here a point-by-point reply that explains what revisions, additional experimentations and analyses are planned to address the points raised by the referees.

Reviewer 1:

A minor problem with movie presentations is that the authors should include both a timer and a scale bar for all their live cell sequences, especially because the movies are looped. The authors did it for Movie 5, and they should do it for the rest of their live cell sequences.

Although information on the duration of movies (including loops) was included in the legends, we agree that it would be helpful to incorporate timers and scale bars in the movies. We have not been able to include this change in the preliminary revision as we have to co-ordinate with our visual aids team to reapply labels and arrows to the edited movies. We will upload the edited movies in the full revision.

Reviewer 3:

Major Comments

This study is very comprehensive and multi-scale. It ranges from the identification of a dynein motor adaptor for membrane trafficking by a proteomic screen, to its functional characterization in human cells and then during development with Drosophila embryo as model organism.
The data are of high quality and are supported by very convincing quantitative analyses. The results are conclusive and the experiments have been carried out and presented in a very constructive way. This combination makes the manuscript very interesting.

Minor comments

HEATR5B overexpression in U2OS cells increased perinuclear clustering of Rab11A/AP1/dynactin-associated membrane. To which compartment are these vesicles directed and associated, the Golgi apparatus? Could the authors show which compartment it is?

We plan to perform new experiments to address this minor comment. However, given their location near the microtubule organising centre, it is likely that the relocalised membranes will be in the vicinity of the Golgi apparatus.

3. Description of the revisions that have already been incorporated in the transferred manuscript

Please insert a point-by-point reply describing the revisions that were already carried out and included in the transferred manuscript. If no revisions have been carried out yet, please leave this section empty.

Reviewer 1:

In my opinion, the main strength of this work is in the development and use of the original assay for adapter identification. As I already indicated, this is a biologically very important problem for cytoplasmic dynein. Another important strength of the paper is the extension of the work to Drosophila. Demonstration of the fact that Heatr5B is an essential gene, and that the product of this gene is involved in dynein-dependent trafficking in fly embryos makes the results significantly more important.

I do not think there are many problems with the results in this manuscript. Generally speaking, the data on biochemical interactions are not as strong as I would like them to be. This is explained mainly by the fact that the authors do not have an expressed recombinant Heatr5B that they can use in biochemical experiments, and they limit their biochemistry by pulling down the protein from cell extracts.

Whilst we are very grateful to the reviewer for their thorough evaluation of our work, we do not understand this particular comment. We did include data with recombinant HEATR5B showing binding in vitro to purified dynein and dynactin complexes. The results are shown in Fig. 2B. We have now made it clearer (from line 153 of the preliminarily revised manuscript) that these experiments used recombinant HEATR5B. We hope in the future to determine the biochemical and structural basis of HEATR5B's interaction with dynein and dynactin but feel that this goes well beyond the scope of this initial study (which already covers a lot of ground), especially as we have not yet found a way to express HEATR5B fragments (line 151).

This creates one of the few experimental problems with the paper. The authors claim that dynein and dynactin do not compete for Heatr5B binding, and therefore they can bind to both components of the complex at the same time. Unfortunately, I do not think that this claim is justified because concentrations of dynein and dynactin in their pull-down assay are much higher than the concentration of GFP-HEATR5B, and likely that HEATR5B does not saturate the binding sites on the motor complex. Therefore, it is unclear whether dynein and dynactin compete for Heatr5B binding. In any case, the conclusion about the competition cannot be seriously made without analysis of saturation curves.

The purified dynein and dynactin were not in excess to recombinant HEATR5B in this assay (80 pmol HEATR5B, 20 pmol dynein tail and 10 pmol dynactin). Nonetheless, the reviewer makes a very good point that we cannot draw strong conclusions about competition unless we generate saturation curves. We have therefore toned down the interpretation of this experiment and included the caveat raised by the reviewer (from line 157):

'Compatible with this notion, we did not observe competition between the purified dynein tail and dynactin for HEATR5B binding in our in vitro binding assay when both complexes were added simultaneously to the beads (Figure 2B). However, we cannot rule out the possibility that a competitive interaction was masked by binding sites on one of the components not being saturated. Nonetheless, we can conclude from this set of experiments that HEATR5B complexes with endogenous dynactin and dynein in cell extracts and can interact with both complexes directly.'

Please note that this was only a minor point in our manuscript.

My second concern with this paper is the quality of imaging in mammalian cells. Unfortunately, not much can be done with live cell imaging because GFP-HEATR5B is expressed in cells at a low level (see, for example, Fig. 3A). However, in fixed cells GFP-HEATR5B signal could be easily amplified using anti-GFP antibodies.

The fixed cell images of GFP-HEATR5B cells are stained with anti-GFP antibodies and are the result of extensive optimisation of staining and imaging conditions. Due to its low expression and presence in both cytoplasm and membrane-bound pools, the signal for GFP-HEATR5B is not as striking as, for example, RAB11A and AP1γ. Nonetheless, the punctate signals are sufficiently strong to confidently evaluate co-localisation with membrane markers. We have now added to the relevant legends that the GFP signal is obtained via GFP antibody staining. Please note that the association of GFP-HEATR5B with AP1γ (Fig. 3A, B) was also confirmed by immunoprecipitation (Fig. 2A).

A minor problem with movie presentations is that the authors should include both a timer and a scale bar for all their live cell sequences, especially because the movies are looped. The authors did it for Movie 5, and they should do it for the rest of their live cell sequences.

As described above, we intend to address this point in the full revision.

In my opinion, the main novelty of this paper is in its pull-down assay, I would like to have it discussed more extensively. The authors state that they "were particularly drawn to Heatr5B". Is there an objective reason for this choice? If so, it should be specified.

We included our two reasons for focusing on HEATR5B in the previous submission, namely that it was the only protein to be enriched on the tail by dynactin in both the N- and C-terminal tethering configuration and that a previous study found it was one of a number of proteins present on dynactin-associated vesicles. We have modified the language in this section (which starts on line 120 of the preliminary revisions) by using the connective ‘because’. This change makes it clearer that there were objective reasons for focusing on HEATR5B in the first instance.

Furthermore, I would like to see the authors discuss the other hits. Their list of hits includes a large number of ribosomal proteins. Do ribosomes really interact with dynein? Can the authors speculate on the number of true hits? Finally, it is likely that dynein interacts with some of the cargoes only transiently. How can the assay be modified to capture these transient interactions?

This is another very good suggestion. As requested, we have added a comment to the Discussion (from line 441) about how transient interactions might be captured using a variation of our strategy. Based on an ongoing project of a PhD student in the lab, we believe the interactions between dynein and ribosomes are functional but we cannot add the supporting data to the HEATR5B manuscript as this would jeopardise the student’s chances of publishing her data well. Nonetheless, we have now added a comment in the Discussion (from line 435) about the capture of ribosomes and other RNA-associated proteins in our screen, as well as the potential significance of this observation. We have also highlighted in this section another dynactin-stimulated hit, Wdr91, which we are following up. We also discussed the STRIPAK complex, which warrants further study, in the Results (line 106). We do not have space to discuss other hits but their identities are listed in Tables S1 and S2 together with a summary of their known functions for easy reference.

Reviewer 2:

Major points:

As a view of non-expert of light microscopy cellular imaging, some confocal images are difficult to accept as proofs of their conclusion that mutation to decrease HEAT5B/AP1 interaction results in diffusion from perinucleolar surface. For example, fluorescent signals in Control of Fig.4A seem more diffused than HR5B KO, which have fluorescence clearly localized on the surfaces of nuclei. Can they have explanation how it ends up with their statistical analysis in Fig.4E?

Fig. 4A is representative of what we typically see in mutant cells, with dispersion of the dimmer AP1γ signal in the cytoplasm and less disturbed localisation of the brighter AP1γ signal at the TGN (see Fig. S6B for quantification of AP1γ signal at the TGN in control and mutant cells). We should have made it clear in the Results that the unbiased image analysis pipeline used to produce Fig. 4E detects the total AP1γ signal not just bright signals (this feature of the pipeline is important given the differences in fluorescent intensity of puncta in the two genotypes). We have now clarified this issue in the Results (line 257) and the Fig. 4E legend. We have also added arrowheads to Fig. 4A to highlight the dispersed dim signal in the mutant cells. We thank the reviewer for leading us to improve the description of this experiment.

In Line168-169, they concluded AP1gamma associated with TGN rarely overlapped with HEATR5B, based on Fig.3A (where HR5B and AP1 seem overlapped in HeLa cells), Fig.S2A (where AP1gamma and TGN46 seem overlapped in U2OS cells) and Fig.S2B (where HR5B and TGN46 are not overlapped in HeLa cells). Is Fig.3A not contradictory to their conclusion (AP1gamma and HEATR5B not overlapped)? Why did they not directly check the overlap between AP1gamma and HR5B in the same cell in U2OS cells?

We don't understand why our co-localisation data might be contradictory to our conclusions. Fig. 3A, together with the associated insets and quantification in Fig. 3B, show overlap of HEATR5B with AP1γ puncta in the cytoplasm of HeLa cells but not the AP1γ that is strong enriched in the perinuclear region, as we stated in the results. Absence of enrichment of HEATR5B with the TGN is additionally shown in Fig. S2B. These observations are commented on further in the Discussion (from line 517). We do, however, agree with the reviewer that is was not ideal that we did not show AP1 and TGN association in a HeLa cell (even though it has been documented in the literature). We have now corrected this oversight by showing HeLa cell data in Fig. S2A. We could not check the overlap of AP1 and HEATR5B in U2OS cells as we do not have a GFP-HEATR5B stable U2OS cell line.

Minor points:

Line100-105 and Fig.1EF are not clear. Is it correct that proteins in red bold letters and in blue letters in Fig.1EF are 28 proteins enriched on the dynactin tail?

We should have been clearer here and thank the reviewer for spotting this. We have modified the figure call outs in the text to include the labelling scheme, which we think helps significantly. We have also clarified the labelling system in the legend. To summarise, bold labelling indicates interactors of the dynein tail that are not core components of the dynein-dynactin machinery (such proteins are labelled in non-bold and italics); the blue bold text shows those 'none core' interactors that were only enriched on the dynein tail when exogenous dynactin was spiked into the lysates.

Do authors have any idea why the "dynactin-stimulated" ones (in blue) are localized at left end of this group (relatively less significance of dynactin tail binding, if this reviewer understands correctly)?

This question appears to indicate some confusion about whether we are capturing the dynein tail or dynactin. We believe the changes made in response to the previous comment about the labelling scheme should help clear this up. Being positioned to the left of this grouping shows a lower degree on enrichment vs the control (although still greater than 10 fold), rather than a difference in statistical significance. The observation that core dynein-dynactin subunits are more enriched on the dynein tail indicates that these interactions are the most stable or the most frequent.

Fig.S7: More explanation how to conclude that HR5B KO is dimmer than Ctrl based on this plot would be helpful.

We have added a line to the legend to Figure S7C to clarify this matter.

4. Description of analyses that authors prefer not to carry out

Please include a point-by-point response explaining why some of the requested data or additional analyses might not be necessary or cannot be provided within the scope of a revision. This can be due to time or resource limitations or in case of disagreement about the necessity of such additional data given the scope of the study. Please leave empty if not applicable.

Reviewer 2:

When they mention statistically more distance between target molecules and the perinuleolar surface, are dynein/dynactin connected to AP1 via HEAT5B stalled on the microtubule before reaching the minus end, or dissociate from the microtubule? Clarifying this will improve impact of this work. If the current data is not enough to answer, this reviewer will propose another confocal microscopy with also tubulin labeled. With this, the location of HEAT5B, AP1 etc. with respect to both nuclei and microtubule cytoskeleton will be clarified.

We would love to know the answer to the question of whether HEATR5B disruption reduces the association of AP1γ with microtubules. We have looked into co-localisation of microtubules and dynein's cargoes previously using advanced light microscopy and found that it is not possible to draw conclusions about meaningful versus coincidental associations because of the density of the microtubule network. In the case of our current study, this approach would be further confounded by the difference in size in fluorescent AP1 puncta in control and HEATR5B mutant cells. We have also in the past attempted to purify recycling endosomal membranes from cells to determine how loss of HEATR5B influences dynein-dynactin association. However, even after extensive efforts we could not reproduce selective purification of recycling endosomes using the published protocol, or indeed variants of it. What is more, we find in general that there

Review COMMONS

is rapid dissociation of motors during purification of membranes from cells, which would confound our results even if we could purify the recycling compartment. We therefore feel that the only way to address the question of how HEATR5B modulates dynein function at the molecular level is to reconstitute the transport machinery with pure proteins (including the as-of-yet unidentified activating adaptor) and microtubules in vitro, which is beyond the scope of this study. We have discussed the future aim of in vitro reconstitution to dissect mechanism in the Discussion (from line 494).

1st Editorial Decision **11th May 2023**

Dear Dr. Bullock,

Thank you for submitting your manuscript entitled 'HEATR5B associates with dynein-dynactin and selectively promotes motility of AP1-bound endosomal membranes' (EMBOJ-2023-114473) via Review Commons for consideration by the EMBO Journal. Your work initially uses a dynactin-induced difference in binding affinity between dynein and its interactors, then proceeds to identify HEATR5B as a protein that is required for the motility of AP1-containing vesicles. Your team goes on to investigate the role HEATR5B plays in the recruitment of AP1 to the endosome.

Given the referees' positive recommendations, I would like to invite you to submit a revised version of the manuscript, addressing the comments as you outline in the accompanying revision plan. I should add that it is EMBO Journal policy to allow only a single round of revision, and acceptance of your manuscript will therefore depend on the completeness of your responses in this revised version.

When finalising your letter of response to the referees' comments, please bear in mind that this will form part of the Review Process File, and will therefore be available online to the community. For more details on our Transparent Editorial Process, please visit our website: https://www.embopress.org/page/journal/14602075/authorguide#transparentprocess

We generally allow three months as standard revision time. As a matter of policy, competing manuscripts published during this period will not negatively impact on our assessment of the conceptual advance presented by your study. However, we request that you contact the editor as soon as possible upon publication of any related work, to discuss how to proceed. Should you foresee a problem in meeting this three-month deadline, please let us know in advance and we may be able to grant an extension.

Thank you for the opportunity to consider your work for publication. I look forward to your revision.

Yours sincerely,

William Teale

William Teale, PhD
Editor
The EMBO Journal
w.teale@embojournal.org

When submitting your revised manuscript, please carefully review the instructions below and include the following items:

1) a .docx formatted version of the manuscript text (including legends for main figures, EV figures and tables). Please make sure that the changes are highlighted to be clearly visible.

2) individual production quality figure files as .eps, .tif, .jpg (one file per figure).

3) a .docx formatted letter INCLUDING the reviewers' reports and your detailed point-by-point response to their comments. As part of the EMBO Press transparent editorial process, the point-by-point response is part of the Review Process File (RPF), which will be published alongside your paper.

4) a complete author checklist, which you can download from our author guidelines (https://wol-prod-cdn.literatumonline.com/pb-assets/embo-site/Author Checklist%20-%20EMBO%20J-1561436015657.xlsx). Please insert information in the checklist that is also reflected in the manuscript. The completed author checklist will also be part of the RPF.

5) Please note that all corresponding authors are required to supply an ORCID ID for their name upon submission of a revised manuscript.

6) We require a 'Data Availability' section after the Materials and Methods. Before submitting your revision, primary datasets produced in this study need to be deposited in an appropriate public database, and the accession numbers and database listed under 'Data Availability'. Please remember to provide a reviewer password if the datasets are not yet public (see https://www.embopress.org/page/journal/14602075/authorguide#datadeposition). If no data deposition in external databases is needed for this paper, please then state in this section: This study includes no data deposited in external repositories. Note that the Data Availability Section is restricted to new primary data that are part of this study.

Note - All links should resolve to a page where the data can be accessed.

7) When assembling figures, please refer to our figure preparation guideline in order to ensure proper formatting and readability in print as well as on screen:
http://bit.ly/EMBOPressFigurePreparationGuideline

Please remember: Digital image enhancement is acceptable practice, as long as it accurately represents the original data and conforms to community standards. If a figure has been subjected to significant electronic manipulation, this must be noted in the figure legend or in the 'Materials and Methods' section. The editors reserve the right to request original versions of figures and the original images that were used to assemble the figure.

8) For data quantification: please specify the name of the statistical test used to generate error bars and P values, the number (n) of independent experiments (specify technical or biological replicates) underlying each data point and the test used to calculate p-values in each figure legend. The figure legends should contain a basic description of n, P and the test applied. Graphs must include a description of the bars and the error bars (s.d., s.e.m.).

9) At EMBO Press we ask authors to provide source data for the main manuscript figures. Our source data coordinator will contact you to discuss which figure panels we would need source data for and will also provide you with helpful tips on how to upload and organize the files.
Briefly, numerical data can be provided as individual .xls or .csv files (including a tab describing the data). For 'blots' or microscopy, uncropped images should be submitted (using a zip archive or a single pdf per main figure if multiple images need to be supplied for one panel). Additional information on source data and instruction on how to label the files are available at .

10) We replaced Supplementary Information with Expanded View (EV) Figures and Tables that are collapsible/expandable online (see examples in https://www.embopress.org/doi/10.15252/embj.201695874). A maximum of 5 EV Figures can be typeset. EV Figures should be cited as 'Figure EV1, Figure EV2" etc. in the text and their respective legends should be included in the main text after the legends of regular figures.

- For the figures that you do NOT wish to display as Expanded View figures, they should be bundled together with their legends in a single PDF file called *Appendix*, which should start with a short Table of Content. Appendix figures should be referred to in the main text as: "Appendix Figure S1, Appendix Figure S2" etc. See detailed instructions regarding expanded view here: .

- Additional Tables/Datasets should be labelled and referred to as Table EV1, Dataset EV1, etc. Legends have to be provided in a separate tab in case of .xls files. Alternatively, the legend can be supplied as a separate text file (README) and zipped together with the Table/Dataset file.

11) Our journal encourages inclusion of *data citations in the reference list* to directly cite datasets that were re-used and obtained from public databases. Data citations in the article text are distinct from normal bibliographical citations and should directly link to the database records from which the data can be accessed. In the main text, data citations are formatted as follows: "Data ref: Smith et al, 2001" or "Data ref: NCBI Sequence Read Archive PRJNA342805, 2017". In the Reference list, data citations must be labeled with "[DATASET]". A data reference must provide the database name, accession number/identifiers and a resolvable link to the landing page from which the data can be accessed at the end of the reference. Further instructions are available at .

Further instructions for preparing your revised manuscript:

Please make sure you upload a letter of response to the referees' comments together with the revised manuscript.

Please also check that the title and abstract of the manuscript are brief, yet explicit, even to non-specialists.

When assembling figures, please refer to our figure preparation guideline in order to ensure proper formatting and readability in print as well as on screen:
https://bit.ly/EMBOPressFigurePreparationGuideline
See also guidelines for figure legends: https://www.embopress.org/page/journal/14602075/authorguide#figureformat

At EMBO Press we ask authors to provide source data for the main manuscript figures. Our source data coordinator will contact you to discuss which figure panels we would need source data for and will also provide you with helpful tips on how to upload and organize the files.

IMPORTANT: When you send the revision we will require
- a point-by-point response to the referees' comments, with a detailed description of the changes made (as a word file).
- a word file of the manuscript text.
- individual production quality figure files (one file per figure)
- a complete author checklist, which you can download from our author guidelines

(https://www.embopress.org/page/journal/14602075/authorguide).
- Expanded View files (replacing Supplementary Information)
Please see out instructions to authors
https://www.embopress.org/page/journal/14602075/authorguide#expandedview

Please remember: Digital image enhancement is acceptable practice, as long as it accurately represents the original data and conforms to community standards. If a figure has been subjected to significant electronic manipulation, this must be noted in the figure legend or in the 'Materials and Methods' section. The editors reserve the right to request original versions of figures and the original images that were used to assemble the figure.

Further information is available in our Guide For Authors: https://www.embopress.org/page/journal/14602075/authorguide

We realize that it is difficult to revise to a specific deadline. In the interest of protecting the conceptual advance provided by the work, we recommend a revision within 3 months (9th Aug 2023). Please discuss the revision progress ahead of this time with the editor if you require more time to complete the revisions. Use the link below to submit your revision:

https://emboj.msubmit.net/cgi-bin/main.plex

We are very grateful to the reviewers for their thorough evaluation of the manuscript and for their positive and constructive feedback. We include below a point-by-point response to their comments, which is based on a revision plan that has been approved by the Editor. Please note that the line numbers in the response refer to the Word document; the PDF generated by the Manuscript Submission System appears to have slightly different line numbers.

Reviewer #1 (Evidence, reproducibility and clarity (Required)):

This work is the first systematic attempt to identify and characterize a diverse set of adapters that attach cytoplasmic dynein to its different cargoes and thus activate the motor. It is an important work because in animal cells dynein is the only efficient motor that can perform processive transport toward the minus ends of microtubules, and therefore the specificity of transport for multiple cargoes along microtubules is determined by these adapters.

The authors use the recombinant tail of dynein for pulling down interacting proteins from the cell extract. This is a straightforward approach, but its main problem is the large number of non-specific proteins that bind to the column. To solve the problem, the authors use a very smart approach. It is based on the fact that in all known cases so far dynein does not transport cargoes without dynactin, and, therefore, potential adaptors are unlikely to bind to the affinity column very efficiently. They compare pull-downs in the presence and absence of dynactin paying specific attention to proteins that bind in the presence of both dynein and dynactin but not dynein alone.

Among the proteins that have been identified by this assay is Heatr5B, the protein known to associate with AP1 clathrin adaptor. Functional characterization of the protein can be divided into two parts, work with mammalian Heatr5B in tissue culture cells and analysis of its function in Drosophila.

In my opinion, the main strength of this work is in the development and use of the original assay for adapter identification. As I already indicated, this is a biologically very important problem for cytoplasmic dynein. Another important strength of the paper is the extension of the work to Drosophila. Demonstration of the fact that Heatr5B is an essential gene, and that the product of this gene is involved in dynein-dependent trafficking in fly embryos makes the results significantly more important.

I do not think there are many problems with the results in this manuscript. Generally speaking, the data on biochemical interactions are not as strong as I would like them to be. This is explained mainly by the fact that the authors do not have an expressed recombinant Heatr5B that they can use in biochemical experiments, and they limit their biochemistry by pulling down the protein from cell extracts.

Whilst we are very grateful to the reviewer for their thorough evaluation of our work, we do not understand this particular comment. We did include data with recombinant HEATR5B showing binding to purified dynein and dynactin complexes in vitro. The results are shown in Fig 2B. We have now made it clearer (on lines 209 and 210 of the revised manuscript) that these experiments used recombinant HEATR5B. We hope in the future to determine the biochemical and structural basis of HEATR5B's interaction with dynein and dynactin but feel that this goes

well beyond the scope of this initial study (which already covers a lot of ground), especially as we have not yet found a way to express HEATR5B fragments (line 207).

This creates one of the few experimental problems with the paper. The authors claim that dynein and dynactin do not compete for Heatr5B binding, and therefore they can bind to both components of the complex at the same time. Unfortunately, I do not think that this claim is justified because concentrations of dynein and dynactin in their pull-down assay are much higher than the concentration of GFP-HEATR5B, and likely that HEATR5B does not saturate the binding sites on the motor complex. Therefore, it is unclear whether dynein and dynactin compete for Heatr5B binding. In any case, the conclusion about the competition cannot be seriously made without analysis of saturation curves.

The purified dynein and dynactin were not in excess to recombinant HEATR5B in this assay (80 pmol HEATR5B, 20 pmol dynein tail and 10 pmol dynactin). Nonetheless, the reviewer makes a very good point that we cannot draw strong conclusions about competition without further experiments (e.g. saturation curves). We have therefore toned down the interpretation of this experiment and included the specific caveat raised by the reviewer (from line 213):

'*Compatible with this notion, we did not observe competition between the purified dynein tail and dynactin for HEATR5B binding in our in vitro binding assay when both complexes were added simultaneously to the beads (Fig 2B). However, we cannot rule out the possibility that a competitive interaction was masked by binding sites on one of the components not being saturated. Nonetheless, we can conclude from this set of experiments that HEATR5B complexes with endogenous dynactin and dynein in cell extracts and can interact with both complexes directly.*'

Please note that this was only a minor point in our manuscript.

My second concern with this paper is the quality of imaging in mammalian cells. Unfortunately, not much can be done with live cell imaging because GFP-HEATR5B is expressed in cells at a low level (see, for example, Fig. 3A). However, in fixed cells GFP-HEATR5B signal could be easily amplified using anti-GFP antibodies.

The fixed cell images of stable GFP-HEATR5B cells were generated using anti-GFP antibodies and are the result of extensive optimisation of staining and imaging conditions. Due to its low expression and presence in both cytoplasm and membrane-bound pools, the signal for GFP-HEATR5B is not as striking as, for example, RAB11A and AP1γ. Nonetheless, the punctate signals are sufficiently strong to confidently evaluate co-localisation with membrane markers. We have now added to the relevant legends that the GFP signal was amplified via GFP antibody staining.

A minor problem with movie presentations is that the authors should include both a timer and a scale bar for all their live cell sequences, especially because the movies are looped. The authors did it for Movie 5, and they should do it for the rest of their live cell sequences.

We agree that it would be helpful to incorporate timers and scale bars in all movies. This has now been done.

In my opinion, the main novelty of this paper is in its pull-down assay, I would like to have it discussed more extensively. The authors state that they "were particularly drawn to Heatr5B". Is there an objective reason for this choice? If so, it should be specified.

We included our two reasons for focusing on HEATR5B in the previous submission, namely that it was the only protein enriched by dynactin on the tail in both the N- and C-terminal tethered configuration and that a previous study found it was one of a number of proteins present on dynactin-associated vesicles. We have modified the language in this section (which starts on line 176) by using the connective 'because'. We believe this change makes it clear that there were objective reasons for initially concentrating on HEATR5B.

Furthermore, I would like to see the authors discuss the other hits. Their list of hits includes a large number of ribosomal proteins. Do ribosomes really interact with dynein? Can the authors speculate on the number of true hits? Finally, it is likely that dynein interacts with some of the cargoes only transiently. How can the assay be modified to capture these transient interactions?

This is another very good suggestion. As requested, we have added a comment to the Discussion (from line 500) about how transient interactions might be captured using a variation of our strategy. Based on an ongoing project of a PhD student in the lab, we believe the interactions between dynein and ribosomes are functional but we cannot add the extensive supporting data to the HEATR5B manuscript as this would jeopardise the student's chances of publishing her data well. Nonetheless, we have now added a comment in the Discussion (from line 494) about the capture of ribosomes and other RNA-associated proteins in our screen, as well as the potential significance of this observation. We have also highlighted in this section another dynactin-stimulated hit, Wdr91, which we are also following up. We discussed the STRIPAK complex, which also warrants further study, in the Results (from line 162). We do not have space to discuss other hits but their identities are listed in Appendix Tables S1 and S2 together with a summary of their known functions for easy reference.

Reviewer #1 (Significance (Required)):

The bottom line is very clear. For me, it is an excellent technical paper with biological results that clearly demonstrate the validity of the technique. As such, it can and should be published.

Reviewer #2 (Evidence, reproducibility and clarity (Required)):

Major points:

As a view of non-expert of light microscopy cellular imaging, some confocal images are difficult to accept as proofs of their conclusion that mutation to decrease HEAT5B/AP1 interaction results in diffusion from perinucleolar surface. For example, fluorescent signals in Control of Fig.4A seem more diffused than HR5B KO, which have fluorescence clearly localized on the surfaces of nuclei. Can they have explanation how it ends up with their statistical analysis in Fig.4E?

Fig 4A is representative of what we typically see in mutant cells, with dispersion of the dimmer AP1γ signal in the cytoplasm and less disturbed localisation of the brighter AP1γ signal at the

TGN (see Fig EV2B for quantification of AP1γ signal at the TGN in control and mutant cells). We should have made it clear in the Results that the unbiased image analysis pipeline used to produce Fig 4E detects the total AP1γ signal not just bright signals (this feature of the pipeline is important given the differences in fluorescent intensity of puncta in the two genotypes). We have now clarified this matter in the Results (line 314) and the Fig 4E legend. We have also added arrowheads to Fig 4A to highlight the dispersed dim signal in the mutant cells. We thank the reviewer for leading us to improve the description of this experiment**.**

When they mention statistically more distance between target molecules and the perinuleolar surface, are dynein/dynactin connected to AP1 via HEAT5B stalled on the microtubule before reaching the minus end, or dissociate from the microtubule? Clarifying this will improve impact of this work. If the current data is not enough to answer, this reviewer will propose another confocal microscopy with also tubulin labeled. With this, the location of HEAT5B, AP1 etc. with respect to both nuclei and microtubule cytoskeleton will be clarified.

We would love to know the answer to the question of whether HEATR5B disruption reduces the association of AP1γ with microtubules. We have looked into co-localisation of microtubules and dynein's cargoes previously using advanced light microscopy and found that it is not possible to draw conclusions about meaningful versus coincidental associations because of the density of the microtubule network. In the case of our current study, this approach would be further confounded by the difference in apparent size in fluorescent AP1 puncta in control and HEATR5B mutant cells. We have also in the past attempted to purify recycling endosomal membranes from cells to determine how loss of HEATR5B influences dynein-dynactin association. However, even after extensive efforts we could not reproduce selective purification of recycling endosomes using the published protocol, or indeed variants of it. Moreover, we find in general that there is rapid dissociation of motors during purification of membranes from cells, which would confound our results even if we could purify the recycling compartment. We therefore feel that the only way to address the question of how HEATR5B modulates dynein function at the molecular level is to reconstitute the transport machinery with pure proteins (including the as-of-yet unidentified activating adaptor) and microtubules in vitro, which is beyond the scope of this study. We had introduced the value of in vitro reconstitution to dissect mechanism in the Discussion (from line 552).

In Line168-169, they concluded AP1gamma associated with TGN rarely overlapped with HEATR5B, based on Fig.3A (where HR5B and AP1 seem overlapped in HeLa cells), Fig.S2A (where AP1gamma and TGN46 seem overlapped in U2OS cells) and Fig.S2B (where HR5B and TGN46 are not overlapped in HeLa cells). Is Fig.3A not contradictory to their conclusion (AP1gamma and HEATR5B not overlapped)? Why did they not directly check the overlap between AP1gamma and HR5B in the same cell in U2OS cells?

We don't understand why our co-localisation data might be contradictory to our conclusions. Fig 3A, together with the associated insets and quantification in Fig 3B, show overlap of HEATR5B with AP1γ puncta in the cytoplasm of HeLa cells but not with the AP1γ that is strong enriched in the perinuclear TGN, as we stated in the Results. Absence of enrichment of HEATR5B on the TGN was additionally shown in Fig S2B (now Fig EV1B). These observations are commented on further in the Discussion (from line 575). We do, however, agree with the reviewer that we should have shown AP1 and TGN association in a HeLa cell, even though it has been documented in the literature. We have now corrected this oversight

by showing these data in Fig EV1A. We could not check the overlap of AP1 and HEATR5B in U2OS cells as we do not have a GFP-HEATR5B stable U2OS cell line.

Minor points:

Line100-105 and Fig.1EF are not clear. Is it correct that proteins in red bold letters and in blue letters in Fig.1EF are 28 proteins enriched on the dynactin tail? Do authors have any idea why the "dynactin-stimulated" ones (in blue) are localized at left end of this group (relatively less significance of dynactin tail binding, if this reviewer understands correctly)?

We should have been clearer here and thank the reviewer for spotting this. We have modified the figure call outs in the main text to include the labelling scheme, which we think helps significantly. We have also clarified the labelling system in the legend. To summarise, bold labelling indicates interactors of the dynein tail that are not core components of the dynein-dynactin machinery (which are labelled in non-bold and italics); the blue bold text shows those 'none core' interactors that were only enriched on the dynein tail when exogenous dynactin was spiked into the lysates.

Fig.S7: More explanation how to conclude that HR5B KO is dimmer than Ctrl based on this plot would be helpful.

We have added a line to the legend of this panel (now Fig EV3C) to clarify this matter.

Reviewer #2 (Significance (Required)):

In this work, Madan and colleagues studied dynein adaptor proteins, which are stimulated by dynactin, using proteomics, fluorescent microscopy, live cell imaging techniques for U2OS and fly embryo cells. They especially focused on HEATR5B and proved its role to bind AP1 membrane associate protein for intracellular transport.

They first conducted proteomic studies and presented novel lists of dynein-associated proteins and proteins stimulated by dynactin. Among them they decided to prioritize HEATR5B protein (it would be interesting to know their motivation to choose this protein) and carried on fluorescent microscopy studies to characterize roles of HEATR5B in microtubule-based motility. Their approach using U2OS cells is to correlate distribution of HEATR5B and such proteins as AP1gamma, TGN46, RAB11A, which they expect interaction with HEATR5B, between WT and mutants. They remarkably demonstrated distance from perinucleolar membrane is heavily influenced by defect of adaptor function of HEATR5B, by fluorescent microscopy and statistical analysis. Next they made HEATR5B depleted Drosophila embryo by CRSPR-CAS9. They proved its influence on AP1 trafficking to Golgi, which is another novel finding of this study, consistent with the case of U2OS cells.

In general the whole study proved importance of HEATR5 proteins on AP1 trafficking. Many data are presented in convincing way and carefully statistically analyzed. This work will attract attention of wide audience from the field of cytoskeleton, motor proteins and membrane trafficking. After addressing a few points, the manuscript will be ready for publication.

Reviewer #3 (Evidence, reproducibility and clarity (Required)):

Summary:

The goal of the authors is to identify dynein regulators which control how dynein and dynactin complexes orchestrate trafficking of diverse cargoes. To do so, the authors have performed a well thought proteomic screen for novel interacting proteins of the dynein tail potentially enhanced by dynactin. These pull-down experiments identified about 50 new dynein tail-interacting proteins, many of which were enhanced by dynactin.

The authors focused on one candidate, HEATR5B, because it was robustly isolated from the screens and its association with the dynein tail was stimulated by exogenous dynactin. HEATR5B is known to interact with AP1 complex, as adaptors that orchestrate cargo loading of clathrin-coated vesicles from intracellular membranes.

The authors further show that HEATR5B complexes with endogenous dynactin and dynein as reveal by immuno precipitation from human cells extracts and can interact with both complexes directly. Then by using Hela cell line stably expressing GFP-HEATR5B, they show that HEATR5B is selectively enriched on the AP1 structure, some of which can be subjected to long-distance transport. They provide evidences that a large proportion of the HEATR5B-positive structures are associated with endosomal recycling membranes, as revealed by colocalization with RAB11A. They further show that the HEATR5B/ AP1 and HEATR5B/ RAB11 membrane structures show similar dynamics, indicating that HEATR5B associate with endosomal membranes that are capable of directed movement. SiRNA depletion of DYNC1H1 reveals that dynein promotes retrograde trafficking of AP-1 associated endosomal membranes.

The authors then investigate the contribution of HEATR5B to AP1-associated membrane trafficking by CRIPR/cas9-mediated mutagenesis in human U2OS cells that disrupt HEATR5B protein expression. They provide evidences that in HEATR5B mutant cells, there is a reduction in the amount of AP1 signal associated with RAB11A-positive structures indicating that disrupting HEATR5B reduces the association of AP1with endosomal membranes. This indicates that HEATR5B promotes AP1 recruitment to endosomal membranes.

HEATR5B overexpression in U2OS cells increased perinuclear clustering of Rab11A/AP1/dynactin-associated membrane, suggesting that HEATR5B can stimulate retrograde trafficking of AP1-associated endosomal membranes by dynein- dynactin.

To assess the importance of HEATR5B function at the organismal level, as well as in polarized cell type the authors investigate its function in Drosophila in which there is a single HEATR5B homologue (Heatr5). They generated via crisper an Heatr5 mutant strain. Heatr5 homozygous mutants are zygotic lethal that died in second larval instar stage. They further provide evidence by investigating nos-cas9 gRNA-Hr51+2 mothers, that Heatr5 plays maternal function essential for embryogenesis. They further show that in early embryos from nos-cas9 gRNA-Hr5 females AP1 puncta are strongly reduced and dimmer.

Next, to understand the effect of Heatr5 disruption on AP1-based trafficking in Drosophila, they used the syncytial blastoderm embryo in which the microtubule cytoskeleton is highly polarized with less apically nucleated ends above the nuclei and more basally extended ends.

In this system, the activity of minus end-directed motor, such as dynein, and minus end-directed motor, such as kinesin, can be distinguished by the direction of cargo movement.

By injecting AP1 antibodies into wild-type and Heatr5 mutant embryos, they provide evidence that AP1 undergoes net apical transport in the Drosophila embryo and that this process is strongly promoted by Heatr5. They further show that this process is microtubule and dynein dependent and that Heatr5 selectively promotes dynein-mediated transport of AP1 structures in the embryo.

They then show that Heatr5-dependent AP1 trafficking pathways in the embryo involves the endosomal and Golgi membranes and that Heatr5 is also required for Golgi organization.

Major Comments

This study is very comprehensive and multi-scale. It ranges from the identification of a dynein motor adaptor for membrane trafficking by a proteomic screen, to its functional characterization in human cells and then during development with Drosophila embryo as model organism.

The data are of high quality and are supported by very convincing quantitative analyses. The results are conclusive and the experiments have been carried out and presented in a very constructive way. This combination makes the manuscript very interesting.

Minor comments

HEATR5B overexpression in U2OS cells increased perinuclear clustering of Rab11A/AP1/dynactin-associated membrane. To which compartment are these vesicles directed and associated, the Golgi apparatus? Could the authors show which compartment it is?

We have performed a new experiment assessing if the vesicles associate with the Golgi apparatus, as suggested. The data (documented in Appendix Figure S5 and introduced from line 336) show that the vesicles are indeed in the vicinity of the TGN (stained with TGN46 antibodies). As the TGN is positioned near the MTOC, where minus ends are enriched, we cannot say that the vesicles are definitely associated with the TGN. However, our observations are consistent with the proposed role of HEATR5B in retrograde trafficking of endosomal membranes. We thank the reviewer for the suggestion to investigate this matter further.

Reviewer #3 (Significance (Required)):

This study is important in two aspects. Firstly, it has identified HEATR5B as a new adaptor of the dynein motor for intracellular membrane trafficking. It is important to mention that this motor is involved in many transport processes and it is still unclear how a single motor orchestrates the traffic of so many cargoes.

Second, this work shed new light on the retrograde trafficking from endosomal material to the Golgi apparatus, in particular with HEATR5B, a known interactor of the AP1 clathrin adapter complex. This study highlights a role of HEATR5B in a novel dynein-based process for

retrograde trafficking of AP1-associated endosomal vesicle to the Golgi apparatus. It also indicates that HEATR5B promotes association of AP1 with endosomal membrane in a dynein-independent manner.

This work is particularly important for the cell biology field.

1st Revision - Editorial Decision 19th Sep 2023

Dear Simon,

We have now received re-review reports from all three referees. As you will see, you have addressed their concerns satisfactorily. Before I can finally accept the manuscript though, there are some remaining editorial points which need to be addressed. In this regard would you please:

address the specific issues in the data edited manuscript; these are listed as comments in the word document among the manuscript files,
remove legends from manuscript file and include them in each corresponding Excel file as a separate sheet, and
include page numbers in the Appendix 1 table of contents.

We include a synopsis of the paper and visual summary figure on our website (see http://emboj.embopress.org/). Please provide me with a general summary statement and 3-5 bullet points that capture the key findings of the paper. The summary figure size should be 550 pixels wide by [200-400] pixels high. You can also use something from the figures if that is easier.

EMBO Press is an editorially independent publishing platform for the development of EMBO scientific publications.

Best wishes,

William

Yours sincerely,

William Teale, PhD
Editor
The EMBO Journal
w.teale@embojournal.org

Instructions for preparing your revised manuscript:

Please check that the title and abstract of the manuscript are brief, yet explicit, even to non-specialists.

When assembling figures, please refer to our figure preparation guideline in order to ensure proper formatting and readability in print as well as on screen:
https://bit.ly/EMBOPressFigurePreparationGuideline
See also figure legend guidelines: https://www.embopress.org/page/journal/14602075/authorguide#figureformat

IMPORTANT: When you send the revision we will require
- a point-by-point response to the referees' comments, with a detailed description of the changes made (as a word file).
- a word file of the manuscript text.
- individual production quality figure files (one file per figure)
- a complete author checklist, which you can download from our author guidelines (https://www.embopress.org/page/journal/14602075/authorguide).
- Expanded View files (replacing Supplementary Information)
Please see out instructions to authors
https://www.embopress.org/page/journal/14602075/authorguide#expandedview

Please remember: Digital image enhancement is acceptable practice, as long as it accurately represents the original data and conforms to community standards. If a figure has been subjected to significant electronic manipulation, this must be noted in the figure legend or in the 'Materials and Methods' section. The editors reserve the right to request original versions of figures and the original images that were used to assemble the figure.

Further information is available in our Guide For Authors: https://www.embopress.org/page/journal/14602075/authorguide

We realize that it is difficult to revise to a specific deadline. In the interest of protecting the conceptual advance provided by the work, we recommend a revision within 3 months (18th Dec 2023). Please discuss the revision progress ahead of this time with

the editor if you require more time to complete the revisions. Use the link below to submit your revision:

https://emboj.msubmit.net/cgi-bin/main.plex

Referee #1:

Madan and colleagues revised their manuscript "HEATR5B associates with dynein-dynactin and promotes motility of AP1-bound endosomal membranes". Most of the reviewers' points were addressed, either by additional experiments rewriting. Otherwise they explained why it is not possible and toned down conclusion. The manuscript is now acceptable for publication in the EMBO Journal.

Referee #2:

In this revised manuscript, Vanesa Madan and colleagues did nicely improve their manuscript and modified the figures accordingly.

The authors responded satisfactorily to the point I had raised. They provided detailed information to the comments and questions submitted by the reviewers.

The manuscript has improved since the last submission and I would recommend its publication in EMBO Journal.

Referee #3:

I am satisfied with the responses to my comments and the revisions of the text. The article in its present form can be published.

All editorial and formatting issues were resolved by the authors.

2nd Revision - Editorial Decision 26th Sep 2023

Dear Simon,

I am pleased to inform you that your manuscript has been accepted for publication in the EMBO Journal.

Congratulations on a really insightful study!

Please note that it is EMBO Journal policy for the transcript of the editorial process (containing referee reports and your response letter) to be published as an online supplement to each paper. If you do NOT want this, you will need to inform the Editorial Office via email immediately. More information is available here: https://www.embopress.org/page/journal/14602075/authorguide#transparentprocess

Your manuscript will be processed for publication in the journal by EMBO Press. Manuscripts in the PDF and electronic editions of The EMBO Journal will be copy edited, and you will be provided with page proofs prior to publication. Please note that supplementary information is not included in the proofs.

You will be contacted by Wiley Author Services to complete licensing and payment information. The required 'Page Charges Authorization Form' is available here: https://www.embopress.org/pb-assets/embo-site/tej_apc.pdf - please download and complete the form and return to embopressproduction@wiley.com

EMBO Press participates in many Publish and Read agreements that allow authors to publish Open Access with reduced/no publication charges. Check your eligibility: https://authorservices.wiley.com/author-resources/Journal-Authors/open-access/affiliation-policies-payments/index.html

Should you be planning a Press Release on your article, please get in contact with embojournal@wiley.com as early as possible, in order to coordinate publication and release dates.

If you have any questions, please do not hesitate to call or email the Editorial Office. Thank you for your contribution to The EMBO Journal.

Best wishes,

William

William Teale, PhD
Editor
The EMBO Journal
w.teale@embojournal.org

** Click here to be directed to your login page: https://emboj.msubmit.net

EMBO Press Author Checklist

Corresponding Author Name: SIMON BULLOCK	
Journal Submitted to: The EMBO Journal	
Manuscript Number: EMBOJ-2023-114473	

USEFUL LINKS FOR COMPLETING THIS FORM
The EMBO Journal - Author Guidelines
EMBO Reports - Author Guidelines
Molecular Systems Biology - Author Guidelines
EMBO Molecular Medicine - Author Guidelines

Reporting Checklist for Life Science Articles (updated January
This checklist is adapted from Materials Design Analysis Reporting (MDAR) Checklist for Authors. MDAR establishes a minimum set of requirements in transparent reporting in the life sciences (see Statement of Task: 10.31222/osf.io/9sm4x). Please follow the journal's guidelines in preparing your
Please note that a copy of this checklist will be published alongside your article.

Abridged guidelines for figures
1. Data
The data shown in figures should satisfy the following conditions:
- the data were obtained and processed according to the field's best practice and are presented to reflect the results of the experiments in an accurate and unbiased manner.
- ideally, figure panels should include only measurements that are directly comparable to each other and obtained with the same assay.
- plots include clearly labeled error bars for independent experiments and sample sizes. Unless justified, error bars should not be shown for technical
- if n<5, the individual data points from each experiment should be plotted. Any statistical test employed should be justified.
- Source Data should be included to report the data underlying figures according to the guidelines set out in the authorship guidelines on Data

2. Captions
Each figure caption should contain the following information, for each panel where they are relevant:
- a specification of the experimental system investigated (eg cell line, species name).
- the assay(s) and method(s) used to carry out the reported observations and measurements.
- an explicit mention of the biological and chemical entity(ies) that are being measured.
- an explicit mention of the biological and chemical entity(ies) that are altered/varied/perturbed in a controlled manner.
- the exact sample size (n) for each experimental group/condition, given as a number, not a range;
- a description of the sample collection allowing the reader to understand whether the samples represent technical or biological replicates (including how many animals, litters, cultures, etc.).
- a statement of how many times the experiment shown was independently replicated in the laboratory.
- definitions of statistical methods and measures:
  - common tests, such as t-test (please specify whether paired vs. unpaired), simple χ2 tests, Wilcoxon and Mann-Whitney tests, can be unambiguously identified by name only, but more complex techniques should be described in the methods section;
  - are tests one-sided or two-sided?
  - are there adjustments for multiple comparisons?
  - exact statistical test results, e.g., P values = x but not P values < x;
  - definition of 'center values' as median or average;
  - definition of error bars as s.d. or s.e.m.

Please complete ALL of the questions below.
Select "Not Applicable" only when the requested information is not relevant for your study.

Materials

**Newly Created Materials**	**Information included in the manuscript?**	**In which section is the information available?** (Reagents and Tools Table, Materials and Methods, Figures, Data Availability Section)
New materials and reagents need to be available; do any restrictions apply?	Yes	All new materials and reagents are described in the Materials and Methods and there are no restrictions on availability other than standard academic MTAs, if applicable

**Antibodies**	**Information included in the manuscript?**	**In which section is the information available?** (Reagents and Tools Table, Materials and Methods, Figures, Data Availability Section)
For **antibodies** provide the following information:<br>- Commercial antibodies: RRID (if possible) or supplier name, catalogue number and or/clone number<br>- Non-commercial: RRID or citation	Yes	Supplier names and catalogue numbers provided in Materials and Methods

**DNA and RNA sequences**	**Information included in the manuscript?**	**In which section is the information available?** (Reagents and Tools Table, Materials and Methods, Figures, Data Availability Section)
**Short novel DNA or RNA including primers, probes**: provide the sequences.	Yes	Sequences of gRNAs and oligonucleotides used to generate them are provided in the Materials and Methods and Appendix (Appendix figures S3, S6, S7, S9 and Table S4)

**Cell materials**	**Information included in the manuscript?**	**In which section is the information available?** (Reagents and Tools Table, Materials and Methods, Figures, Data Availability Section)
**Cell lines:** Provide species information, strain. Provide accession number in repository **OR** supplier name, catalog number, clone number, and/**OR** RRID.	Yes	Information on (non-commercial) sources of cell lines is included in the Materials and Methods
**Primary cultures:** Provide species, strain, sex of origin, genetic modification status.	Not Applicable	
Report if the cell lines were recently **authenticated** (e.g., by STR profiling) and tested for mycoplasma contamination.	Yes	Information on mycoplasma testing is included in the Materials and Methods

**Experimental animals**	**Information included in the manuscript?**	**In which section is the information available?** (Reagents and Tools Table, Materials and Methods, Figures, Data Availability Section)
**Laboratory animals or Model organisms:** Provide species, strain, sex, age, genetic modification status. Provide accession number in repository **OR** supplier name, catalog number, clone number, **OR** RRID.	Yes	Information on Drosophila strains is provided in the Materials and Methods, including stock centre numbers when applicable
**Animal observed in or captured from the field:** Provide species, sex, and age where possible.	Not Applicable	
Please detail **housing and husbandry conditions**.	Yes	This is detailed in the Materials and Methods

**Plants and microbes**	**Information included in the manuscript?**	**In which section is the information available?** (Reagents and Tools Table, Materials and Methods, Figures, Data Availability Section)
**Plants:** provide species and strain, ecotype and cultivar where relevant, unique accession number if available, and source (including location for collected wild specimens).	Not Applicable	
**Microbes:** provide species and strain, unique accession number if available, and source.	Not Applicable	

**Human research participants**	**Information included in the manuscript?**	**In which section is the information available?** (Reagents and Tools Table, Materials and Methods, Figures, Data Availability Section)
If collected and within the bounds of privacy constraints report on age, sex and gender or ethnicity for all study participants.	Not Applicable	

**Core facilities**	**Information included in the manuscript?**	**In which section is the information available?** (Reagents and Tools Table, Materials and Methods, Figures, Data Availability Section)
If your work benefited from core facilities, was their service mentioned in the acknowledgments section?	Yes	Acknowledgements section

Design

Study protocol	Information included in the manuscript?	In which section is the information available? (Reagents and Tools Table, Materials and Methods, Figures, Data Availability Section)
If study protocol has been **pre-registered, provide DOI in the manuscript**. For clinical trials, provide the trial registration number **OR** cite DOI.	Not Applicable	
Report the **clinical trial registration number** (at ClinicalTrials.gov or equivalent), where applicable.	Not Applicable	

Laboratory protocol	Information included in the manuscript?	In which section is the information available? (Reagents and Tools Table, Materials and Methods, Figures, Data Availability Section)
Provide DOI OR other citation details if **external detailed step-by-step protocols** are available.	Not Applicable	

Experimental study design and statistics	Information included in the manuscript?	In which section is the information available? (Reagents and Tools Table, Materials and Methods, Figures, Data Availability Section)
Include a statement about **sample size** estimate even if no statistical methods were used.	Not Applicable	
Were any steps taken to minimize the effects of subjective bias when allocating animals/samples to treatment (e.g. **randomization procedure**)? If yes, have they been described?	Not Applicable	
Include a statement about **blinding** even if no blinding was done.	Yes	Materials and Methods
Describe **inclusion/exclusion criteria** if samples or animals were excluded from the analysis. Were the criteria pre-established?<br><br>If sample or data points were omitted from analysis, report if this was due to attrition or intentional exclusion and provide justification.	Not Applicable	
For every figure, are **statistical tests** justified as appropriate? Do the data meet the assumptions of the tests (e.g., normal distribution)? Describe any methods used to assess it. Is there an estimate of variation within each group of data? Is the variance similar between the groups that are being statistically compared?	Yes	The Materials and Methods concludes with a section that covers this aspect of the work

Sample definition and in-laboratory replication	Information included in the manuscript?	In which section is the information available? (Reagents and Tools Table, Materials and Methods, Figures, Data Availability Section)
In the figure legends: state number of times the experiment was **replicated** in laboratory.	Yes	
In the figure legends: define whether data describe **technical or biological replicates**.	Yes	

Ethics

Ethics	Information included in the manuscript?	In which section is the information available? (Reagents and Tools Table, Materials and Methods, Figures, Data Availability Section)
Studies involving **human participants**: State details of **authority granting ethics approval** (IRB or equivalent committee(s), provide reference number for approval.	Not Applicable	
Studies involving **human participants**: Include a statement confirming that **informed consent** was obtained from all subjects and that the experiments conformed to the principles set out in the WMA Declaration of Helsinki and the Department of Health and Human Services Belmont Report.	Not Applicable	
Studies involving **human participants:** For publication of **patient photos**, include a statement confirming that consent to publish was obtained.	Not Applicable	
Studies involving experimental **animals**: State details of **authority granting ethics approval** (IRB or equivalent committee(s), provide reference number for approval. Include a statement of compliance with ethical regulations.	Not Applicable	
Studies involving **specimen and field samples:** State if relevant **permits** obtained, provide details of authority approving study; if none were required, explain why.	Not Applicable	

Dual Use Research of Concern (DURC)	Information included in the manuscript?	In which section is the information available? (Reagents and Tools Table, Materials and Methods, Figures, Data Availability Section)
Could your study fall under dual use research restrictions? Please check biosecurity documents and list of **select agents and toxins** (CDC): https://www.selectagents.gov/sat/list.htm	Not Applicable	
If you used a select agent, is the security level of the lab appropriate and reported in the manuscript?	Not Applicable	
If a study is subject to dual use research of concern regulations, is the name of the **authority granting approval and reference number** for the regulatory approval provided in the manuscript?	Not Applicable	

Reporting

The MDAR framework recommends adoption of discipline-specific guidelines, established and endorsed through community initiatives. Journals have their own policy about requiring specific guidelines and recommendations to complement MDAR.

Adherence to community standards	Information included in the manuscript?	In which section is the information available? (Reagents and Tools Table, Materials and Methods, Figures, Data Availability Section)
State if relevant guidelines or checklists (e.g., **ICMJE, MIBBI, ARRIVE, PRISMA**) have been followed or provided.	Not Applicable	
For **tumor marker prognostic studies,** we recommend that you follow the **REMARK** reporting guidelines (see link list at top right). See author guidelines, under 'Reporting Guidelines'. Please confirm you have followed these guidelines.	Not Applicable	
For **phase II and III randomized controlled trials**, please refer to the **CONSORT** flow diagram (see link list at top right) and submit the CONSORT checklist (see link list at top right) with your submission. See author guidelines, under 'Reporting Guidelines'. Please confirm you have submitted this list.	Not Applicable	

Data Availability

Data availability	Information included in the manuscript?	In which section is the information available? (Reagents and Tools Table, Materials and Methods, Figures, Data Availability Section)
Have **primary datasets** been deposited according to the journal's guidelines (see 'Data Deposition' section) and the respective accession numbers provided in the Data Availability Section?	Yes	Data availability section
Were **human clinical and genomic datasets** deposited in a public access-controlled repository in accordance to ethical obligations to the patients and to the applicable consent agreement?	Not Applicable	
Are **computational models** that are central and integral to a study available without restrictions in a machine-readable form? Were the relevant accession numbers or links provided?	Not Applicable	
If publicly available data were reused, provide the respective **data citations in the reference list**.	Not Applicable	
