# Supplementary material for: HEATR5B associates with dynein‐dynactin and promotes motility of AP1‐bound endosomal membranes
Source: EMBO J. 2023 Oct 24;42(23):e114473. doi: 10.15252/embj.2023114473 (PMC10690479; doi:10.15252/embj.2023114473)
Supplement: Supplementary file 9 — Movie EV7 [file EMBJ-42-e114473-s014.zip › Movie EV7/Movie EV7.docx]

**Movie EV7. Behaviour of injected Alexa555-secondary antibody/AP1γ primary antibody conjugates in wild-type embryo preinjected with colcemid and after inactivation of colcemid with UV light.** ‘UV’ indicates brief period of exposure to UV light (1 min 5 sec into the duration of the movie). Scale bar, 10 μm. Movie is representative of injections into four embryos.
